# Supplementary figures and images for: Resistance characterization and transcriptomic analysis of imipenem-induced drug resistance in Escherichia coli
Source: PeerJ. 2024 Nov 29;12:e18572. doi: 10.7717/peerj.18572 (PMC11610472; doi:10.7717/peerj.18572)

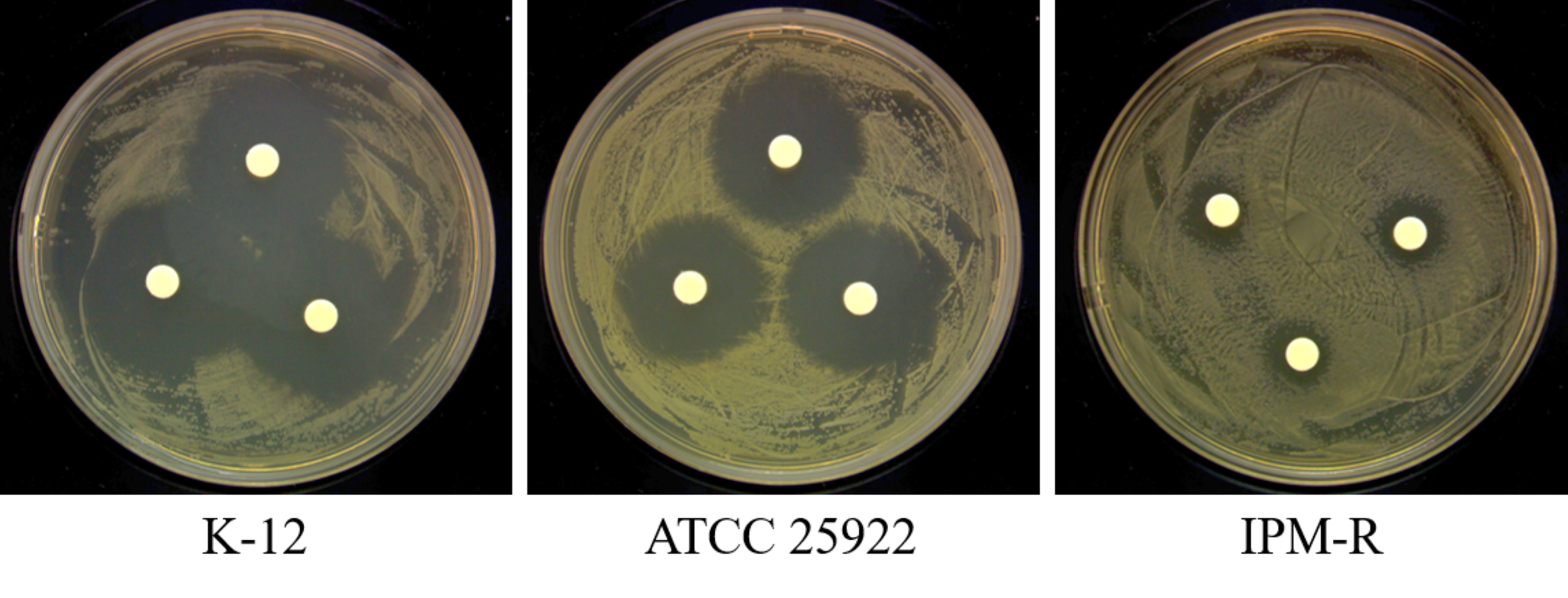

Supplement: Figure S1 [file peerj-12-18572-s001.png]

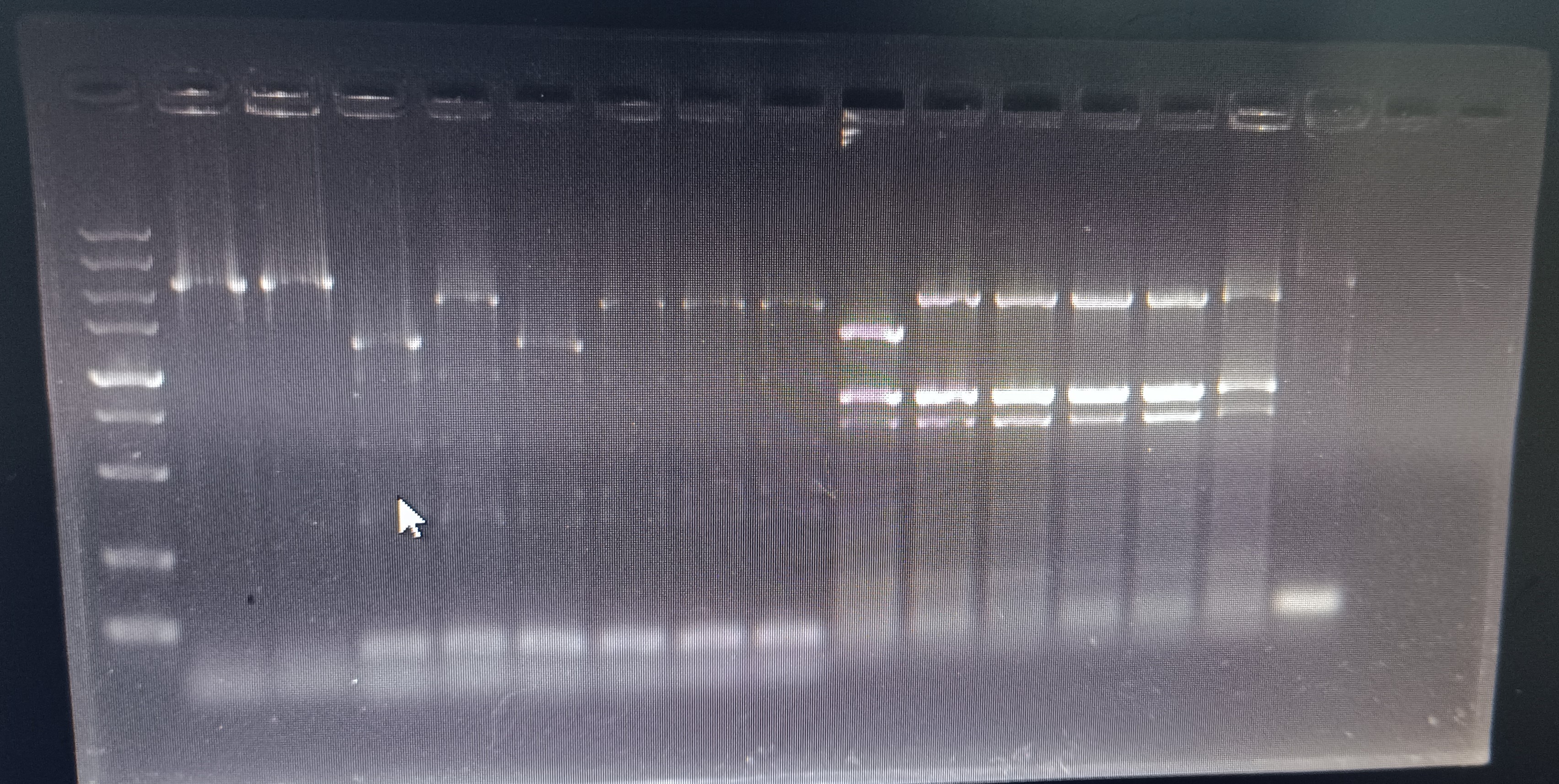

Supplement: Figure S2 — Column 15 is the wild-type strain control, and the remaining columns are knockout strain verifications. The strains used in sequencing and experiments are in column 3 (the 5000 bp marker in the left column is not considered). The primers used were mdtC-2 listed in Table S6. [file peerj-12-18572-s002.jpg]

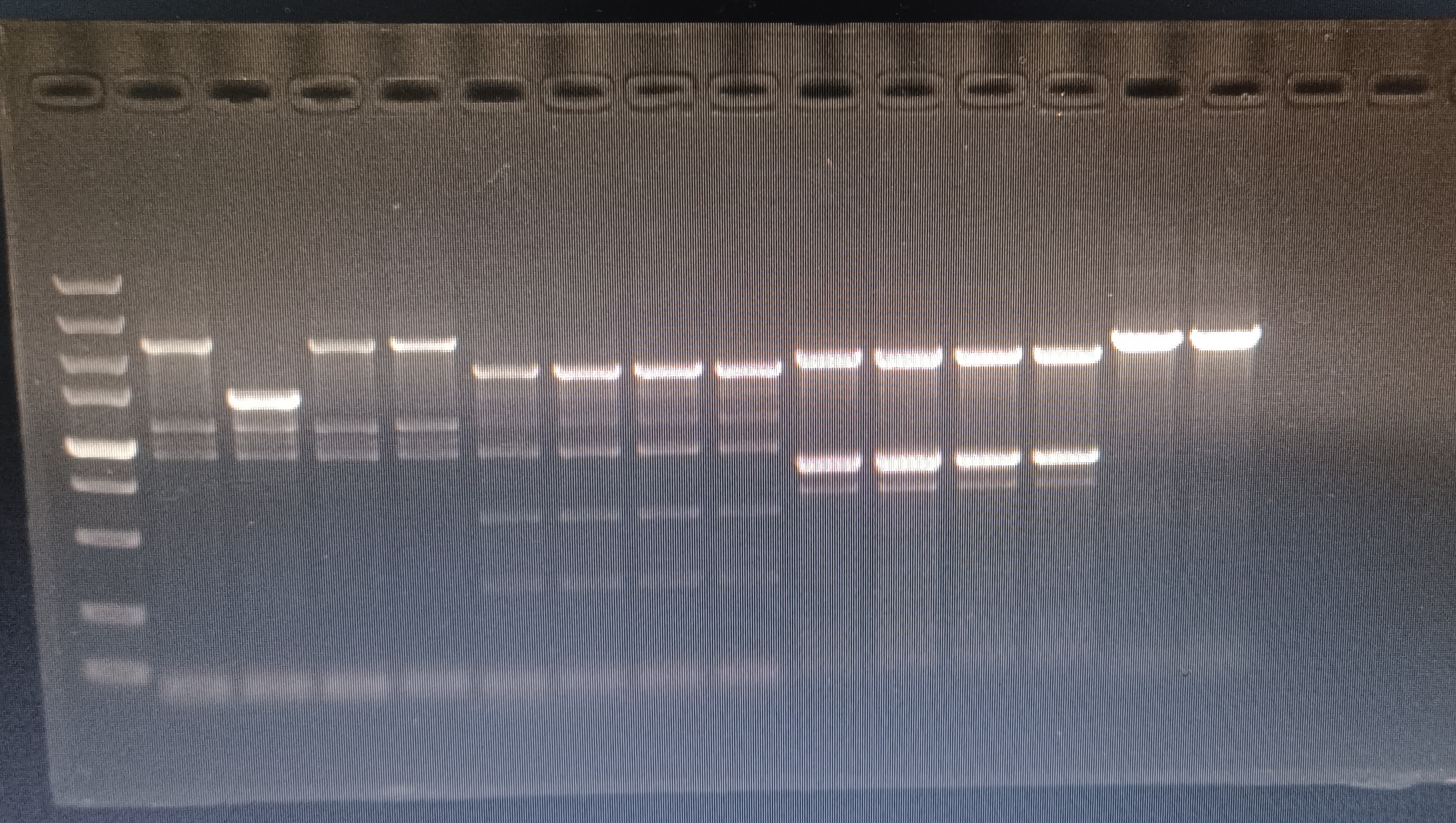

Supplement: Figure S3 — The 1st column is the verification of mdtD in the wild-type strain, the 2–3 columns are the verification of the mdtD gene deletion strain; the 5th column is the verification of the mdtC-1 primer in the wild-type strain, and the 6–8 columns are mdtC-1 Verification of primers in gene deletion strains; column 9 is the verification of mdtC-2 primers in wild-type strains, columns 10–12 are verification of mdtC-2 primers in gene deletion strains (the 5000 bp marker in the left column is not considered). [file peerj-12-18572-s003.jpg]

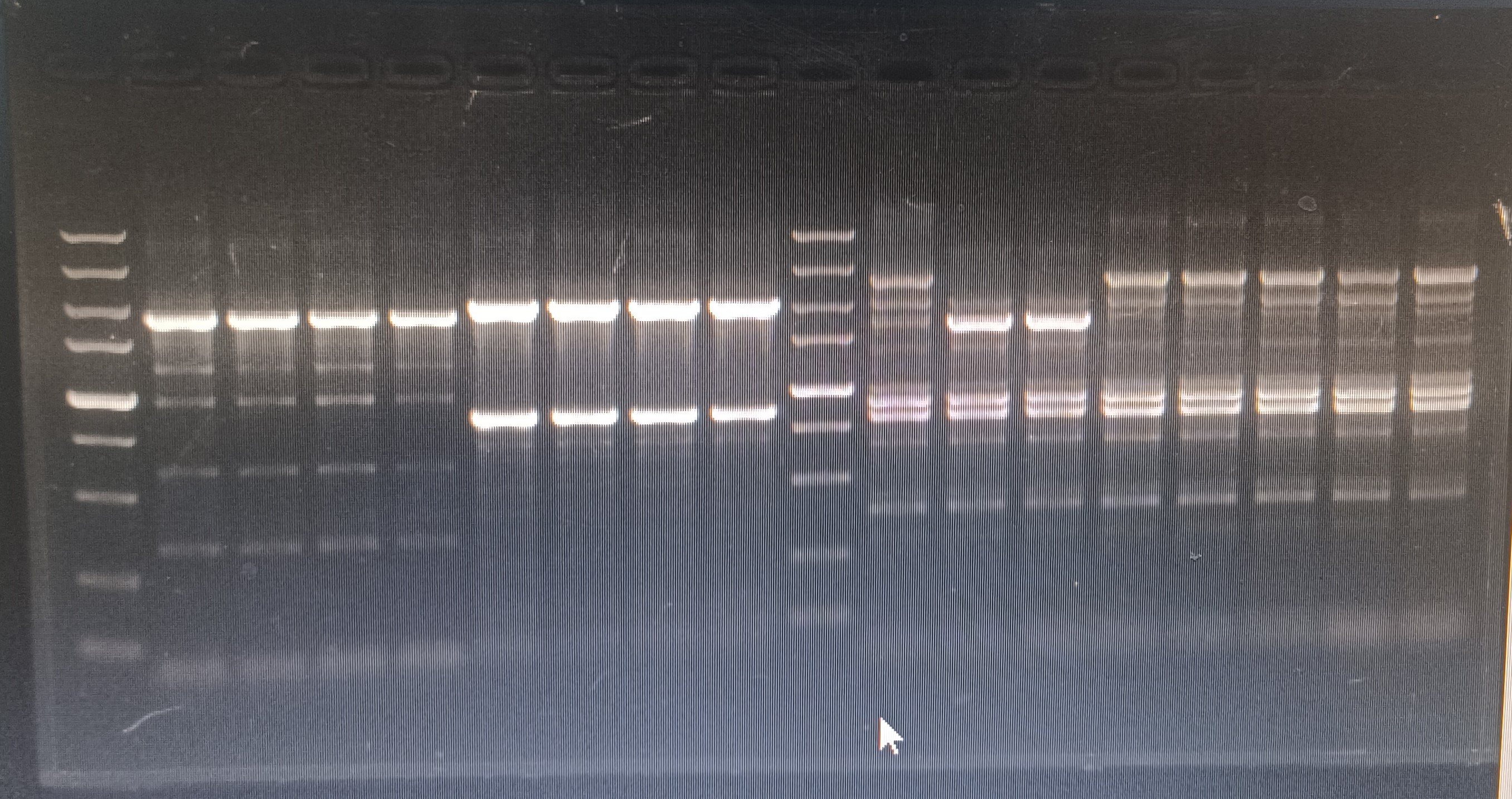

Supplement: Figure S4 — The 1st column is the verification of mdtC-1 in the wild-type strain, the 2-3 columns are the verification of the mdtC-1 gene deletion strain; the 5th column is the verification of the mdtC-2 in the wild-type strain, and the 6-8 columns are verification of mdtC-2 in gene deletion strains; column 9 is the verification of macB primers in wild-type strains, columns 10-16 are verification of mdtC-2 primers in gene deletion strains(the 5000 bp markers in the figure are not considered). [file peerj-12-18572-s004.jpg]

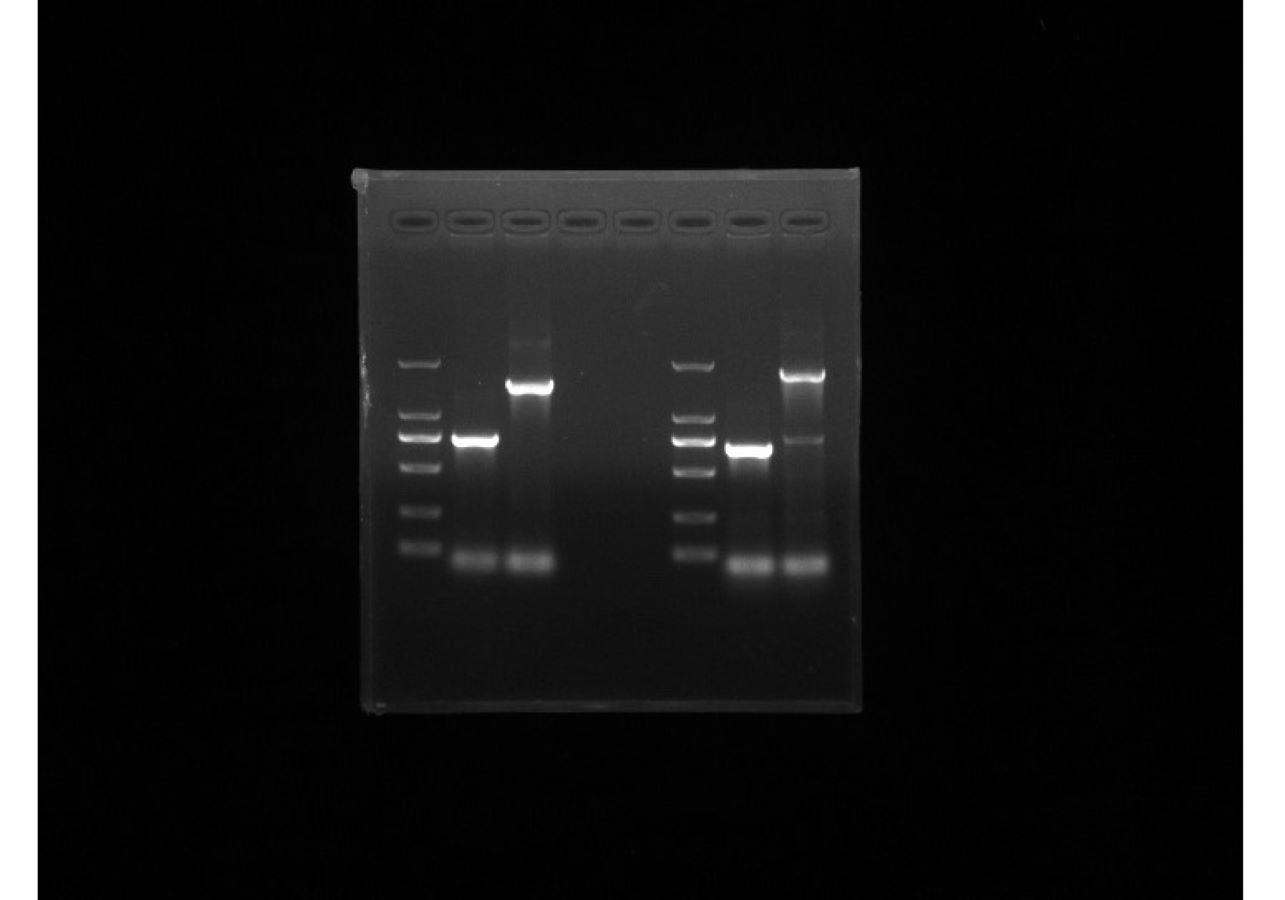

Supplement: Figure S5 — The 1st column is the verification of mdtE in the knockout strain, the 2nd column is the verification of mdtE in the wild-type strain; the 3rd column is the verification of mdtF in the knockout strain, and the 4th column is the verification of mdtF in the wild-type strain. [file peerj-12-18572-s005.jpg]
