# Supplementary material for: Resistance characterization and transcriptomic analysis of imipenem-induced drug resistance in Escherichia coli
Source: PeerJ. 2024 Nov 29;12:e18572. doi: 10.7717/peerj.18572 (PMC11610472; doi:10.7717/peerj.18572)
Supplement: Supplemental Information 1 [file peerj-12-18572-s006.pdf]

1. Imipenem inhibition zone test
2. PCR validation of *mdtC* knockout strains

   Column 15 is the wild-type strain control, and the remaining columns are knockout strain verifications. The strains used in sequencing and experiments are in column 3 (the 5000bp marker in the left column is not considered). The primers used were mdtC-2 listed in Table S6.
3. PCR validation of *mdtD* knockout strains

   The 1st column is the verification of *mdtD* in the wild-type strain, the 2-3 columns are the verification of the *mdtD* gene deletion strain; the 5th column is the verification of the *mdtC*-1 primer in the wild-type strain, and the 6-8 columns are *mdtC*-1 Verification of primers in gene deletion strains; column 9 is the verification of *mdtC*-2 primers in wild-type strains, columns 10-12 are verification of *mdtC*-2 primers in gene deletion strains(the 5000bp marker in the left column is not considered).
4. PCR validation of *macB* knockout strains

   The 1st column is the verification of *mdtC*-1 in the wild-type strain, the 2-3 columns are the verification of the *mdtC*-1 gene deletion strain; the 5th column is the verification of the *mdtC*-2 in the wild-type strain, and the 6-8 columns are verification of *mdtC*-2 in gene deletion strains; column 9 is the verification of *macB* primers in wild-type strains, columns 10-16 are verification of mdtC-2 primers in gene deletion strains(the 5000bp markers in the figure are not considered).

5. PCR validation of *mdtE* and *mdtF* knockout strains

   We knocked out both the *mdtE* and *mdtF* genes in the operon where *mdtF* is located, although only the *mdtF* gene deletion strain was used in this study. The 1st column is the verification of *mdtE* in the knockout strain, the 2nd column is the verification of *mdtE* in the wild-type strain; the 3rd column is the verification of *mdtF* in the knockout strain, and the 4th column is the verification of *mdtF* in the wild-type strain.
6. GO classification of differentially expressed genes
7. GO classification of differentially expressed genes
8. GO classification of differentially expressed genes
9. GO classification of differentially expressed genes
10. COG enrichment of differentially expressed genes
11. COG enrichment of differentially expressed genes
12. Enrichment of KEGG pathway for differentially expressed genes
13. Carbon fixation pathways
14. Carbon metabolism
15. Sulfur metabolism
16. Sulfur relay system
17. Nitrogen metabolism
18. Starch and sucrose metabolism
19. Glycolysis/Gluconeogenesis
20. Pentose phosphate pathway

21. Phosphotransferase system (PTS)
22. Fatty acid degradation
23. Citrate cycle (TCA)
24. Biosynthesis of amino acid
25. Arginine and proline metabolism
26. Arginine biosynthesis
27. Lysine biosynthesis
28. Valine, leucine and isoleucine biosynthesis
29. Tryptophan metabolism
30. Ribosome
31. Purine metabolism
32. Lipopolysaccharide biosynthesis
33. Peptidoglycan biosynthesis
34. ABC transporters
35. Phosphotransferase system (PTS)
36. Two-component system
37. Flagellar assembly
38. Quorum sensing
39. Biofilm formation
40. Bacterial chemotaxis

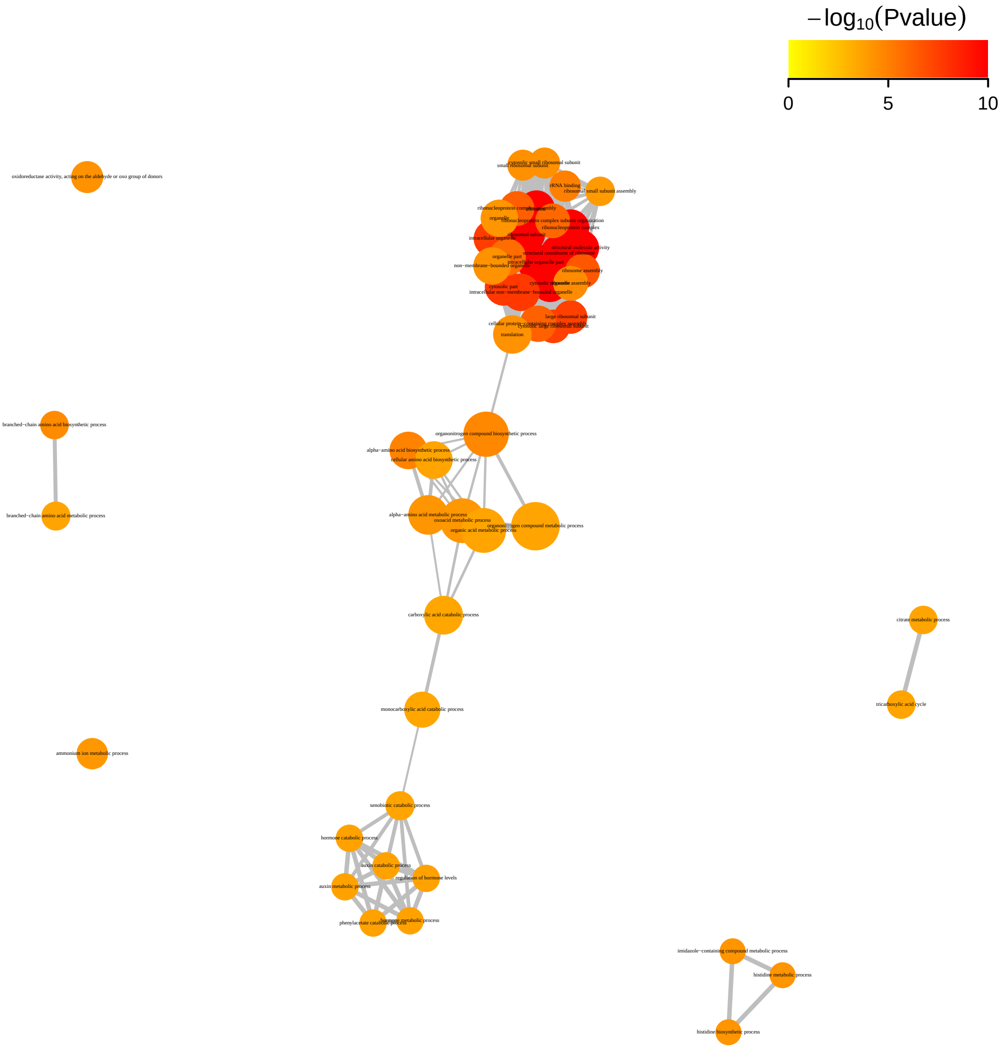

$-\log_{10}(\text{Pvalue})$
0
5
10
oxidoreductase activity, acting on the aldehyde or oxo group of donors
cytosolic small ribosomal subunit
small ribosomal subunit
rRNA binding
ribosomal small subunit assembly
ribonucleoprotein complex assembly
ribosome
organelle
ribonucleoprotein complex subunit organization
ribonucleoprotein complex
ribosomal subunit
intracellular organelle
structural molecule activity
structural constituent of ribosome
organelle part
intracellular organelle part
non−membrane−bounded organelle
ribosome assembly
organelle assembly
cytosolic part
intracellular non−membrane−bounded organelle
large ribosomal subunit
cellular protein−containing complex assembly
cytosolic large ribosomal subunit
translation
branched−chain amino acid biosynthetic process
branched−chain amino acid metabolic process
organonitrogen compound biosynthetic process
alpha−amino acid biosynthetic process
cellular amino acid biosynthetic process
alpha−amino acid metabolic process
oxoacid metabolic process
organic acid metabolic process
organonitrogen compound metabolic process
carboxylic acid catabolic process
citrate metabolic process
tricarboxylic acid cycle
monocarboxylic acid catabolic process
ammonium ion metabolic process
xenobiotic catabolic process
hormone catabolic process
auxin catabolic process
regulation of hormone levels
auxin metabolic process
phenylacetate catabolic process
hormone metabolic process
imidazole−containing compound metabolic process
histidine metabolic process
histidine biosynthetic process

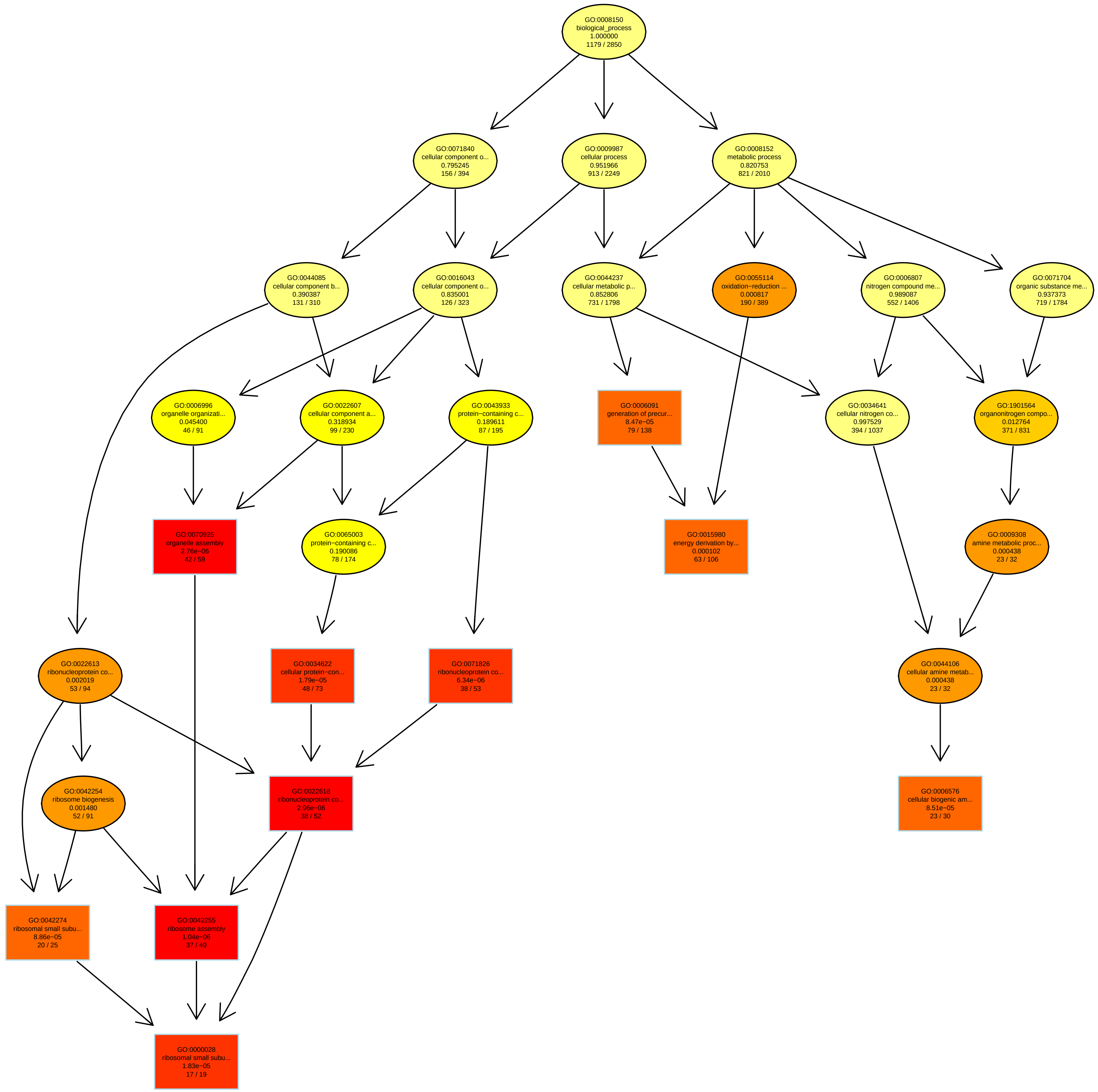

GO:0008150
biological_process
1.000000
1179 / 2850
GO:0071840
cellular component o...
0.795245
156 / 394
GO:0009987
cellular process
0.951966
913 / 2249
GO:0008152
metabolic process
0.820753
821 / 2010
GO:0044085
cellular component b...
0.390387
131 / 310
GO:0016043
cellular component o...
0.835001
126 / 323
GO:0044237
cellular metabolic p...
0.852806
731 / 1798
GO:0055114
oxidation−reduction ...
0.000817
190 / 389
GO:0006807
nitrogen compound me...
0.989087
552 / 1406
GO:0071704
organic substance me...
0.937373
719 / 1784
GO:0006996
organelle organizati...
0.045400
46 / 91
GO:0022607
cellular component a...
0.318934
99 / 230
GO:0043933
protein−containing c...
0.189611
87 / 195
GO:0006091
generation of precur...
8.47e−05
79 / 138
GO:0034641
cellular nitrogen co...
0.997529
394 / 1037
GO:1901564
organonitrogen compo...
0.012764
371 / 831
GO:0070925
organelle assembly
2.76e−06
42 / 59
GO:0065003
protein−containing c...
0.190086
78 / 174
GO:0015980
energy derivation by...
0.000102
63 / 106
GO:0009308
amine metabolic proc...
0.000438
23 / 32
GO:0022613
ribonucleoprotein co...
0.002019
53 / 94
GO:0034622
cellular protein−con...
1.79e−05
48 / 73
GO:0071826
ribonucleoprotein co...
6.34e−06
38 / 53
GO:0044106
cellular amine metab...
0.000438
23 / 32
GO:0042254
ribosome biogenesis
0.001480
52 / 91
GO:0022618
ribonucleoprotein co...
2.96e−06
36 / 52
GO:0006576
cellular biogenic am...
8.51e−05
23 / 30
GO:0042274
ribosomal small subu...
8.86e−05
20 / 25
GO:0042255
ribosome assembly
1.04e−06
37 / 49
GO:0000028
ribosomal small subu...
1.83e−05
17 / 19

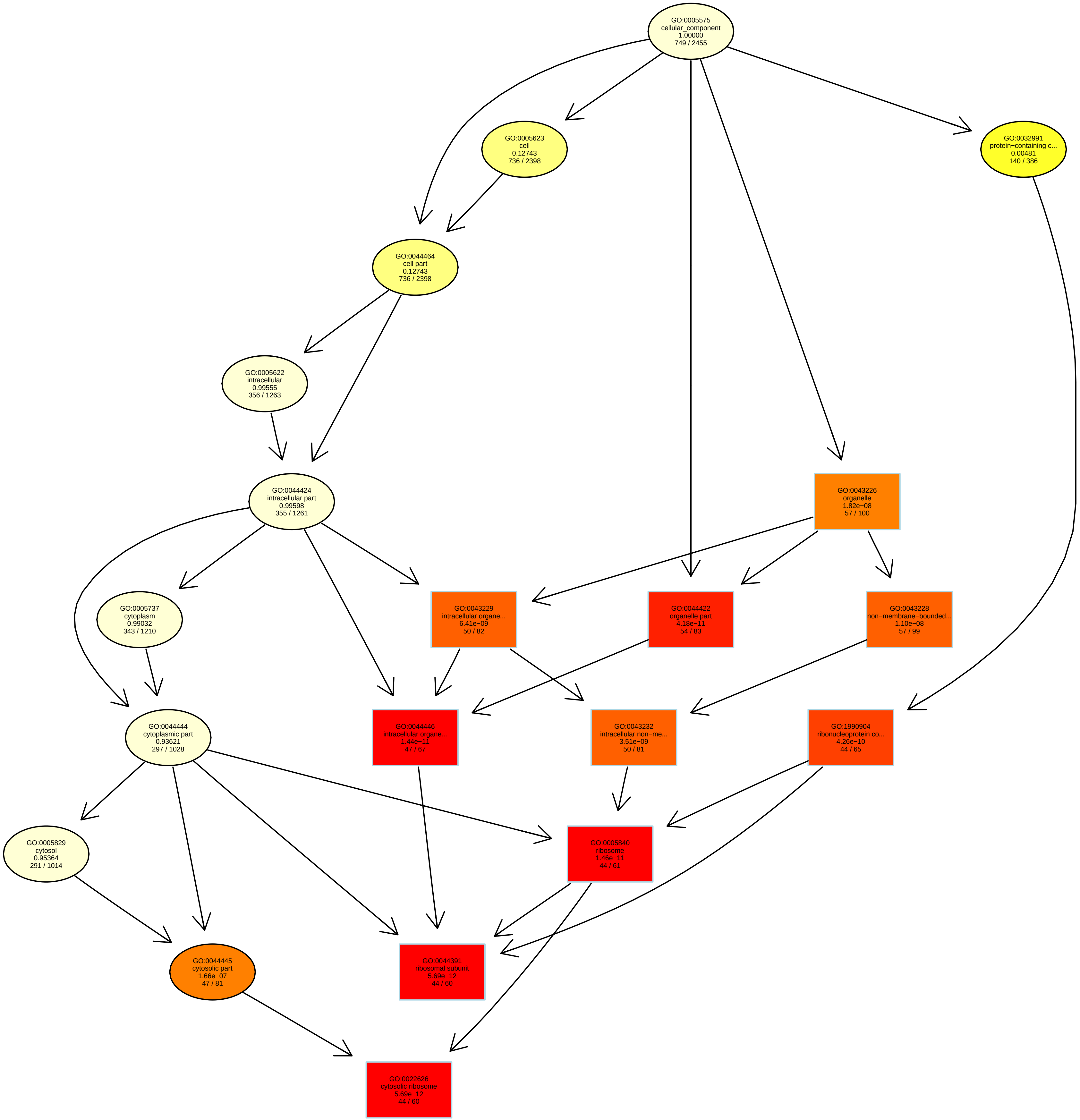
GO:0005575
cellular_component
1.00000
749 / 2455
GO:0005623
cell
0.12743
736 / 2398
GO:0032991
protein−containing c...
0.00481
140 / 386
GO:0044464
cell part
0.12743
736 / 2398
GO:0005622
intracellular
0.99555
356 / 1263
GO:0044424
intracellular part
0.99598
355 / 1261
GO:0043226
organelle
1.82e−08
57 / 100
GO:0005737
cytoplasm
0.99032
343 / 1210
GO:0043229
intracellular organe...
6.41e−09
50 / 82
GO:0044422
organelle part
4.18e−11
54 / 83
GO:0043228
non−membrane−bounded...
1.10e−08
57 / 99
GO:0044444
cytoplasmic part
0.93621
297 / 1028
GO:0044446
intracellular organe...
1.44e−11
47 / 67
GO:0043232
intracellular non−me...
3.51e−09
50 / 81
GO:1990904
ribonucleoprotein co...
4.26e−10
44 / 65
GO:0005829
cytosol
0.95364
291 / 1014
GO:0005840
ribosome
1.46e−11
44 / 61
GO:0044445
cytosolic part
1.66e−07
47 / 81
GO:0044391
ribosomal subunit
5.69e−12
44 / 60
GO:0022626
cytosolic ribosome
5.69e−12
44 / 60

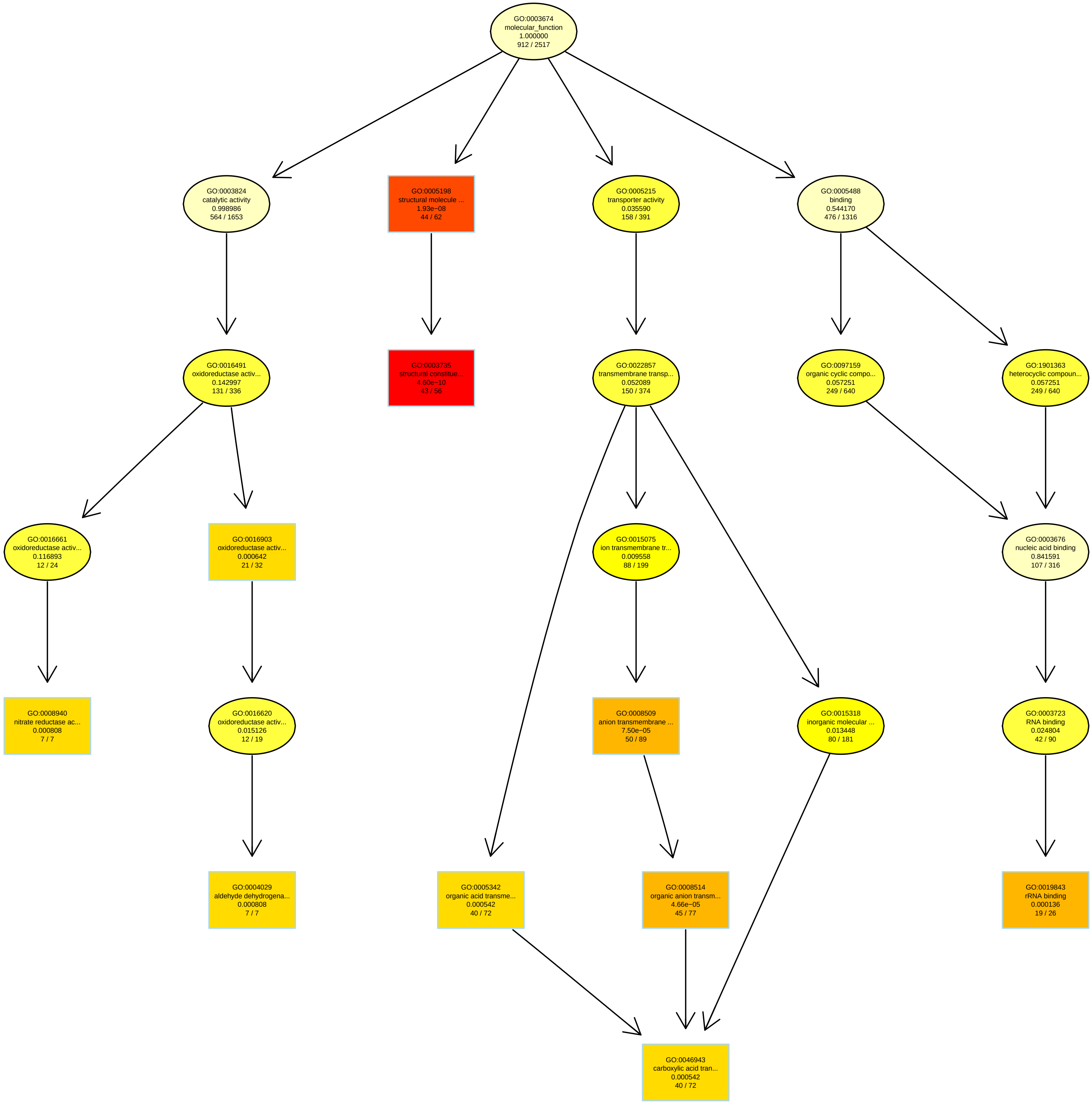
GO:0003674
molecular_function
1.000000
912 / 2517
GO:0003824
catalytic activity
0.998986
564 / 1653
GO:0005198
structural molecule ...
1.93e−08
44 / 62
GO:0005215
transporter activity
0.035590
158 / 391
GO:0005488
binding
0.544170
476 / 1316
GO:0016491
oxidoreductase activ...
0.142997
131 / 336
GO:0003735
structural constitue...
4.60e−10
43 / 56
GO:0022857
transmembrane transp...
0.052089
150 / 374
GO:0097159
organic cyclic compo...
0.057251
249 / 640
GO:1901363
heterocyclic compoun...
0.057251
249 / 640
GO:0016661
oxidoreductase activ...
0.116893
12 / 24
GO:0016903
oxidoreductase activ...
0.000642
21 / 32
GO:0015075
ion transmembrane tr...
0.009558
88 / 199
GO:0003676
nucleic acid binding
0.841591
107 / 316
GO:0008940
nitrate reductase ac...
0.000808
7 / 7
GO:0016620
oxidoreductase activ...
0.015126
12 / 19
GO:0008509
anion transmembrane ...
7.50e−05
50 / 89
GO:0015318
inorganic molecular ...
0.013448
80 / 181
GO:0003723
RNA binding
0.024804
42 / 90
GO:0004029
aldehyde dehydrogena...
0.000808
7 / 7
GO:0005342
organic acid transme...
0.000542
40 / 72
GO:0008514
organic anion transm...
4.66e−05
45 / 77
GO:0019843
rRNA binding
0.000136
19 / 26
GO:0046943
carboxylic acid tran...
0.000542
40 / 72

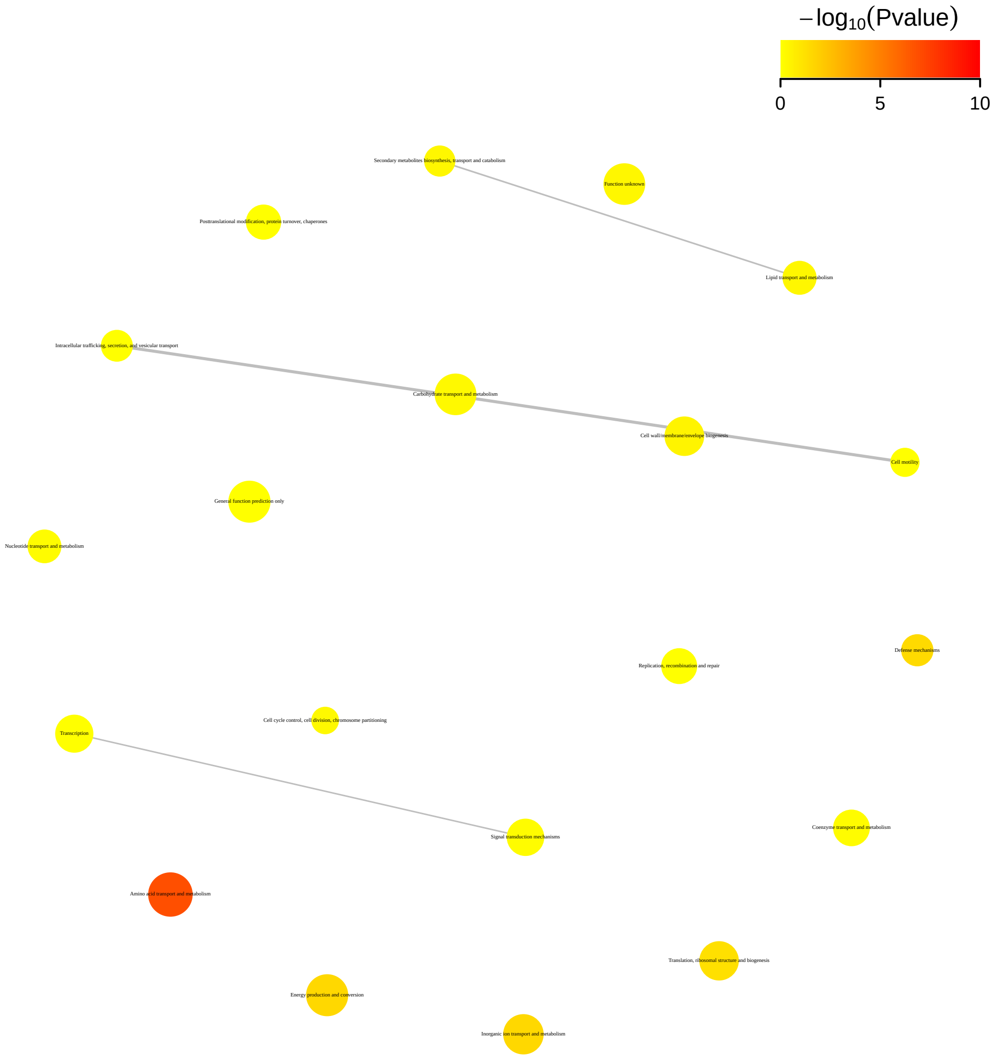

$-\log_{10}(\text{Pvalue})$
0
5
10
Secondary metabolites biosynthesis, transport and catabolism
Function unknown
Posttranslational modification, protein turnover, chaperones
Lipid transport and metabolism
Intracellular trafficking, secretion, and vesicular transport
Carbohydrate transport and metabolism
Cell wall/membrane/envelope biogenesis
Cell motility
General function prediction only
Nucleotide transport and metabolism
Defense mechanisms
Replication, recombination and repair
Cell cycle control, cell division, chromosome partitioning
Transcription
Signal transduction mechanisms
Coenzyme transport and metabolism
Amino acid transport and metabolism
Translation, ribosomal structure and biogenesis
Energy production and conversion
Inorganic ion transport and metabolism

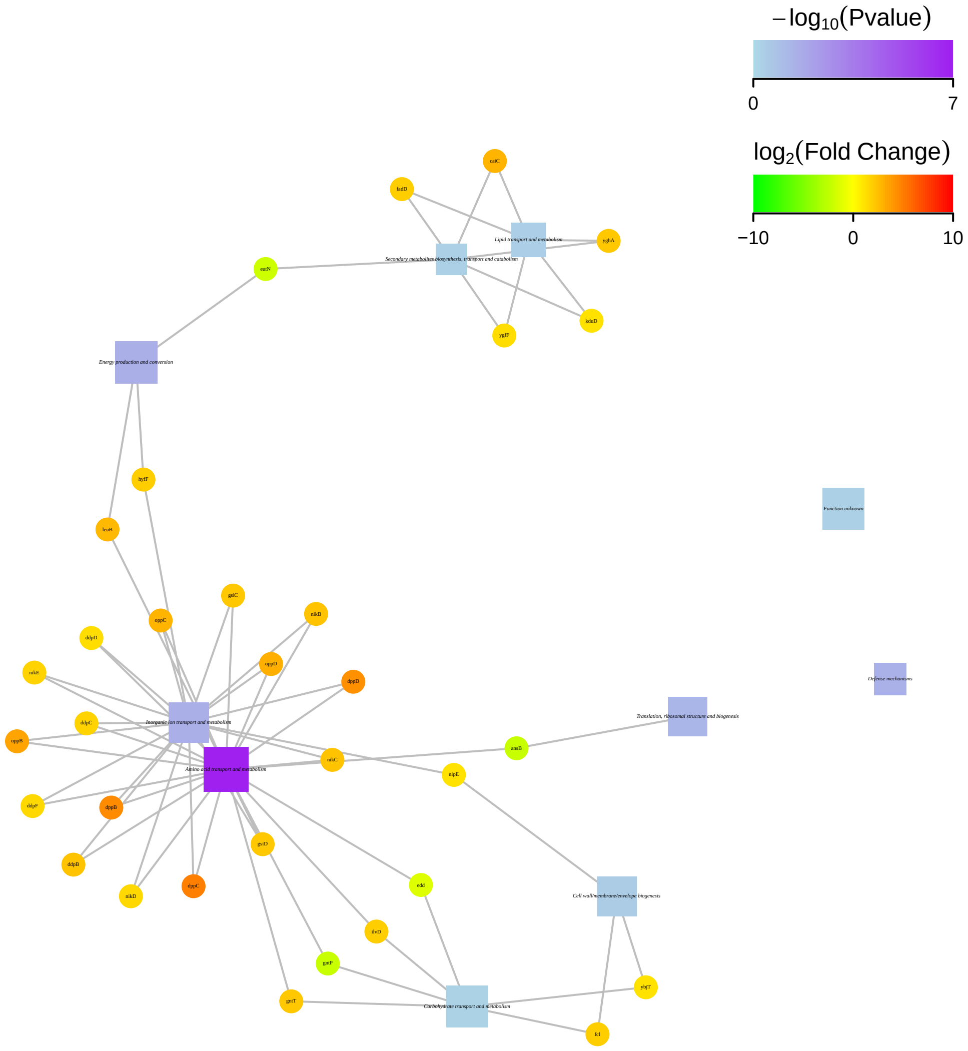

$-\log_{10}(Pvalue)$
0
7
$\log_2(Fold\ Change)$
−10
0
10
caiC
fadD
Lipid transport and metabolism
yghA
Secondary metabolites biosynthesis, transport and catabolism
eutN
kduD
ygfF
Energy production and conversion
hyfF
leuB
Function unknown
gsiC
nikB
oppC
ddpD
oppD
dppD
Defense mechanisms
nikE
ddpC
Inorganic ion transport and metabolism
oppB
Translation, ribosomal structure and biogenesis
nikC
ansB
Amino acid transport and metabolism
nlpE
ddpF
dppB
gsiD
ddpB
nikD
dppC
edd
Cell wall/membrane/envelope biogenesis
ilvD
gntP
ybjT
gntT
Carbohydrate transport and metabolism
fcl

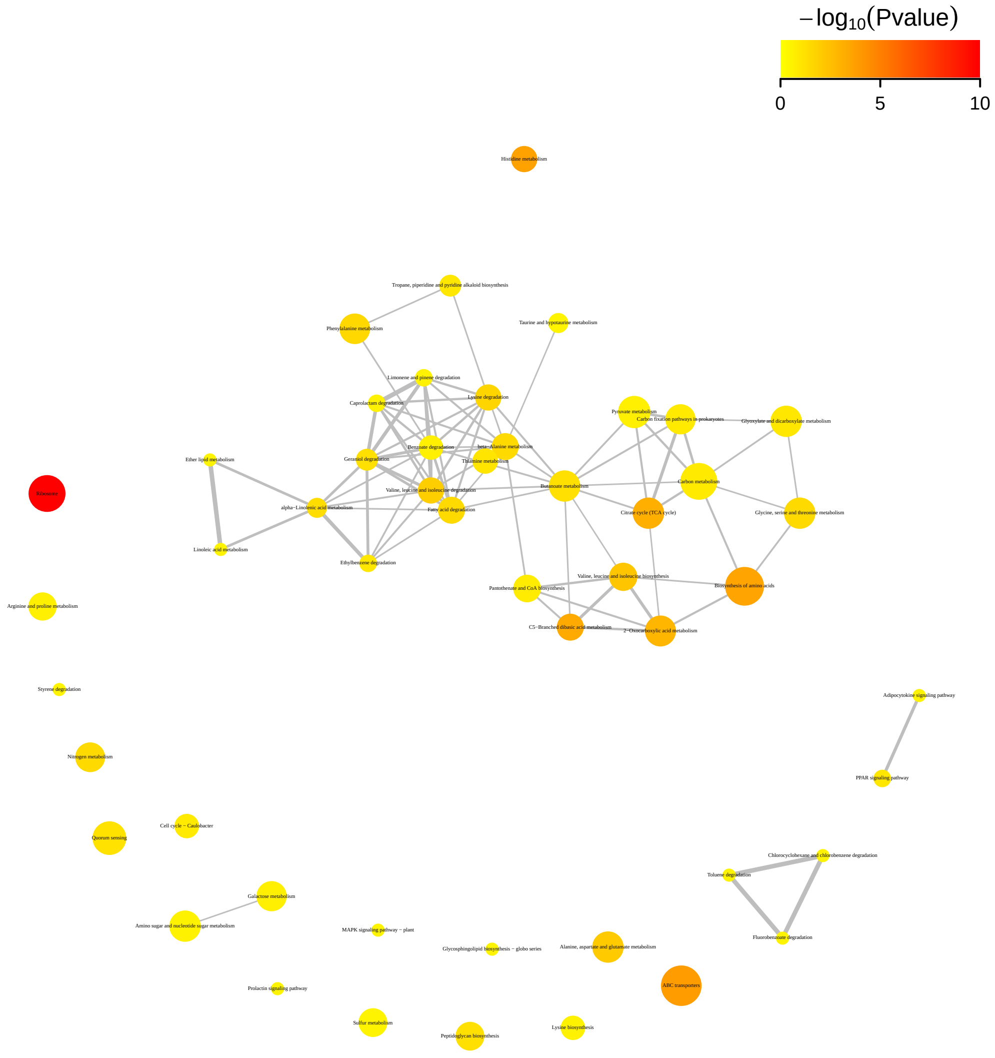

$-\log_{10}(\mathrm{Pvalue})$
0
5
10
Histidine metabolism
Tropane, piperidine and pyridine alkaloid biosynthesis
Phenylalanine metabolism
Taurine and hypotaurine metabolism
Limonene and pinene degradation
Caprolactam degradation
Lysine degradation
Pyruvate metabolism
Carbon fixation pathways in prokaryotes
Glyoxylate and dicarboxylate metabolism
Benzoate degradation
beta−Alanine metabolism
Geraniol degradation
Thiamine metabolism
Ether lipid metabolism
Valine, leucine and isoleucine degradation
Butanoate metabolism
Carbon metabolism
Ribosome
alpha−Linolenic acid metabolism
Fatty acid degradation
Citrate cycle (TCA cycle)
Glycine, serine and threonine metabolism
Linoleic acid metabolism
Ethylbenzene degradation
Valine, leucine and isoleucine biosynthesis
Pantothenate and CoA biosynthesis
Biosynthesis of amino acids
Arginine and proline metabolism
C5−Branched dibasic acid metabolism
2−Oxocarboxylic acid metabolism
Styrene degradation
Adipocytokine signaling pathway
Nitrogen metabolism
PPAR signaling pathway
Cell cycle − Caulobacter
Quorum sensing
Chlorocyclohexane and chlorobenzene degradation
Toluene degradation
Galactose metabolism
Amino sugar and nucleotide sugar metabolism
MAPK signaling pathway − plant
Fluorobenzoate degradation
Glycosphingolipid biosynthesis − globo series
Alanine, aspartate and glutamate metabolism
ABC transporters
Prolactin signaling pathway
Sulfur metabolism
Lysine biosynthesis
Peptidoglycan biosynthesis

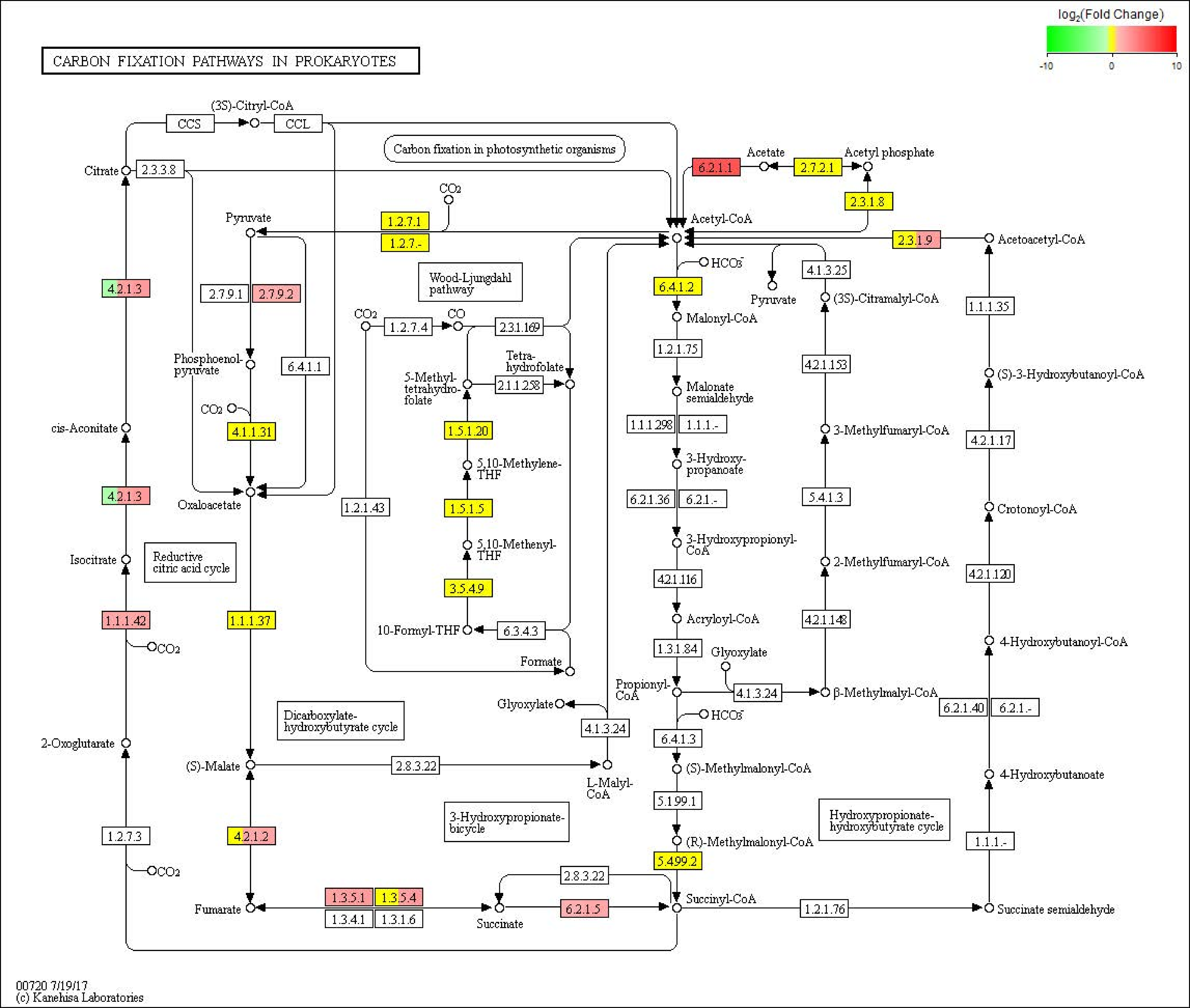

log2(Fold Change)
-10
0
10
CARBON FIXATION PATHWAYS IN PROKARYOTES
(3S)-Citryl-CoA
CCS
CCL
Citrate
2.3.3.8
Carbon fixation in photosynthetic organisms
Acetate
Acetyl phosphate
6.2.1.1
2.7.2.1
2.3.1.8
CO2
Pyruvate
1.2.7.1
1.2.7.-
Acetyl-CoA
2.3.1.9
Acetoacetyl-CoA
HCO3-
4.2.1.3
2.7.9.1
2.7.9.2
Wood-Ljungdahl pathway
6.4.1.2
Pyruvate
4.1.3.25
(3S)-Citramalyl-CoA
Malonyl-CoA
1.1.1.35
CO2
1.2.7.4
CO
2.3.1.169
1.2.1.75
4.2.1.153
Phosphoenol-pyruvate
6.4.1.1
Tetra-hydrofolate
5-Methyl-tetrahydro-folate
2.1.1.258
Malonate semialdehyde
(S)-3-Hydroxybutanoyl-CoA
CO2
4.1.1.31
1.5.1.20
1.1.1.298
1.1.1.-
3-Methylfumaryl-CoA
cis-Aconitate
4.2.1.17
5,10-Methylene-THF
3-Hydroxy-propanoate
4.2.1.3
Oxaloacetate
1.2.1.43
1.5.1.5
6.2.1.36
6.2.1.-
5.4.1.3
Crotonoyl-CoA
5,10-Methenyl-THF
3-Hydroxypropionyl-CoA
Isocitrate
Reductive citric acid cycle
2-Methylfumaryl-CoA
3.5.4.9
4.2.1.116
4.2.1.120
1.1.1.42
1.1.1.37
10-Formyl-THF
6.3.4.3
Acryloyl-CoA
4.2.1.148
CO2
1.3.1.84
Glyoxylate
4-Hydroxybutanoyl-CoA
Formate
Propionyl-CoA
4.1.3.24
β-Methylmalyl-CoA
Dicarboxylate-hydroxybutyrate cycle
Glyoxylate
6.2.1.40
6.2.1.-
4.1.3.24
HCO3-
6.4.1.3
2-Oxoglutarate
(S)-Malate
2.8.3.22
L-Malyl-CoA
(S)-Methylmalonyl-CoA
4-Hydroxybutanoate
5.1.99.1
3-Hydroxypropionate-bicycle
Hydroxypropionate-hydroxybutyrate cycle
1.2.7.3
4.2.1.2
(R)-Methylmalonyl-CoA
1.1.1.-
CO2
5.4.99.2
2.8.3.22
1.3.5.1
1.3.5.4
Succinyl-CoA
Fumarate
6.2.1.5
1.2.1.76
Succinate semialdehyde
1.3.4.1
1.3.1.6
Succinate
00720 7/19/17
(c) Kanehisa Laboratories

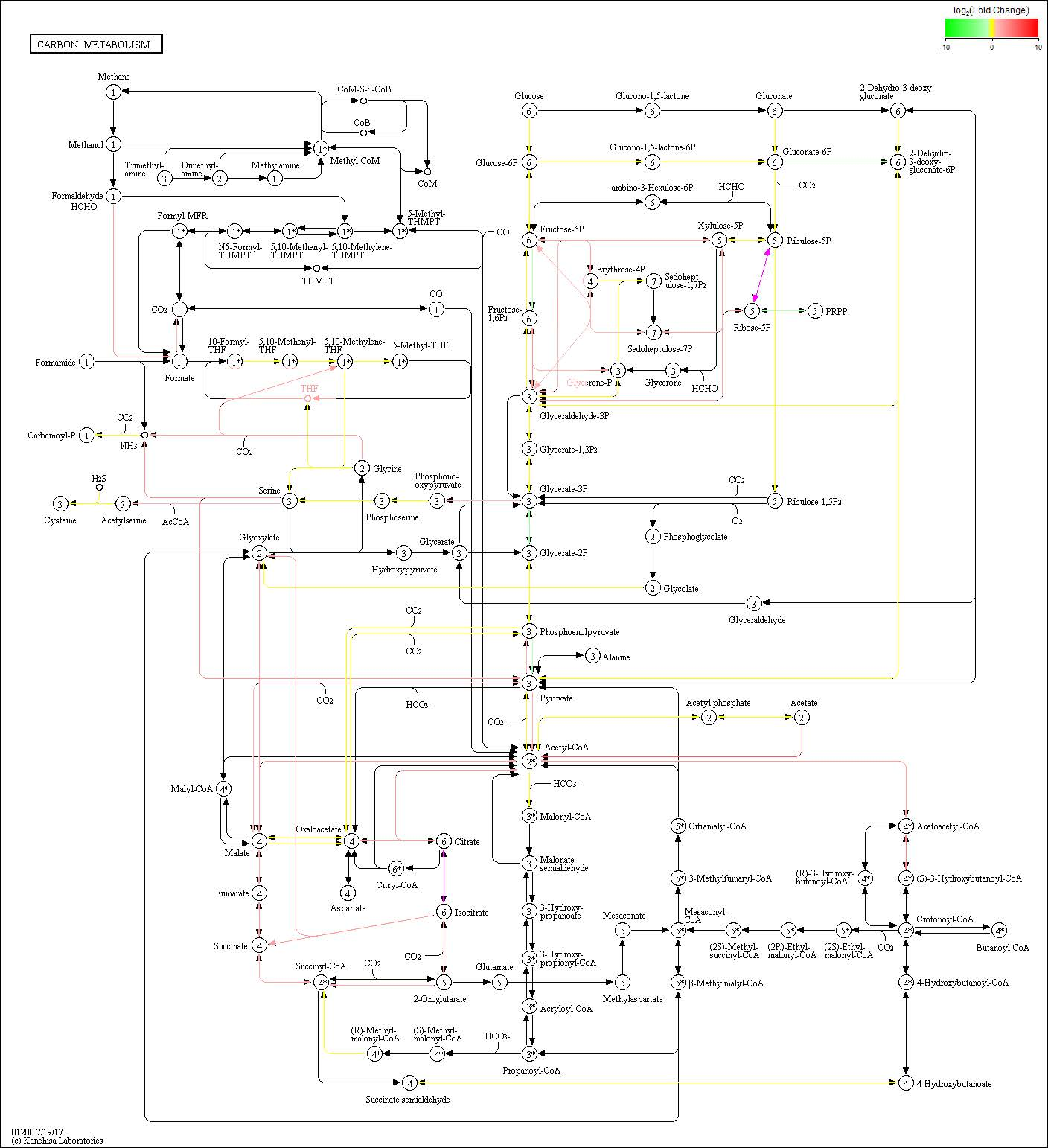
CARBON METABOLISM
log2(Fold Change)
-10
0
10
Methane
Methanol
Formaldehyde
HCHO
Trimethyl-
amine
Dimethyl-
amine
Methylamine
CoM-S-S-CoB
CoB
Methyl-CoM
CoM
Formyl-MFR
N5-Formyl-
THMPT
5,10-Methenyl-
THMPT
5,10-Methylene-
THMPT
5-Methyl-
THMPT
CO
THMPT
CO2
Formamide
Formate
10-Formyl-
THF
5,10-Methenyl-
THF
5,10-Methylene-
THF
5-Methyl-THF
THF
Carbamoyl-P
NH3
H2S
Cysteine
Acetylserine
AcCoA
Serine
Glycine
Phosphoserine
Phosphono-
oxypyruvate
Glyoxylate
Hydroxypyruvate
Glycerate
Glucose
Glucono-1,5-lactone
Gluconate
2-Dehydro-3-deoxy-
gluconate
Glucose-6P
Glucono-1,5-lactone-6P
Gluconate-6P
2-Dehydro-
3-deoxy-
gluconate-6P
arabino-3-Hexulose-6P
HCHO
Fructose-6P
Xylulose-5P
Ribulose-5P
Erythrose-4P
Sedohept-
ulose-1,7P2
Fructose-
1,6P2
Ribose-5P
PRPP
Sedoheptulose-7P
Glycerone-P
Glycerone
Glyceraldehyde-3P
Glycerate-1,3P2
Glycerate-3P
Ribulose-1,5P2
O2
Phosphoglycolate
Glycerate-2P
Glycolate
Glyceraldehyde
Phosphoenolpyruvate
Alanine
Pyruvate
HCO3-
Acetyl phosphate
Acetate
Acetyl-CoA
Malyl-CoA
Malonyl-CoA
Malate
Oxaloacetate
Citrate
Citryl-CoA
Aspartate
Fumarate
Isocitrate
Succinate
Malonate
semialdehyde
3-Hydroxy-
propanoate
3-Hydroxy-
propionyl-CoA
Acryloyl-CoA
Propanoyl-CoA
Citramalyl-CoA
3-Methylfumaryl-CoA
Mesaconate
Mesaconyl-
CoA
(2S)-Methyl-
succinyl-CoA
(2R)-Ethyl-
malonyl-CoA
(2S)-Ethyl-
malonyl-CoA
Crotonoyl-CoA
Butanoyl-CoA
Acetoacetyl-CoA
(R)-3-Hydroxy-
butanoyl-CoA
(S)-3-Hydroxybutanoyl-CoA
4-Hydroxybutanoyl-CoA
4-Hydroxybutanoate
β-Methylmalyl-CoA
Methylaspartate
Glutamate
2-Oxoglutarate
Succinyl-CoA
(R)-Methyl-
malonyl-CoA
(S)-Methyl-
malonyl-CoA
Succinate semialdehyde
01200 7/19/17
(c) Kanehisa Laboratories

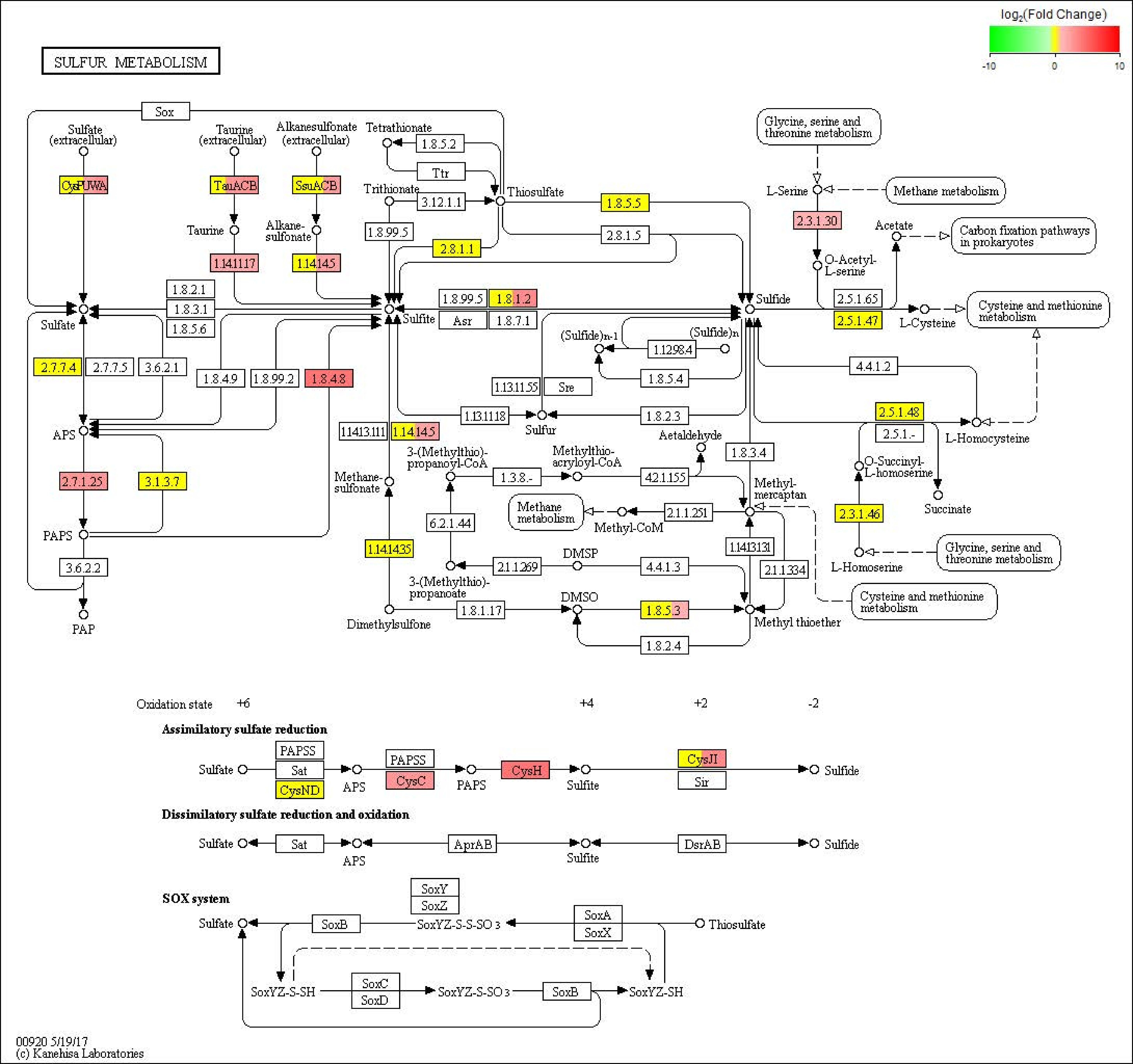
log2(Fold Change)
-10
0
10
SULFUR METABOLISM
Sox
Sulfate (extracellular)
Taurine (extracellular)
Alkanesulfonate (extracellular)
Tetrathionate
1.8.5.2
Ttr
Trithionate
3.12.1.1
Thiosulfate
1.8.5.5
2.8.1.5
1.8.99.5
2.8.1.1
CysPUWA
TauACB
SsuACB
Taurine
Alkane-sulfonate
1.14.11.17
1.14.14.5
1.8.2.1
1.8.3.1
1.8.5.6
Sulfate
Sulfite
1.8.99.5
1.8.1.2
Asr
1.8.7.1
Sulfide
2.7.7.4
2.7.7.5
3.6.2.1
1.8.4.9
1.8.99.2
1.8.4.8
(Sulfide)n-1
(Sulfide)n
1.12.98.4
1.8.5.4
1.13.11.55
Sre
1.13.11.18
Sulfur
1.8.2.3
APS
1.14.13.111
1.14.14.5
2.7.1.25
3.1.3.7
PAPS
3.6.2.2
PAP
Methane-sulfonate
1.14.14.35
3-(Methylthio)-propanoyl-CoA
1.3.8.-
Methylthio-acryloyl-CoA
4.2.1.155
Acetaldehyde
1.8.3.4
Methyl-mercaptan
6.2.1.44
Methane metabolism
Methyl-CoM
2.1.1.251
1.14.13.131
2.1.1.269
DMSP
4.4.1.3
2.1.1.334
3-(Methylthio)-propanoate
Dimethylsulfone
1.8.1.17
DMSO
1.8.5.3
Methyl thioether
1.8.2.4
Glycine, serine and threonine metabolism
L-Serine
Methane metabolism
2.3.1.30
Acetate
Carbon fixation pathways in prokaryotes
O-Acetyl-L-serine
2.5.1.65
2.5.1.47
L-Cysteine
Cysteine and methionine metabolism
4.4.1.2
2.5.1.48
2.5.1.-
L-Homocysteine
O-Succinyl-L-homoserine
Succinate
2.3.1.46
L-Homoserine
Glycine, serine and threonine metabolism
Cysteine and methionine metabolism
Oxidation state
+6
+4
+2
-2
Assimilatory sulfate reduction
Sulfate
PAPSS
Sat
CysND
APS
PAPSS
CysC
PAPS
CysH
Sulfite
CysJI
Sir
Sulfide
Dissimilatory sulfate reduction and oxidation
Sulfate
Sat
APS
AprAB
Sulfite
DsrAB
Sulfide
SOX system
Sulfate
SoxB
SoxY
SoxZ
SoxYZ-S-S-SO3
SoxA
SoxX
Thiosulfate
SoxYZ-S-SH
SoxC
SoxD
SoxYZ-S-SO3
SoxB
SoxYZ-SH
00920 5/19/17
(c) Kanehisa Laboratories

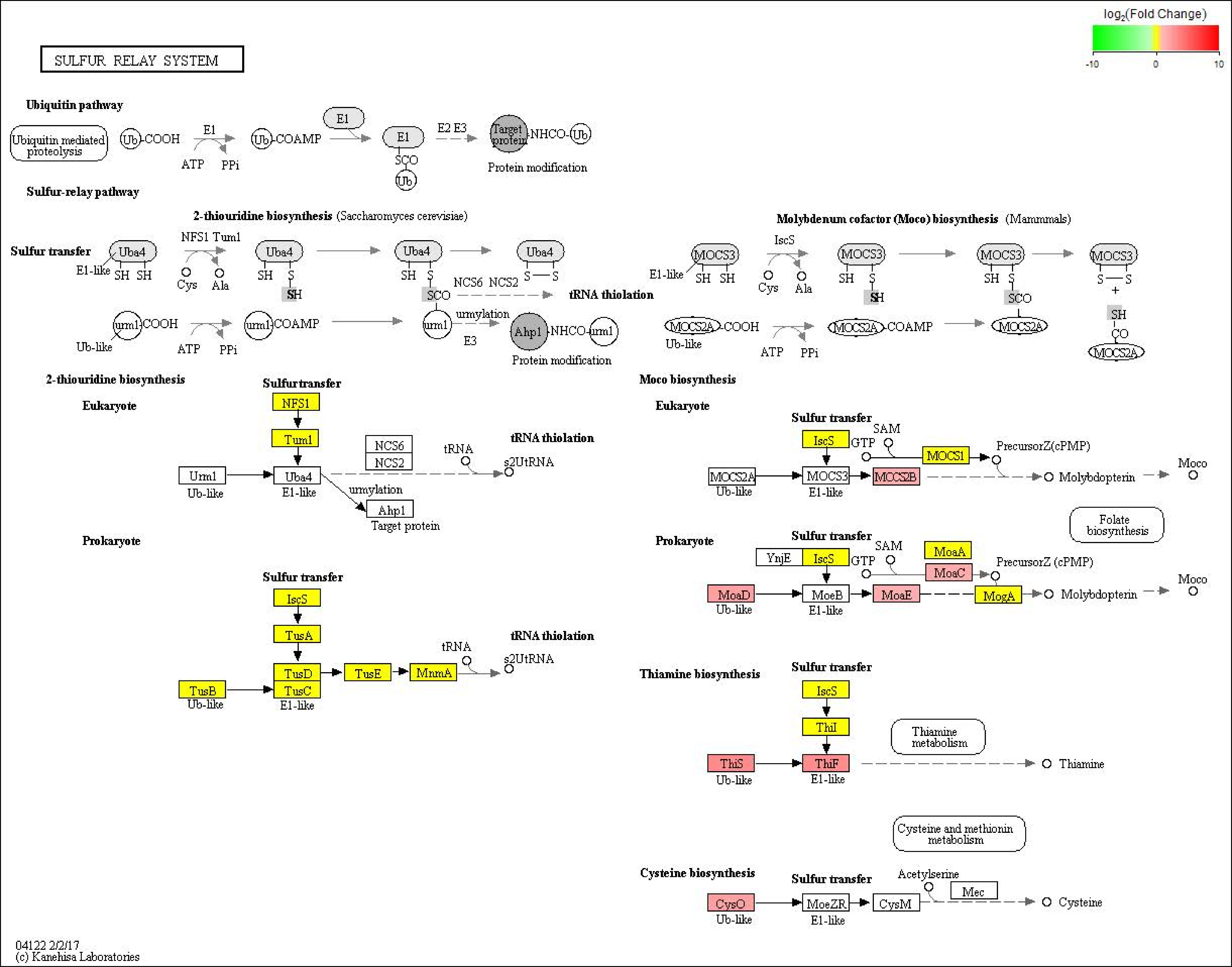
log2(Fold Change)
-10
0
10
SULFUR RELAY SYSTEM
Ubiquitin pathway
Ubiquitin mediated proteolysis
Ub-COOH
E1
ATP
PPi
Ub-COAMP
E1
E1
SCO
Ub
E2 E3
Target protein-NHCO-Ub
Protein modification
Sulfur-relay pathway
2-thiouridine biosynthesis (Saccharomyces cerevisiae)
Sulfur transfer
Uba4
E1-like
SH SH
NFS1 Tum1
Cys
Ala
NCS6 NCS2
tRNA thiolation
urm1-COOH
Ub-like
urm1-COAMP
urmylation
E3
Ahp1-NHCO-urm1
Molybdenum cofactor (Moco) biosynthesis (Mammals)
MOCS3
IscS
MOCS2A-COOH
MOCS2A-COAMP
2-thiouridine biosynthesis
Eukaryote
Sulfur transfer
NFS1
Tum1
NCS6
NCS2
tRNA
s2UtRNA
Urm1
Uba4
E1-like
Ahp1
Target protein
Prokaryote
IscS
TusA
TusD
TusC
TusE
MnmA
TusB
Moco biosynthesis
SAM
GTP
MOCS1
PrecursorZ(cPMP)
MOCS2A
MOCS3
MOCS2B
Molybdopterin
Moco
Folate biosynthesis
YnjE
MoaA
MoaC
PrecursorZ (cPMP)
MoaD
MoeB
MoaE
MogA
Thiamine biosynthesis
ThiI
Thiamine metabolism
ThiS
ThiF
Thiamine
Cysteine and methionin metabolism
Cysteine biosynthesis
Acetylserine
Mec
CysO
MoeZR
CysM
Cysteine
04122 2/2/17
(c) Kanehisa Laboratories

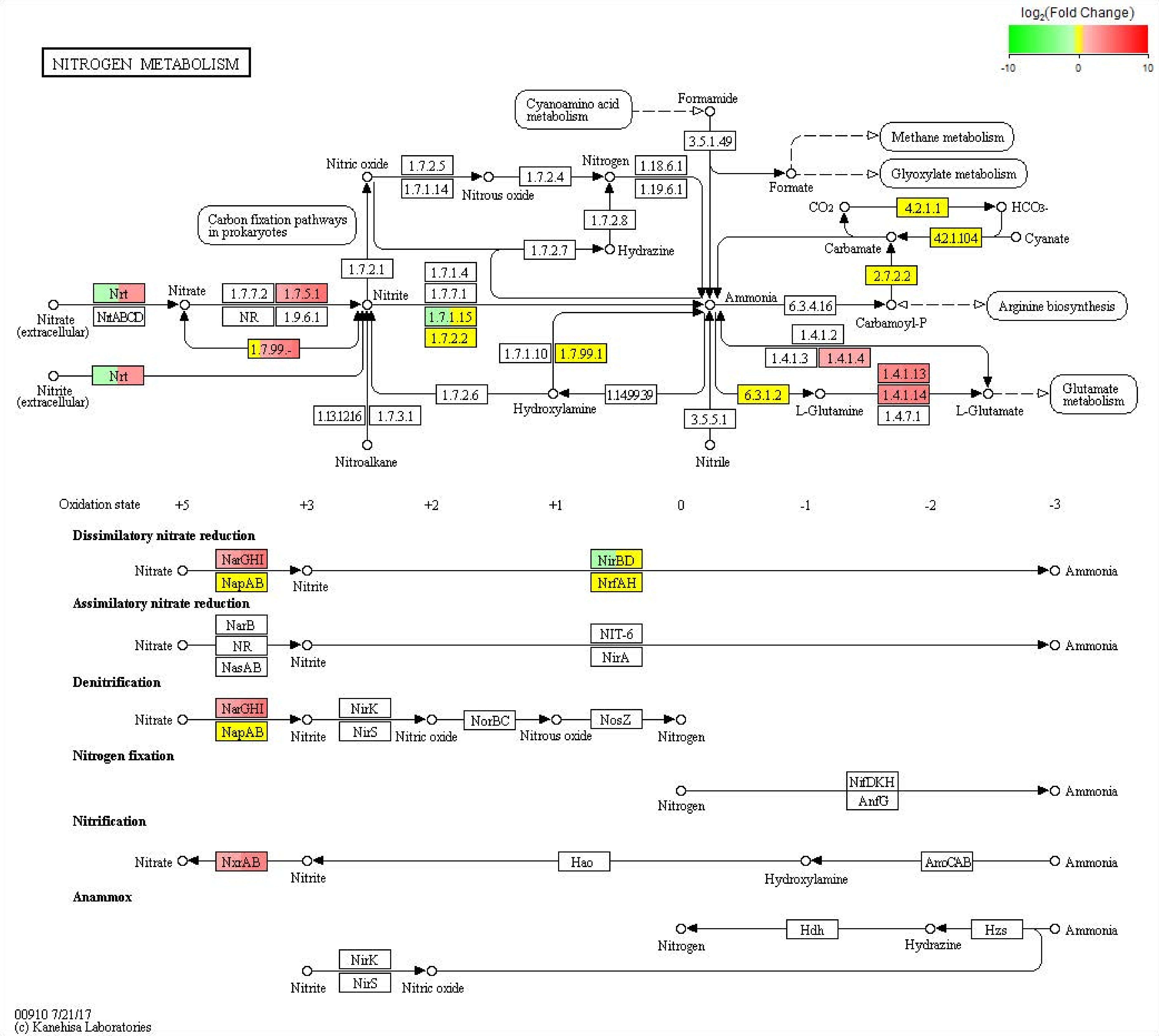

NITROGEN METABOLISM
log2(Fold Change)
-10
0
10
Cyanoamino acid metabolism
Formamide
3.5.1.49
Methane metabolism
Glyoxylate metabolism
Formate
Nitric oxide
1.7.2.5
1.7.1.14
Nitrous oxide
1.7.2.4
Nitrogen
1.18.6.1
1.19.6.1
CO2
4.2.1.1
HCO3-
Carbon fixation pathways in prokaryotes
1.7.2.8
1.7.2.7
Hydrazine
Carbamate
4.2.1.104
Cyanate
1.7.2.1
1.7.1.4
2.7.2.2
Nrt
Nitrate
1.7.7.2
1.7.5.1
Nitrite
1.7.7.1
Ammonia
6.3.4.16
Carbamoyl-P
Arginine biosynthesis
Nitrate (extracellular)
NrtABCD
NR
1.9.6.1
1.7.1.15
1.7.2.2
1.7.99.-
1.4.1.2
1.7.1.10
1.7.99.1
1.4.1.3
1.4.1.4
1.4.1.13
Nitrite (extracellular)
1.7.2.6
Hydroxylamine
1.14.99.39
6.3.1.2
L-Glutamine
1.4.1.14
L-Glutamate
Glutamate metabolism
1.13.12.16
1.7.3.1
3.5.5.1
1.4.7.1
Nitroalkane
Nitrile
Oxidation state +5 +3 +2 +1 0 -1 -2 -3
Dissimilatory nitrate reduction
Nitrate
NarGHI
NapAB
Nitrite
NirBD
NrfAH
Ammonia
Assimilatory nitrate reduction
Nitrate
NarB
NR
NasAB
Nitrite
NIT-6
NirA
Ammonia
Denitrification
Nitrate
NarGHI
NapAB
Nitrite
NirK
NirS
Nitric oxide
NorBC
Nitrous oxide
NosZ
Nitrogen
Nitrogen fixation
Nitrogen
NifDKH
AnfG
Ammonia
Nitrification
Nitrate
NxrAB
Nitrite
Hao
Hydroxylamine
AmoCAB
Ammonia
Anammox
Nitrogen
Hdh
Hydrazine
Hzs
Ammonia
Nitrite
NirK
NirS
Nitric oxide
00910 7/21/17
(c) Kanehisa Laboratories

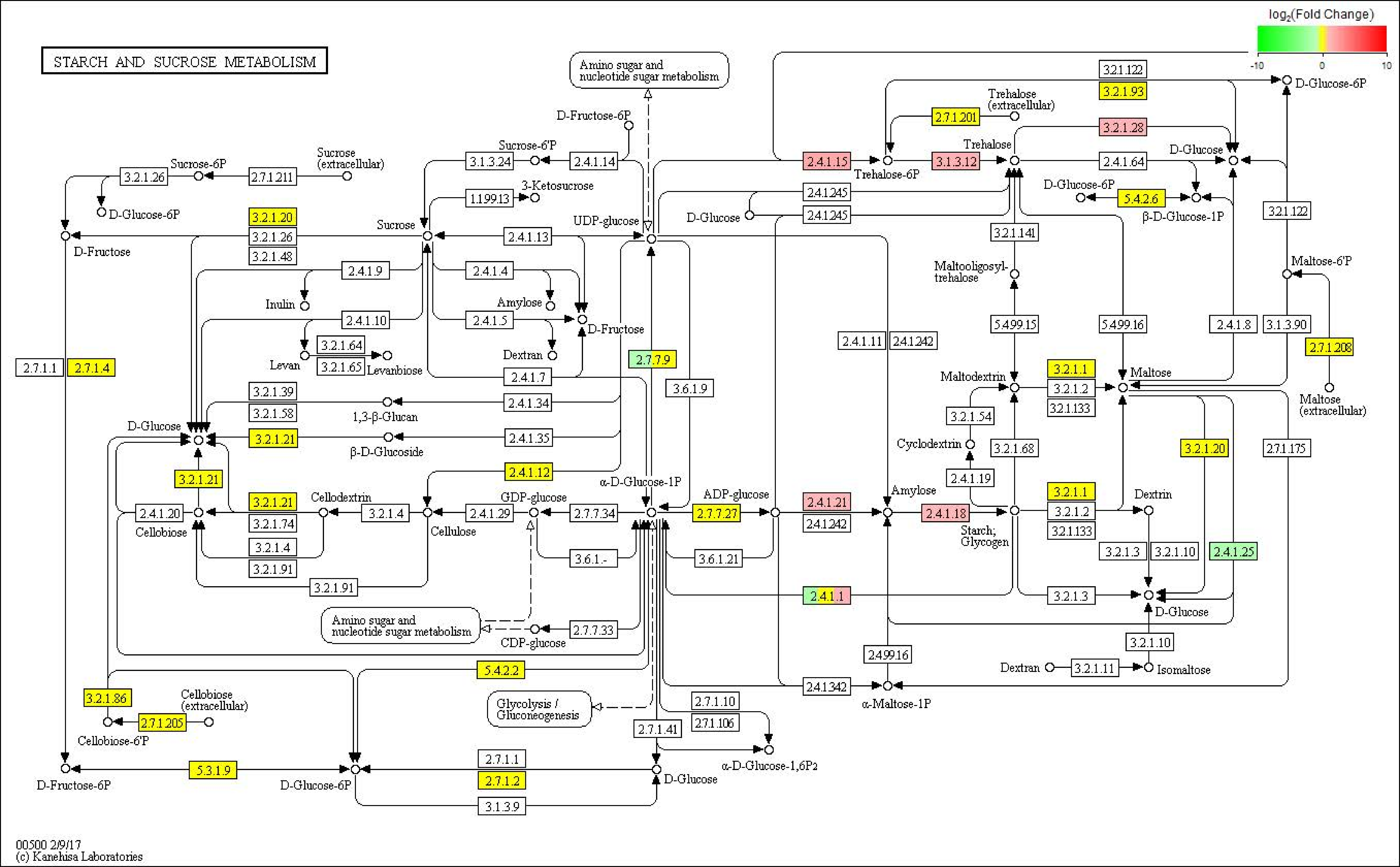
STARCH AND SUCROSE METABOLISM
log2(Fold Change)
-10
0
10
Amino sugar and nucleotide sugar metabolism
D-Fructose-6P
Sucrose-6'P
3.1.3.24
2.4.1.14
Sucrose (extracellular)
Sucrose-6P
3.2.1.26
2.7.1.211
D-Glucose-6P
3-Ketosucrose
1.1.99.13
Sucrose
UDP-glucose
3.2.1.20
3.2.1.26
3.2.1.48
D-Fructose
2.4.1.13
2.4.1.9
2.4.1.4
Inulin
Amylose
2.4.1.10
2.4.1.5
D-Fructose
Levan
3.2.1.64
3.2.1.65
Levanbiose
Dextran
2.4.1.7
2.7.7.9
3.6.1.9
2.7.1.1
2.7.1.4
3.2.1.39
3.2.1.58
1,3-β-Glucan
2.4.1.34
D-Glucose
3.2.1.21
β-D-Glucoside
2.4.1.35
2.4.1.12
α-D-Glucose-1P
3.2.1.21
3.2.1.21
Cellodextrin
GDP-glucose
2.4.1.20
Cellobiose
3.2.1.74
3.2.1.4
Cellulose
2.4.1.29
2.7.7.34
3.2.1.4
3.2.1.91
3.6.1.-
3.2.1.91
Amino sugar and nucleotide sugar metabolism
CDP-glucose
2.7.7.33
5.4.2.2
3.2.1.86
Cellobiose (extracellular)
2.7.1.205
Cellobiose-6'P
Glycolysis / Gluconeogenesis
2.7.1.10
2.7.1.106
2.7.1.41
α-D-Glucose-1,6P2
D-Fructose-6P
5.3.1.9
D-Glucose-6P
2.7.1.1
2.7.1.2
D-Glucose
3.1.3.9
3.2.1.122
3.2.1.93
D-Glucose-6P
Trehalose (extracellular)
2.7.1.201
3.2.1.28
Trehalose
D-Glucose
2.4.1.15
Trehalose-6P
3.1.3.12
2.4.1.64
2.4.1.245
D-Glucose-6P
5.4.2.6
D-Glucose
2.4.1.245
β-D-Glucose-1P
3.2.1.122
3.2.1.141
Maltooligosyl-trehalose
Maltose-6'P
5.4.99.15
5.4.99.16
2.4.1.8
3.1.3.90
2.4.1.11
2.4.1.242
2.7.1.208
Maltodextrin
3.2.1.1
3.2.1.2
3.2.1.133
Maltose
Maltose (extracellular)
3.2.1.54
Cyclodextrin
3.2.1.68
3.2.1.20
2.7.1.175
2.4.1.19
ADP-glucose
Amylose
3.2.1.1
Dextrin
2.7.7.27
2.4.1.21
2.4.1.242
2.4.1.18
Starch; Glycogen
3.2.1.2
3.2.1.133
3.6.1.21
3.2.1.3
3.2.1.10
2.4.1.25
2.4.1.1
3.2.1.3
D-Glucose
3.2.1.10
2.4.99.16
Dextran
3.2.1.11
Isomaltose
2.4.1.342
α-Maltose-1P
00500 2/9/17
(c) Kanehisa Laboratories

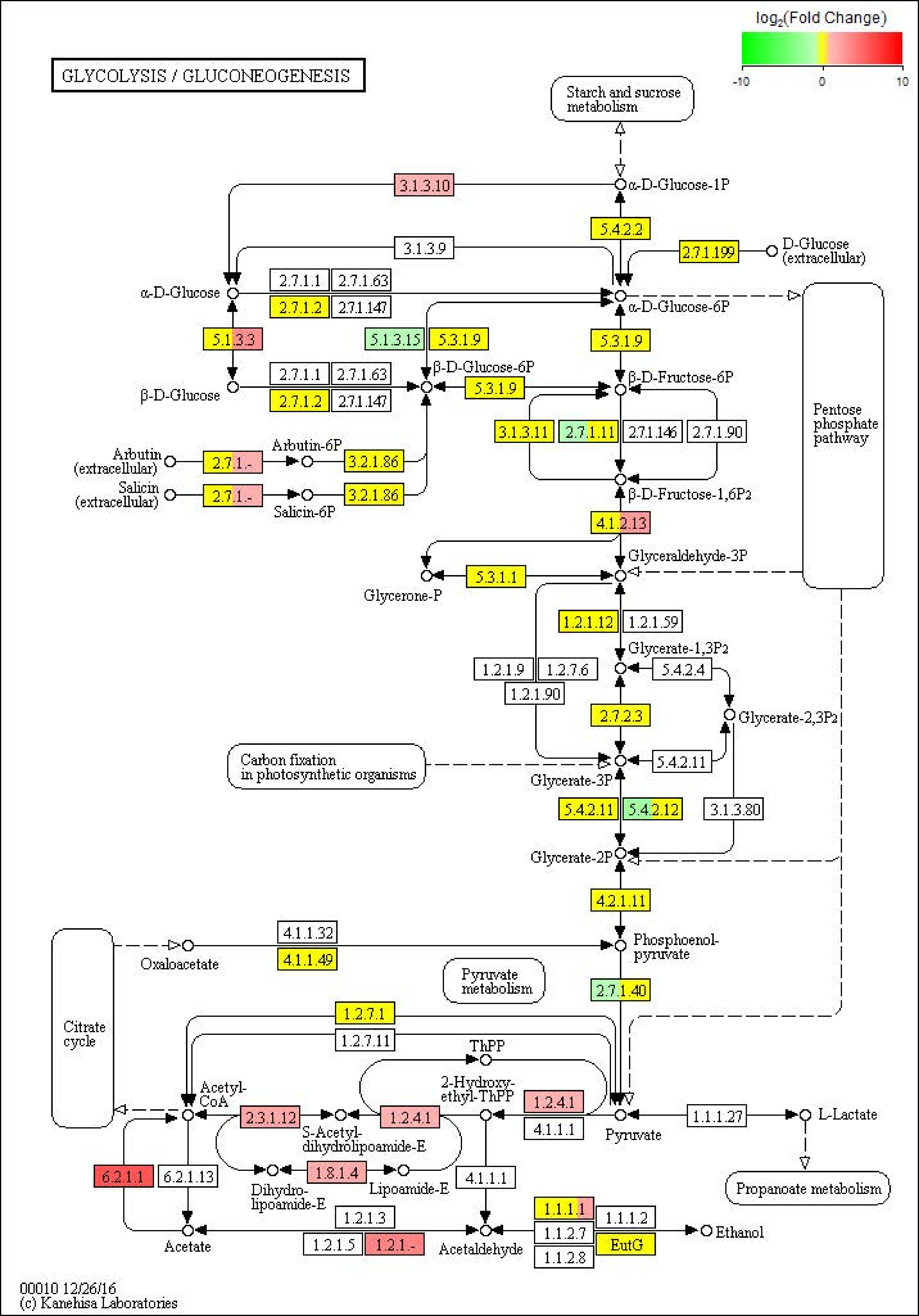
GLYCOLYSIS / GLUCONEOGENESIS
log2(Fold Change)
-10
0
10
Starch and sucrose metabolism
α-D-Glucose-1P
3.1.3.10
5.4.2.2
3.1.3.9
2.7.1.199
D-Glucose (extracellular)
α-D-Glucose
2.7.1.1
2.7.1.63
2.7.1.2
2.7.1.147
α-D-Glucose-6P
5.1.3.3
5.1.3.15
5.3.1.9
β-D-Glucose-6P
β-D-Fructose-6P
β-D-Glucose
Pentose phosphate pathway
3.1.3.11
2.7.1.11
2.7.1.146
2.7.1.90
Arbutin (extracellular)
2.7.1.-
Arbutin-6P
3.2.1.86
Salicin (extracellular)
Salicin-6P
β-D-Fructose-1,6P2
4.1.2.13
Glyceraldehyde-3P
5.3.1.1
Glycerone-P
1.2.1.12
1.2.1.59
Glycerate-1,3P2
1.2.1.9
1.2.7.6
5.4.2.4
1.2.1.90
2.7.2.3
Glycerate-2,3P2
Carbon fixation in photosynthetic organisms
5.4.2.11
Glycerate-3P
5.4.2.12
3.1.3.80
Glycerate-2P
4.2.1.11
Citrate cycle
4.1.1.32
4.1.1.49
Oxaloacetate
Phosphoenol-pyruvate
Pyruvate metabolism
2.7.1.40
1.2.7.1
1.2.7.11
ThPP
Acetyl-CoA
2-Hydroxy-ethyl-ThPP
1.2.4.1
2.3.1.12
S-Acetyl-dihydrolipoamide-E
4.1.1.1
Pyruvate
1.1.1.27
L-Lactate
6.2.1.1
6.2.1.13
Dihydro-lipoamide-E
1.8.1.4
Lipoamide-E
Propanoate metabolism
1.1.1.1
1.1.1.2
1.2.1.3
1.1.2.7
Ethanol
Acetate
1.2.1.5
1.2.1.-
Acetaldehyde
EutG
1.1.2.8
00010 12/26/16
(c) Kanehisa Laboratories

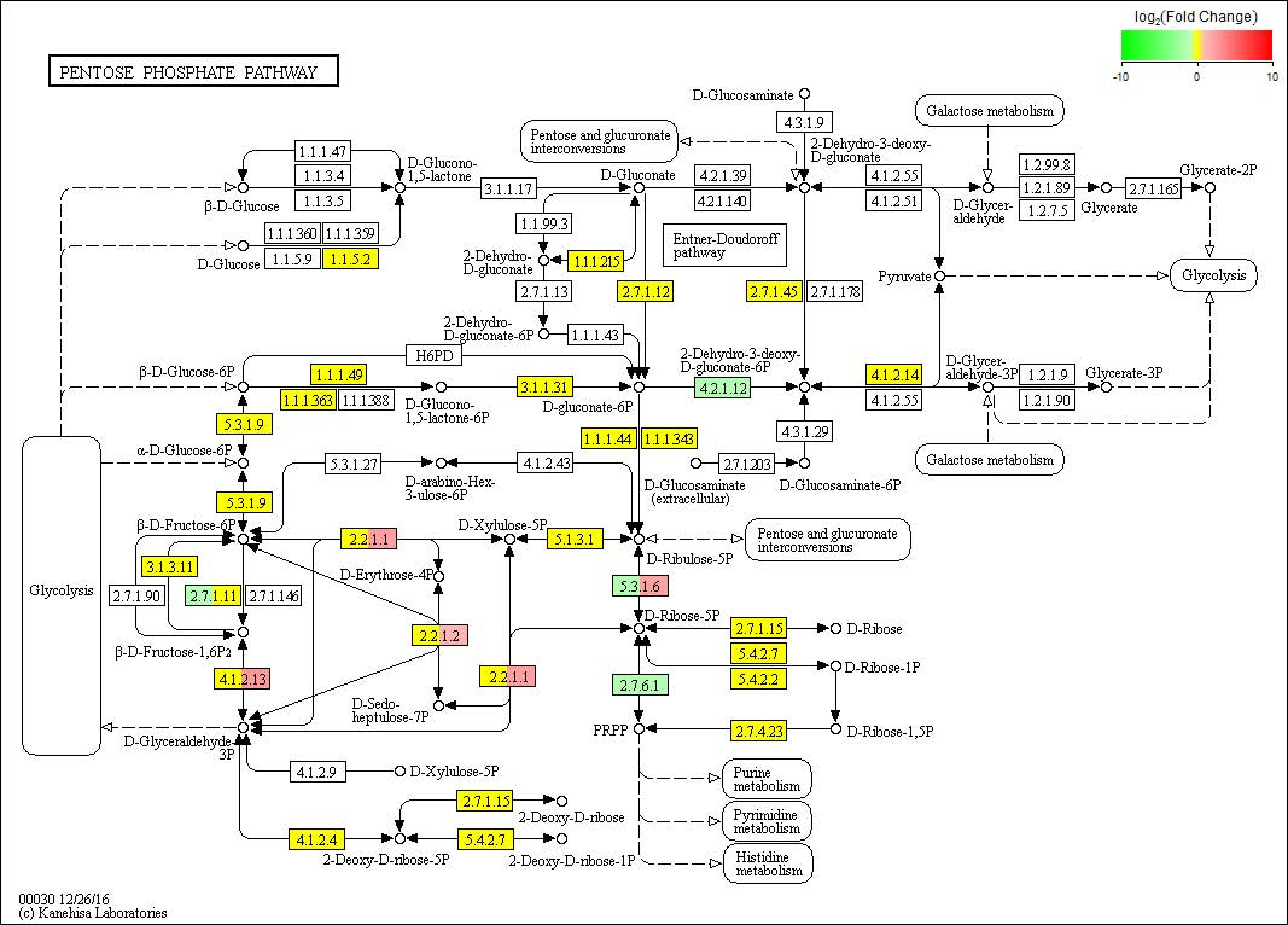

PENTOSE PHOSPHATE PATHWAY
log2(Fold Change)
-10
0
10
D-Glucosaminate
4.3.1.9
Galactose metabolism
Pentose and glucuronate interconversions
2-Dehydro-3-deoxy-D-gluconate
1.1.1.47
1.1.3.4
1.1.3.5
D-Glucono-1,5-lactone
β-D-Glucose
3.1.1.17
D-Gluconate
4.2.1.39
4.2.1.140
4.1.2.55
4.1.2.51
1.2.99.8
1.2.1.89
1.2.7.5
D-Glyceraldehyde
Glycerate
2.7.1.165
Glycerate-2P
1.1.99.3
1.1.1.360
1.1.1.359
D-Glucose
1.1.5.9
1.1.5.2
2-Dehydro-D-gluconate
1.1.1.215
Entner-Doudoroff pathway
2.7.1.13
2.7.1.12
2.7.1.45
2.7.1.178
Pyruvate
Glycolysis
2-Dehydro-D-gluconate-6P
1.1.1.43
H6PD
2-Dehydro-3-deoxy-D-gluconate-6P
β-D-Glucose-6P
1.1.1.49
1.1.1.363
1.1.1.388
D-Glucono-1,5-lactone-6P
3.1.1.31
D-gluconate-6P
4.2.1.12
4.1.2.14
4.1.2.55
D-Glyceraldehyde-3P
1.2.1.9
1.2.1.90
Glycerate-3P
5.3.1.9
α-D-Glucose-6P
1.1.1.44
1.1.1.343
4.3.1.29
5.3.1.27
D-arabino-Hex-3-ulose-6P
4.1.2.43
2.7.1.203
D-Glucosamine (extracellular)
D-Glucosamine-6P
Galactose metabolism
5.3.1.9
β-D-Fructose-6P
2.2.1.1
D-Xylulose-5P
5.1.3.1
D-Ribulose-5P
Pentose and glucuronate interconversions
3.1.3.11
D-Erythrose-4P
2.7.1.90
2.7.1.11
2.7.1.146
5.3.1.6
D-Ribose-5P
2.7.1.15
D-Ribose
β-D-Fructose-1,6P2
2.2.1.2
5.4.2.7
D-Ribose-1P
5.4.2.2
4.1.2.13
2.2.1.1
2.7.6.1
D-Sedoheptulose-7P
PRPP
2.7.4.23
D-Ribose-1,5P
D-Glyceraldehyde-3P
4.1.2.9
D-Xylulose-5P
Purine metabolism
2.7.1.15
2-Deoxy-D-ribose
Pyrimidine metabolism
4.1.2.4
5.4.2.7
2-Deoxy-D-ribose-5P
2-Deoxy-D-ribose-1P
Histidine metabolism
00030 12/26/16
(c) Kanehisa Laboratories

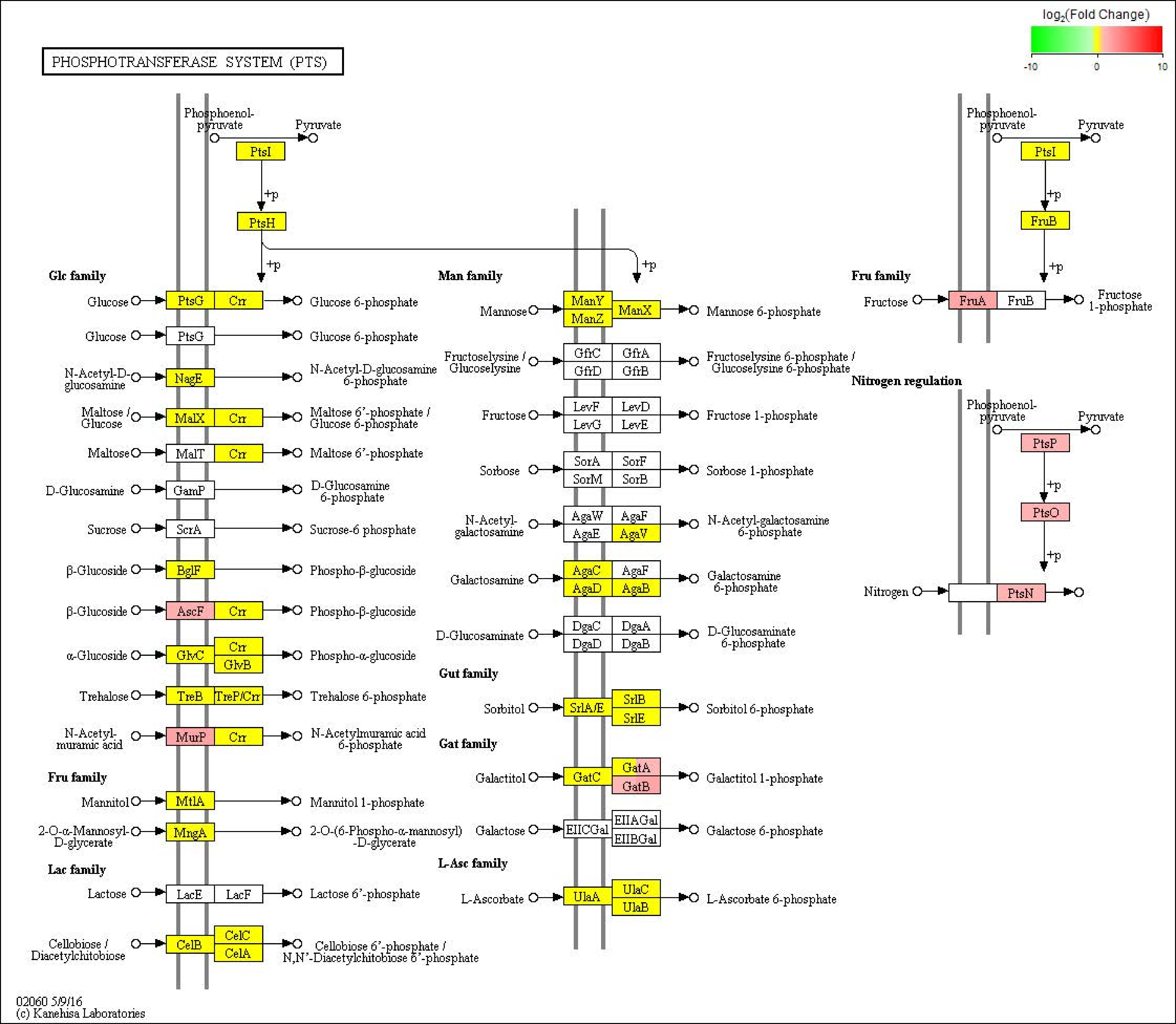
log2(Fold Change)
-10
0
10
PHOSPHOTRANSFERASE SYSTEM (PTS)
Phosphoenol-pyruvate
Pyruvate
PtsI
+p
PtsH
+p
Glc family
Glucose
PtsG
Crr
Glucose 6-phosphate
Glucose
PtsG
Glucose 6-phosphate
N-Acetyl-D-glucosamine
NagE
N-Acetyl-D-glucosamine 6-phosphate
Maltose / Glucose
MalX
Crr
Maltose 6'-phosphate / Glucose 6-phosphate
Maltose
MalT
Crr
Maltose 6'-phosphate
D-Glucosamine
GamP
D-Glucosamine 6-phosphate
Sucrose
ScrA
Sucrose-6 phosphate
β-Glucoside
BglF
Phospho-β-glucoside
β-Glucoside
AscF
Crr
Phospho-β-glucoside
α-Glucoside
GlvC
Crr
GlvB
Phospho-α-glucoside
Trehalose
TreB
TreP/Crr
Trehalose 6-phosphate
N-Acetyl-muramic acid
MurP
Crr
N-Acetylmuramic acid 6-phosphate
Fru family
Mannitol
MtlA
Mannitol 1-phosphate
2-O-α-Mannosyl-D-glycerate
MngA
2-O-(6-Phospho-α-mannosyl)-D-glycerate
Lac family
Lactose
LacE
LacF
Lactose 6'-phosphate
Cellobiose / Diacetylchitobiose
CelB
CelC
CelA
Cellobiose 6'-phosphate / N,N'-Diacetylchitobiose 6'-phosphate
Man family
Mannose
ManY
ManZ
ManX
Mannose 6-phosphate
Fructoselysine / Glucoselysine
GfrC
GfrA
GfrD
GfrB
Fructoselysine 6-phosphate / Glucoselysine 6-phosphate
Fructose
LevF
LevD
LevG
LevE
Fructose 1-phosphate
Sorbose
SorA
SorF
SorM
SorB
Sorbose 1-phosphate
N-Acetyl-galactosamine
AgaW
AgaF
AgaE
AgaV
N-Acetyl-galactosamine 6-phosphate
Galactosamine
AgaC
AgaF
AgaD
AgaB
Galactosamine 6-phosphate
D-Glucosaminate
DgaC
DgaA
DgaD
DgaB
D-Glucosaminate 6-phosphate
Gut family
Sorbitol
SrlA/E
SrlB
SrlE
Sorbitol 6-phosphate
Gat family
Galactitol
GatC
GatA
GatB
Galactitol 1-phosphate
Galactose
EIICGal
EIIAGal
EIIBGal
Galactose 6-phosphate
L-Asc family
L-Ascorbate
UlaA
UlaC
UlaB
L-Ascorbate 6-phosphate
Phosphoenol-pyruvate
Pyruvate
PtsI
+p
FruB
+p
Fru family
Fructose
FruA
FruB
Fructose 1-phosphate
Nitrogen regulation
Phosphoenol-pyruvate
Pyruvate
PtsP
+p
PtsO
+p
Nitrogen
PtsN
02060 5/9/16
(c) Kanehisa Laboratories

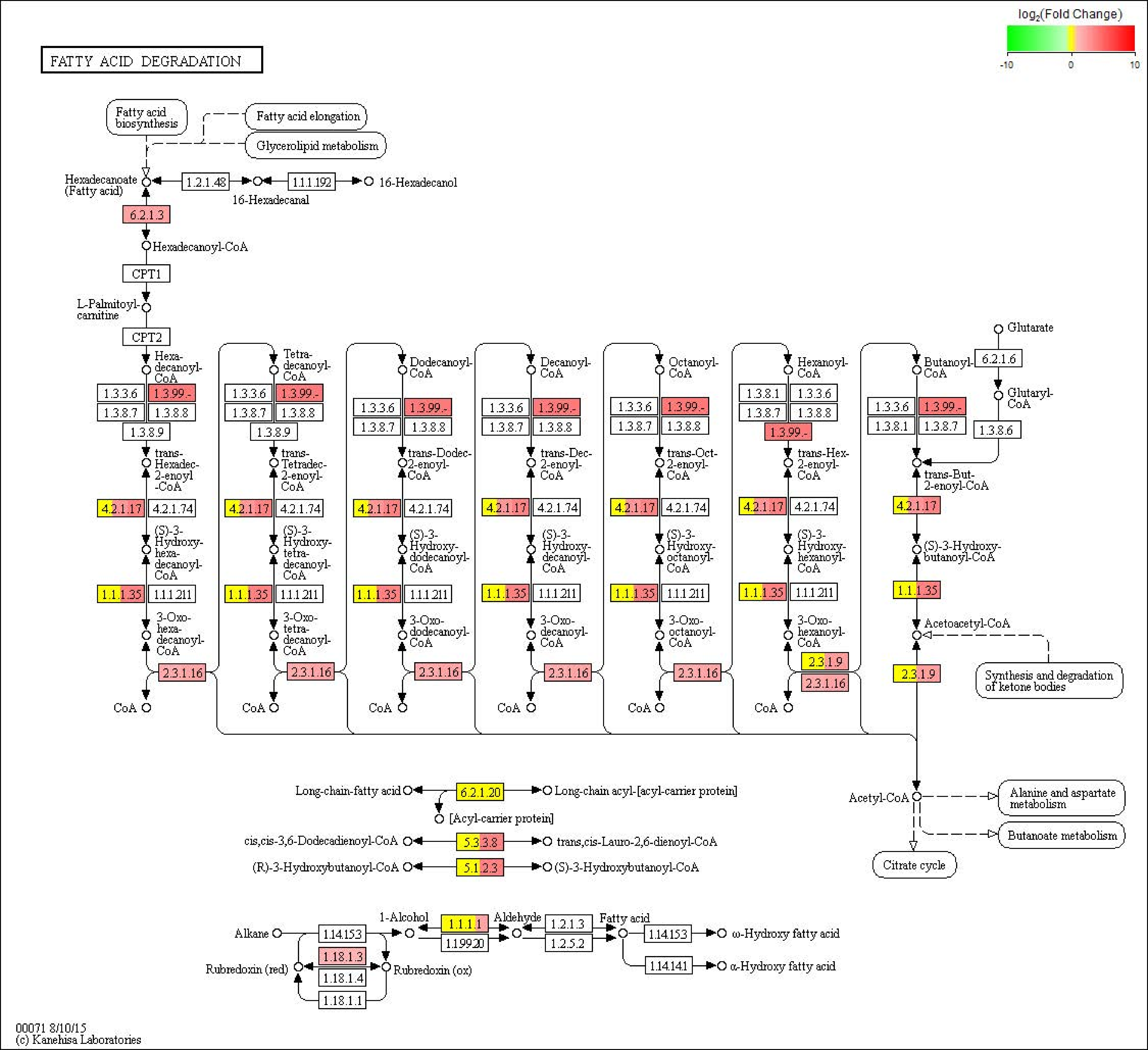

log2(Fold Change)
-10
0
10
FATTY ACID DEGRADATION
Fatty acid biosynthesis
Fatty acid elongation
Glycerolipid metabolism
Hexadecanoate (Fatty acid)
1.2.1.48
16-Hexadecanal
1.1.1.192
16-Hexadecanol
6.2.1.3
Hexadecanoyl-CoA
CPT1
L-Palmitoyl-carnitine
CPT2
Hexa-decanoyl-CoA
Tetra-decanoyl-CoA
Dodecanoyl-CoA
Decanoyl-CoA
Octanoyl-CoA
Hexanoyl-CoA
Butanoyl-CoA
Glutarate
6.2.1.6
Glutaryl-CoA
1.3.8.6
1.3.3.6
1.3.99.-
1.3.8.7
1.3.8.8
1.3.8.9
1.3.8.1
trans-Hexadec-2-enoyl-CoA
trans-Tetradec-2-enoyl-CoA
trans-Dodec-2-enoyl-CoA
trans-Dec-2-enoyl-CoA
trans-Oct-2-enoyl-CoA
trans-Hex-2-enoyl-CoA
trans-But-2-enoyl-CoA
4.2.1.17
4.2.1.74
(S)-3-Hydroxy-hexa-decanoyl-CoA
(S)-3-Hydroxy-tetra-decanoyl-CoA
(S)-3-Hydroxy-dodecanoyl-CoA
(S)-3-Hydroxy-decanoyl-CoA
(S)-3-Hydroxy-octanoyl-CoA
(S)-3-Hydroxy-hexanoyl-CoA
(S)-3-Hydroxy-butanoyl-CoA
1.1.1.35
1.1.1.211
3-Oxo-hexa-decanoyl-CoA
3-Oxo-tetra-decanoyl-CoA
3-Oxo-dodecanoyl-CoA
3-Oxo-decanoyl-CoA
3-Oxo-octanoyl-CoA
3-Oxo-hexanoyl-CoA
Acetoacetyl-CoA
2.3.1.16
2.3.1.9
CoA
Synthesis and degradation of ketone bodies
Acetyl-CoA
Alanine and aspartate metabolism
Butanoate metabolism
Citrate cycle
Long-chain-fatty acid
6.2.1.20
Long-chain acyl-[acyl-carrier protein]
[Acyl-carrier protein]
cis,cis-3,6-Dodecadienoyl-CoA
5.3.3.8
trans,cis-Lauro-2,6-dienoyl-CoA
(R)-3-Hydroxybutanoyl-CoA
5.1.2.3
(S)-3-Hydroxybutanoyl-CoA
Alkane
1.14.15.3
1-Alcohol
1.1.1.1
1.1.99.20
Aldehyde
1.2.1.3
1.2.5.2
Fatty acid
ω-Hydroxy fatty acid
1.14.14.1
α-Hydroxy fatty acid
1.18.1.3
1.18.1.4
1.18.1.1
Rubredoxin (red)
Rubredoxin (ox)
00071 8/10/15
(c) Kanehisa Laboratories

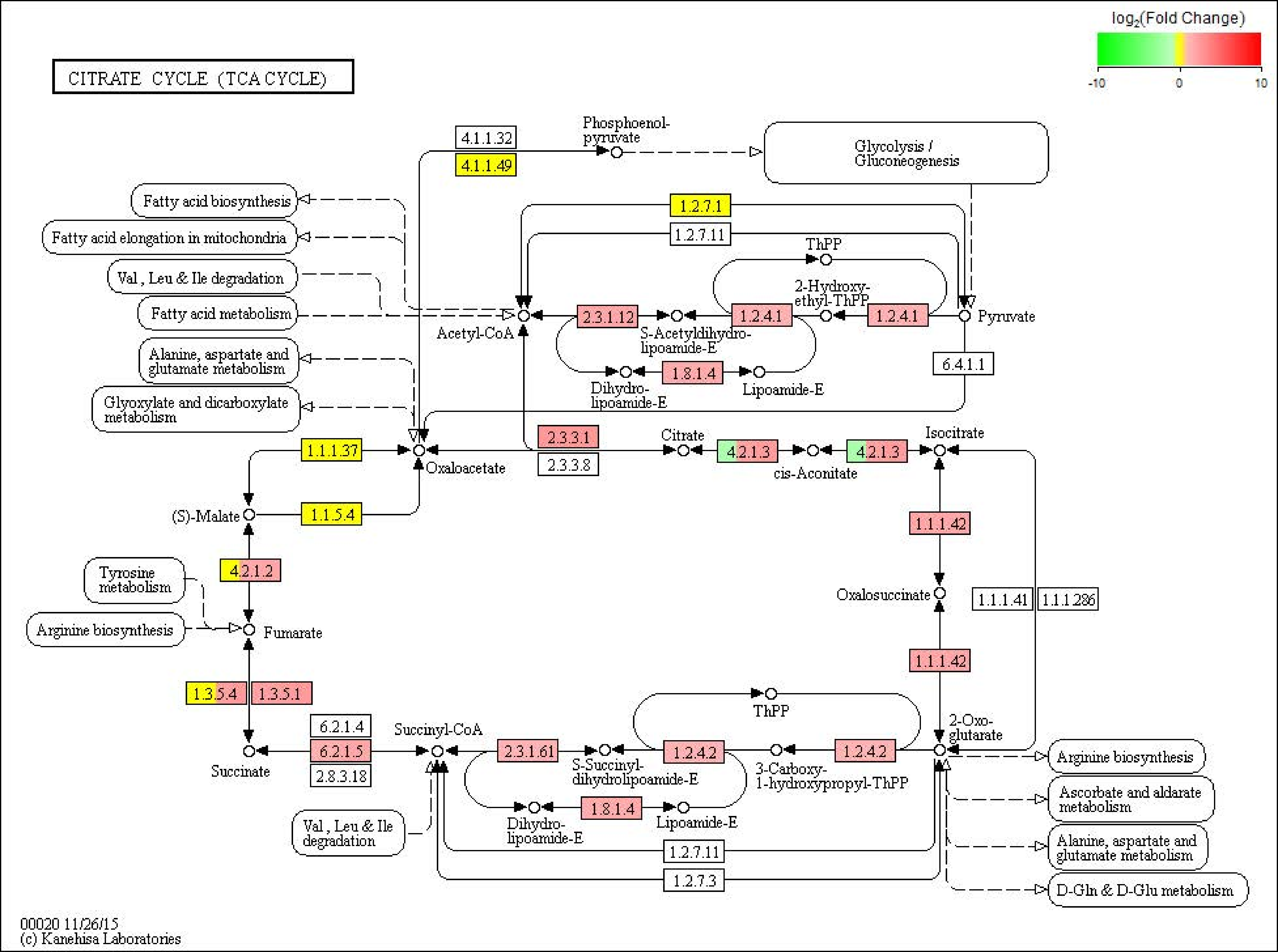

log2(Fold Change)
-10
0
10
CITRATE CYCLE (TCA CYCLE)
4.1.1.32
4.1.1.49
Phosphoenol-pyruvate
Glycolysis / Gluconeogenesis
Fatty acid biosynthesis
Fatty acid elongation in mitochondria
Val, Leu & Ile degradation
Fatty acid metabolism
Alanine, aspartate and glutamate metabolism
Glyoxylate and dicarboxylate metabolism
1.2.7.1
1.2.7.11
ThPP
2-Hydroxy-ethyl-ThPP
2.3.1.12
1.2.4.1
1.2.4.1
Pyruvate
Acetyl-CoA
S-Acetyldihydro-lipoamide-E
Dihydro-lipoamide-E
1.8.1.4
Lipoamide-E
6.4.1.1
2.3.3.1
2.3.3.8
Citrate
4.2.1.3
4.2.1.3
cis-Aconitate
Isocitrate
1.1.1.37
Oxaloacetate
(S)-Malate
1.1.5.4
1.1.1.42
4.2.1.2
Tyrosine metabolism
Arginine biosynthesis
Fumarate
Oxalosuccinate
1.1.1.41
1.1.1.286
1.1.1.42
1.3.5.4
1.3.5.1
ThPP
6.2.1.4
6.2.1.5
2.8.3.18
Succinate
Succinyl-CoA
2.3.1.61
S-Succinyl-dihydrolipoamide-E
1.2.4.2
1.2.4.2
3-Carboxy-1-hydroxypropyl-ThPP
2-Oxo-glutarate
Arginine biosynthesis
Ascorbate and aldarate metabolism
Alanine, aspartate and glutamate metabolism
D-Gln & D-Glu metabolism
Val, Leu & Ile degradation
Dihydro-lipoamide-E
1.8.1.4
Lipoamide-E
1.2.7.11
1.2.7.3
00020 11/26/15
(c) Kanehisa Laboratories

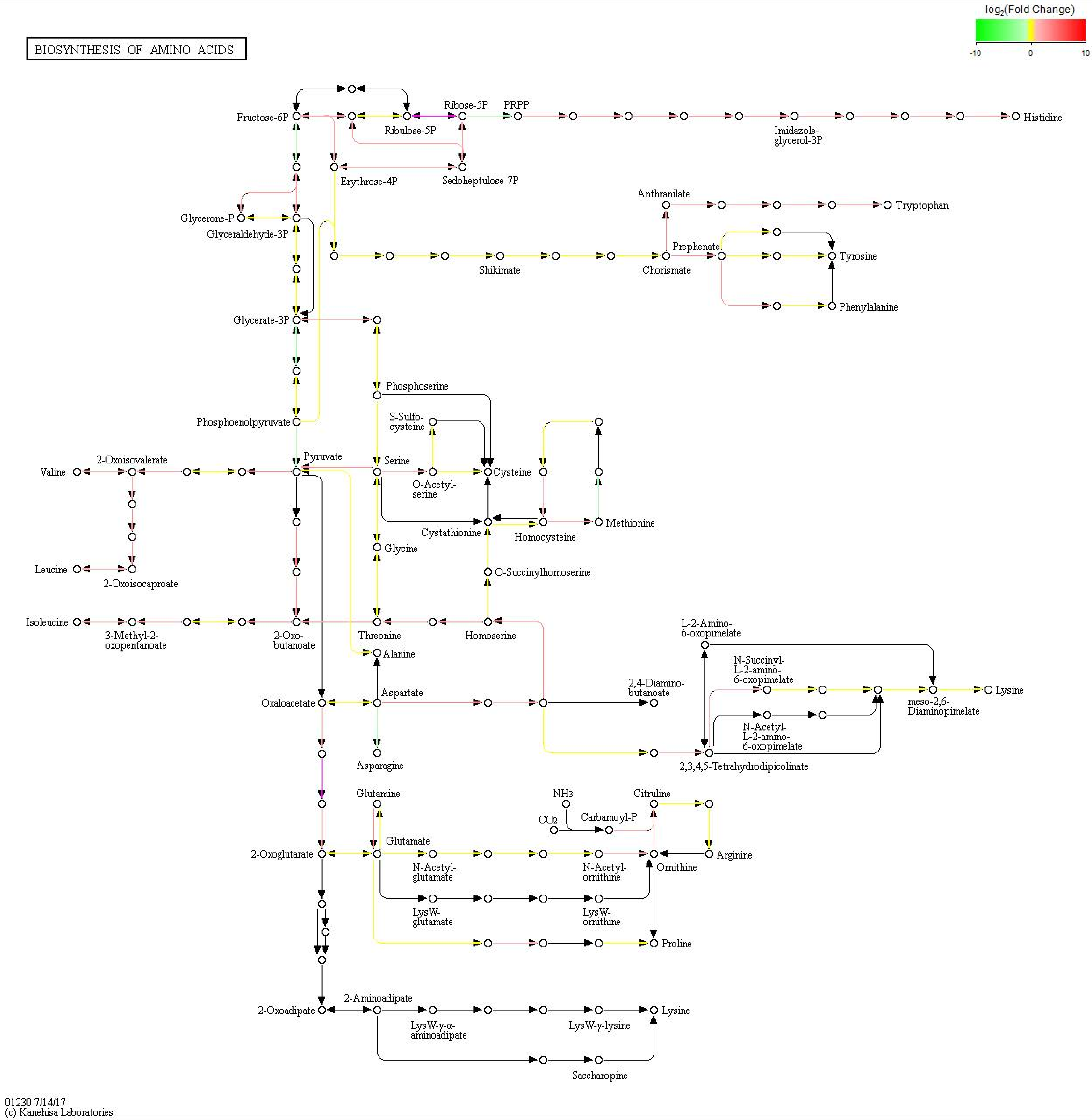

BIOSYNTHESIS OF AMINO ACIDS
log2(Fold Change)
-10
0
10
Fructose-6P
Ribulose-5P
Ribose-5P
PRPP
Histidine
Imidazole-glycerol-3P
Erythrose-4P
Sedoheptulose-7P
Anthranilate
Tryptophan
Glycerone-P
Glyceraldehyde-3P
Prephenate
Tyrosine
Shikimate
Chorismate
Phenylalanine
Glycerate-3P
Phosphoserine
S-Sulfo-cysteine
Phosphoenolpyruvate
2-Oxoisovalerate
Valine
Pyruvate
Serine
O-Acetyl-serine
Cysteine
Methionine
Cystathionine
Homocysteine
Glycine
Leucine
2-Oxoisocaproate
O-Succinylhomoserine
Isoleucine
3-Methyl-2-oxopentanoate
2-Oxo-butanoate
Threonine
Homoserine
L-2-Amino-6-oxopimelate
Alanine
N-Succinyl-L-2-amino-6-oxopimelate
Oxaloacetate
Aspartate
2,4-Diamino-butanoate
Lysine
meso-2,6-Diaminopimelate
N-Acetyl-L-2-amino-6-oxopimelate
Asparagine
2,3,4,5-Tetrahydrodipicolinate
Glutamine
NH3
Citruline
CO2
Carbamoyl-P
2-Oxoglutarate
Glutamate
Arginine
N-Acetyl-glutamate
N-Acetyl-ornithine
Ornithine
LysW-glutamate
LysW-ornithine
Proline
2-Aminoadipate
2-Oxoadipate
Lysine
LysW-γ-α-aminoadipate
LysW-γ-lysine
Saccharopine
01230 7/14/17
(c) Kanehisa Laboratories

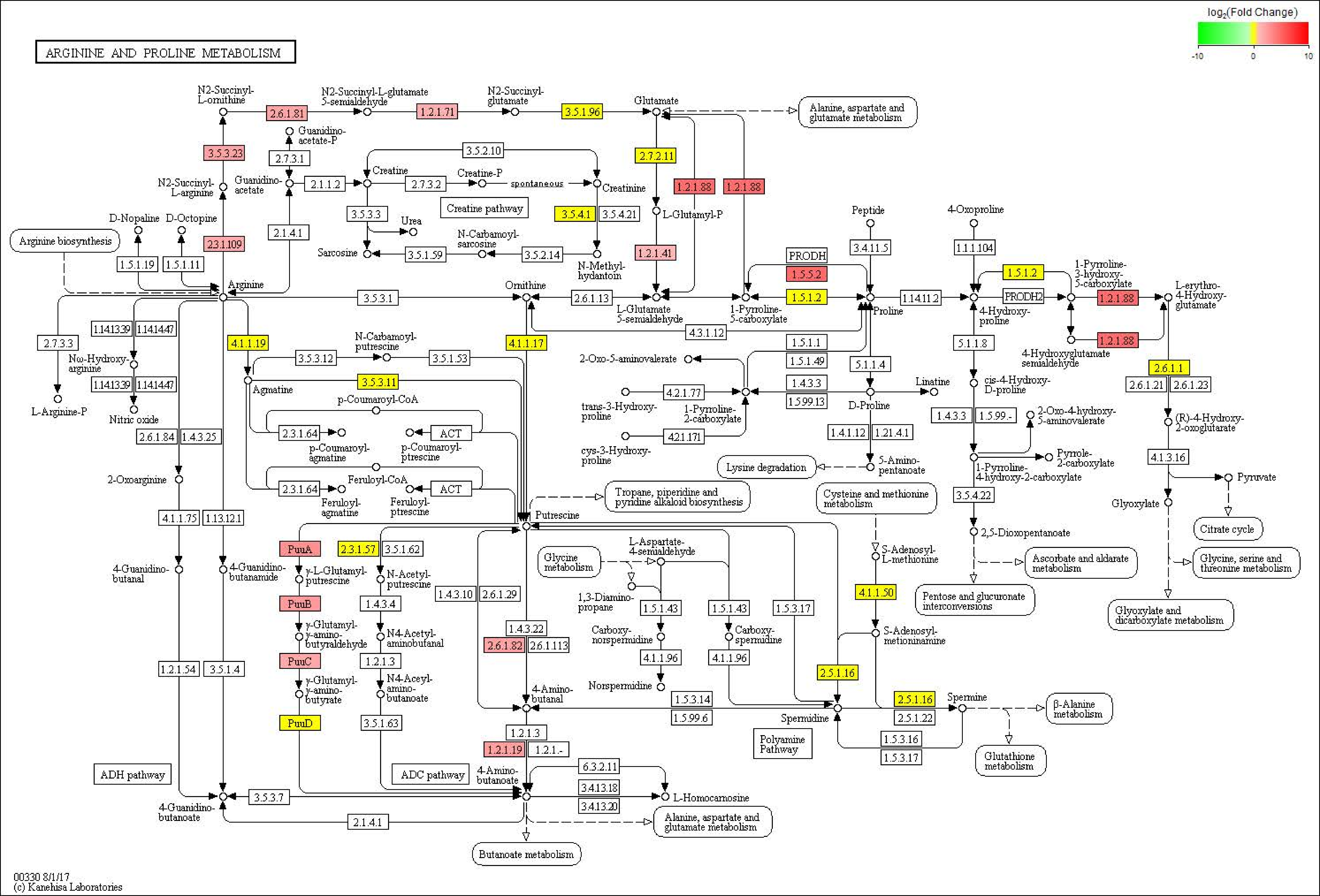

ARGININE AND PROLINE METABOLISM
log2(Fold Change)
-10
0
10
N2-Succinyl-L-ornithine
2.6.1.81
N2-Succinyl-L-glutamate 5-semialdehyde
1.2.1.71
N2-Succinyl-glutamate
3.5.1.96
Glutamate
Alanine, aspartate and glutamate metabolism
3.5.3.23
Guanidino-acetate-P
2.7.3.1
3.5.2.10
Creatine
Creatine-P
spontaneous
Creatinine
N2-Succinyl-L-arginine
Guanidino-acetate
2.1.1.2
2.7.3.2
2.7.2.11
1.2.1.88
1.2.1.88
Creatine pathway
3.5.3.3
Urea
3.5.4.1
3.5.4.21
L-Glutamyl-P
D-Nopaline
D-Octopine
Arginine biosynthesis
2.3.1.109
2.1.4.1
N-Carbamoyl-sarcosine
Sarcosine
3.5.1.59
3.5.2.14
N-Methyl-hydantoin
1.2.1.41
Peptide
4-Oxoproline
3.4.11.5
1.1.1.104
1.5.1.19
1.5.1.11
PRODH
1.5.5.2
1.5.1.2
1-Pyrroline-3-hydroxy-5-carboxylate
Arginine
Ornithine
3.5.3.1
2.6.1.13
L-Glutamate 5-semialdehyde
1.5.1.2
1-Pyrroline-5-carboxylate
Proline
1.14.11.2
PRODH2
4-Hydroxy-proline
1.2.1.88
L-erythro-4-Hydroxy-glutamate
4.3.1.12
2.7.3.3
1.14.13.39
1.14.14.47
4.1.1.19
N-Carbamoyl-putrescine
4.1.1.17
1.5.1.1
1.5.1.49
5.1.1.8
4-Hydroxyglutamate semialdehyde
1.2.1.88
Nω-Hydroxy-arginine
3.5.3.12
3.5.1.53
2-Oxo-5-aminovalerate
5.1.1.4
2.6.1.1
L-Arginine-P
1.14.13.39
1.14.14.47
Agmatine
3.5.3.11
1.4.3.3
Linatine
2.6.1.21
2.6.1.23
Nitric oxide
p-Coumaroyl-CoA
trans-3-Hydroxy-proline
4.2.1.77
1-Pyrroline-2-carboxylate
1.5.99.13
D-Proline
cis-4-Hydroxy-D-proline
1.4.3.3
1.5.99.-
2-Oxo-4-hydroxy-5-aminovalerate
(R)-4-Hydroxy-2-oxoglutarate
2.6.1.84
1.4.3.25
2.3.1.64
ACT
p-Coumaroyl-agmatine
p-Coumaroyl-ptrescine
cys-3-Hydroxy-proline
4.2.1.171
1.4.1.12
1.21.4.1
5-Amino-pentanoate
Lysine degradation
Pyrrole-2-carboxylate
1-Pyrroline-4-hydroxy-2-carboxylate
4.1.3.16
Pyruvate
2-Oxoarginine
Feruloyl-CoA
2.3.1.64
ACT
Feruloyl-agmatine
Feruloyl-ptrescine
Tropane, piperidine and pyridine alkaloid biosynthesis
Cysteine and methionine metabolism
3.5.4.22
Glyoxylate
4.1.1.75
1.13.12.1
Putrescine
2,5-Dioxopentanoate
Citrate cycle
PuuA
2.3.1.57
3.5.1.62
L-Aspartate-4-semialdehyde
Glycine metabolism
S-Adenosyl-L-methionine
Ascorbate and aldarate metabolism
Glycine, serine and threonine metabolism
4-Guanidino-butanal
4-Guanidino-butanamide
γ-L-Glutamyl-putrescine
N-Acetyl-putrescine
1,3-Diamino-propane
PuuB
1.4.3.4
1.4.3.10
2.6.1.29
1.5.1.43
1.5.1.43
1.5.3.17
4.1.1.50
Pentose and glucuronate interconversions
Glyoxylate and dicarboxylate metabolism
γ-Glutamyl-γ-amino-butyraldehyde
N4-Acetyl-aminobutanal
1.4.3.22
Carboxy-norspermidine
Carboxy-spermidine
S-Adenosyl-metioninamine
2.6.1.82
2.6.1.113
4.1.1.96
4.1.1.96
PuuC
1.2.1.3
N4-Aceyl-amino-butanoate
1.2.1.54
3.5.1.4
γ-Glutamyl-γ-amino-butyrate
4-Amino-butanal
Norspermidine
2.5.1.16
1.5.3.14
1.5.99.6
2.5.1.16
Spermine
β-Alanine metabolism
Spermidine
2.5.1.22
PuuD
3.5.1.63
1.2.1.3
Polyamine Pathway
1.5.3.16
1.5.3.17
1.2.1.19
1.2.1.-
Glutathione metabolism
ADH pathway
ADC pathway
4-Amino-butanoate
6.3.2.11
3.4.13.18
L-Homocarnosine
3.5.3.7
3.4.13.20
4-Guanidino-butanoate
2.1.4.1
Alanine, aspartate and glutamate metabolism
Butanoate metabolism
00330 8/1/17
(c) Kanehisa Laboratories

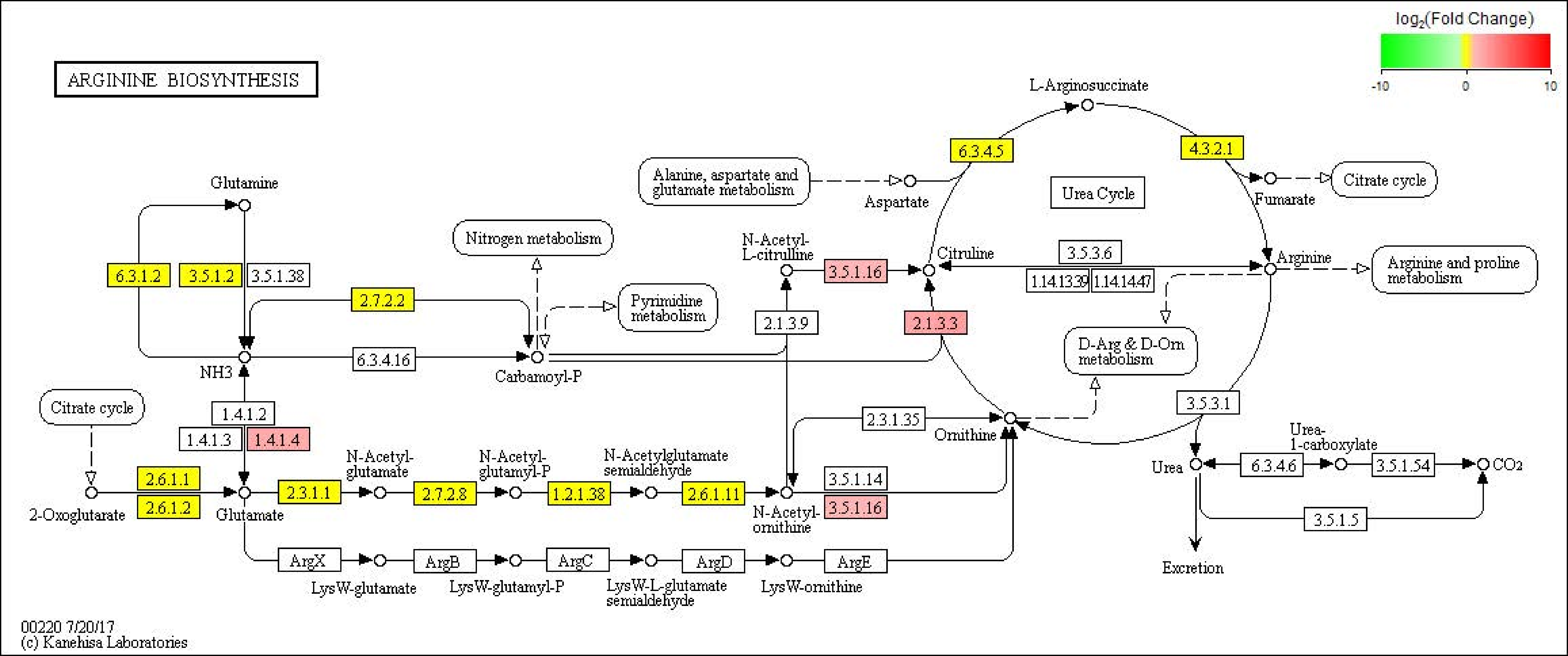
ARGININE BIOSYNTHESIS
log2(Fold Change)
-10
0
10
L-Arginosuccinate
6.3.4.5
4.3.2.1
Alanine, aspartate and glutamate metabolism
Aspartate
Citrate cycle
Fumarate
Urea Cycle
Glutamine
Nitrogen metabolism
N-Acetyl-L-citrulline
Citruline
3.5.3.6
Arginine
Arginine and proline metabolism
6.3.1.2
3.5.1.2
3.5.1.38
3.5.1.16
1.14.13.39
1.14.14.47
2.7.2.2
Pyrimidine metabolism
2.1.3.9
2.1.3.3
D-Arg & D-Orn metabolism
NH3
6.3.4.16
Carbamoyl-P
Citrate cycle
1.4.1.2
1.4.1.3
1.4.1.4
2.3.1.35
Ornithine
3.5.3.1
Urea-1-carboxylate
N-Acetyl-glutamate
N-Acetyl-glutamyl-P
N-Acetylglutamate semialdehyde
3.5.1.14
Urea
6.3.4.6
3.5.1.54
CO2
2.6.1.1
2.6.1.2
2.3.1.1
2.7.2.8
1.2.1.38
2.6.1.11
3.5.1.16
2-Oxoglutarate
Glutamate
N-Acetyl-ornithine
3.5.1.5
Excretion
ArgX
ArgB
ArgC
ArgD
ArgE
LysW-glutamate
LysW-glutamyl-P
LysW-L-glutamate semialdehyde
LysW-ornithine
00220 7/20/17
(c) Kanehisa Laboratories

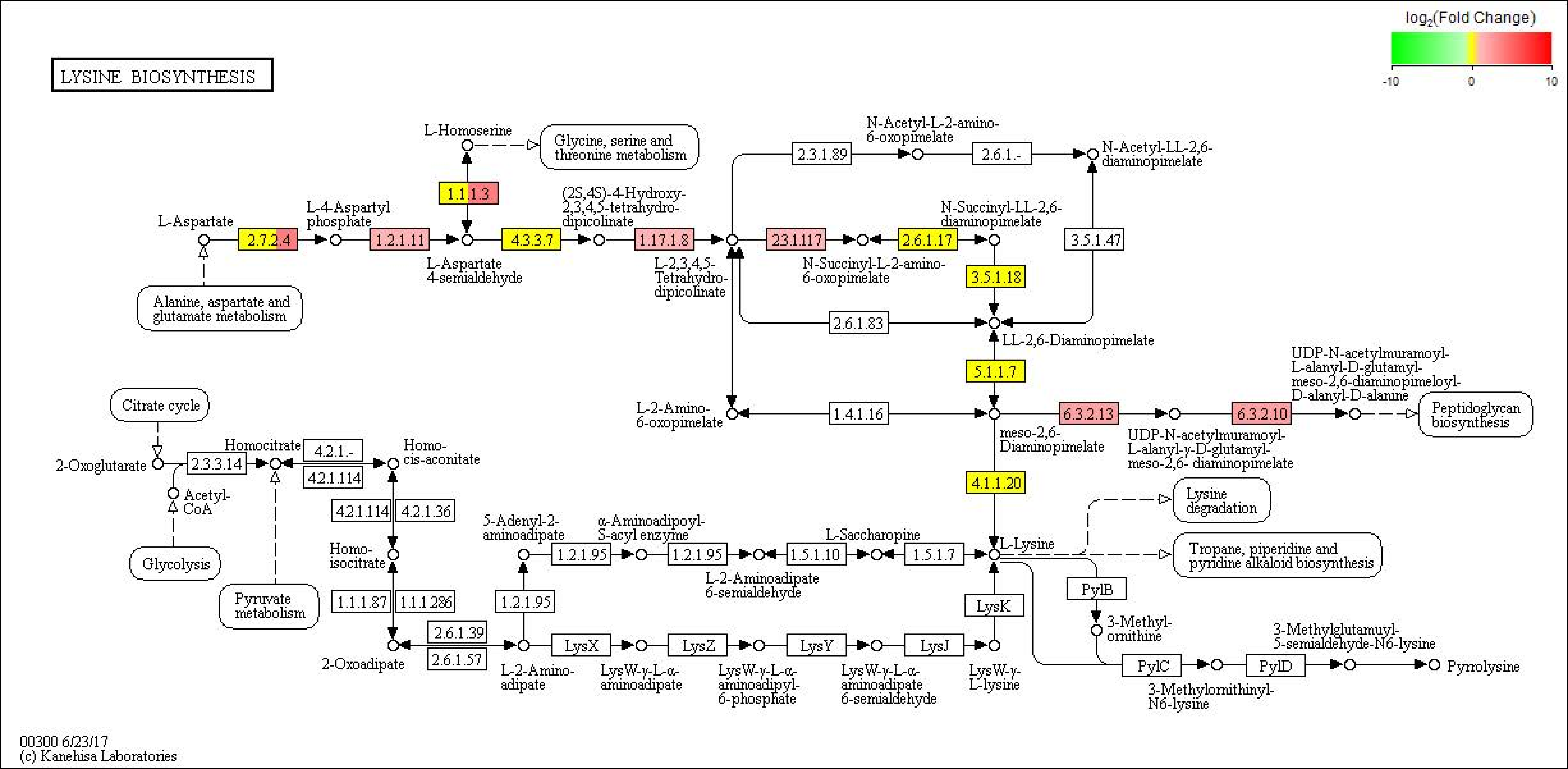

log2(Fold Change)
-10
0
10
LYSINE BIOSYNTHESIS
L-Homoserine
Glycine, serine and threonine metabolism
1.1.1.3
L-Aspartate
2.7.2.4
L-4-Aspartyl phosphate
1.2.1.11
L-Aspartate 4-semialdehyde
4.3.3.7
(2S,4S)-4-Hydroxy-2,3,4,5-tetrahydro-dipicolinate
1.17.1.8
L-2,3,4,5-Tetrahydro-dipicolinate
Alanine, aspartate and glutamate metabolism
2.3.1.89
N-Acetyl-L-2-amino-6-oxopimelate
2.6.1.-
N-Acetyl-LL-2,6-diaminopimelate
2.3.1.117
N-Succinyl-L-2-amino-6-oxopimelate
2.6.1.17
N-Succinyl-LL-2,6-diaminopimelate
3.5.1.47
3.5.1.18
2.6.1.83
LL-2,6-Diaminopimelate
5.1.1.7
L-2-Amino-6-oxopimelate
1.4.1.16
meso-2,6-Diaminopimelate
6.3.2.13
UDP-N-acetylmuramoyl-L-alanyl-γ-D-glutamyl-meso-2,6- diaminopimelate
6.3.2.10
UDP-N-acetylmuramoyl-L-alanyl-D-glutamyl-meso-2,6-diaminopimeloyl-D-alanyl-D-alanine
Peptidoglycan biosynthesis
4.1.1.20
Citrate cycle
2-Oxoglutarate
2.3.3.14
Homocitrate
4.2.1.-
4.2.1.114
Homo-cis-aconitate
Acetyl-CoA
Glycolysis
Pyruvate metabolism
4.2.1.114
4.2.1.36
Homo-isocitrate
1.1.1.87
1.1.1.286
2-Oxoadipate
2.6.1.39
2.6.1.57
5-Adenyl-2-aminoadipate
1.2.1.95
α-Aminoadipoyl-S-acyl enzyme
1.2.1.95
L-2-Aminoadipate 6-semialdehyde
1.5.1.10
L-Saccharopine
1.5.1.7
L-Lysine
Lysine degradation
Tropane, piperidine and pyridine alkaloid biosynthesis
1.2.1.95
L-2-Amino-adipate
LysX
LysW-γ-L-α-aminoadipate
LysZ
LysW-γ-L-α-aminoadipyl-6-phosphate
LysY
LysW-γ-L-α-aminoadipate 6-semialdehyde
LysJ
LysW-γ-L-lysine
LysK
PylB
3-Methyl-ornithine
PylC
3-Methylornithinyl-N6-lysine
PylD
3-Methylglutamuyl-5-semialdehyde-N6-lysine
Pyrrolysine
00300 6/23/17
(c) Kanehisa Laboratories

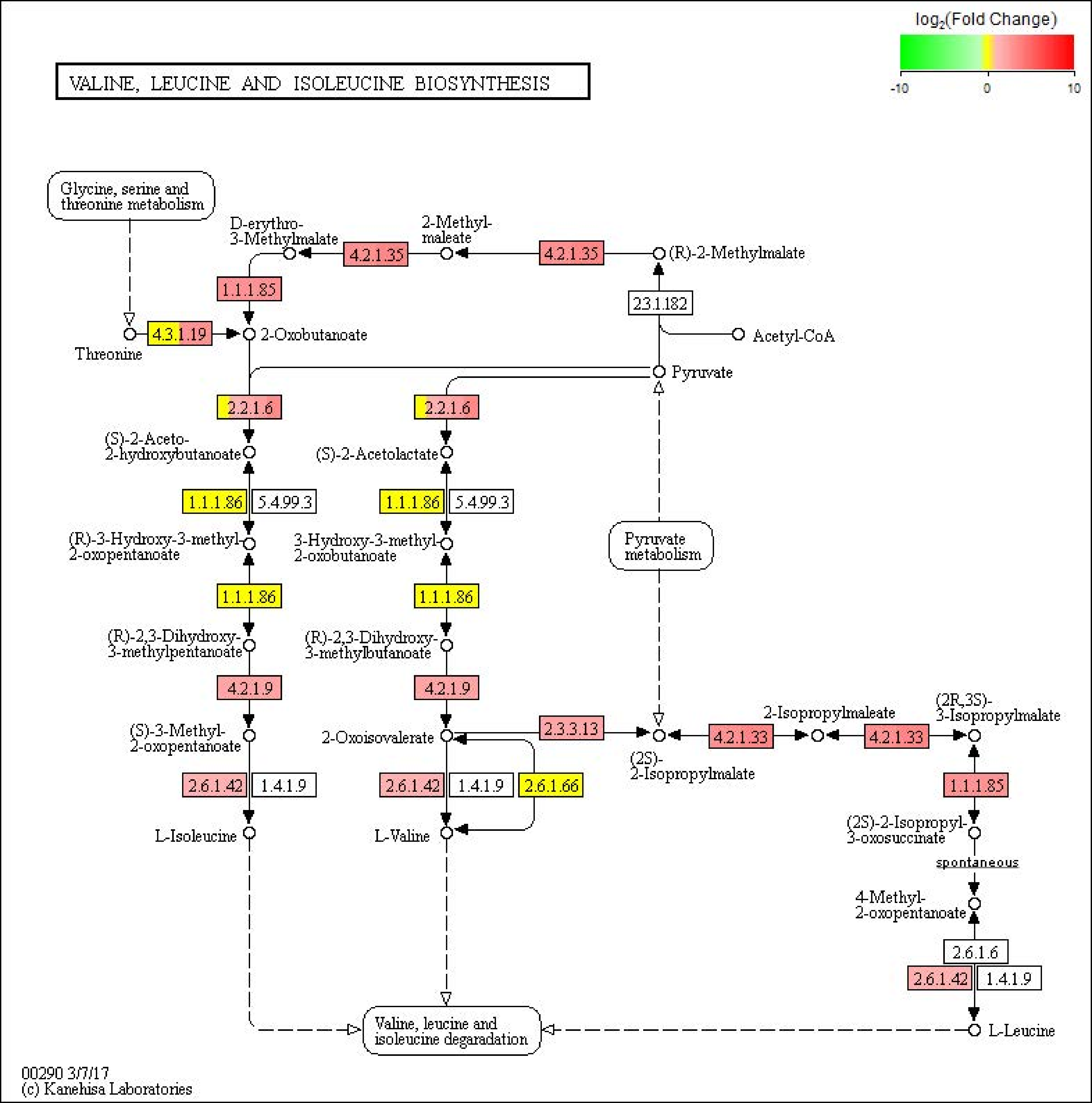

log2(Fold Change)
-10
0
10
VALINE, LEUCINE AND ISOLEUCINE BIOSYNTHESIS
Glycine, serine and threonine metabolism
D-erythro-3-Methylmalate
4.2.1.35
2-Methyl-maleate
4.2.1.35
(R)-2-Methylmalate
1.1.1.85
2.3.1.182
Threonine
4.3.1.19
2-Oxobutanoate
Acetyl-CoA
Pyruvate
2.2.1.6
2.2.1.6
(S)-2-Aceto-2-hydroxybutanoate
(S)-2-Acetolactate
1.1.1.86
5.4.99.3
1.1.1.86
5.4.99.3
(R)-3-Hydroxy-3-methyl-2-oxopentanoate
3-Hydroxy-3-methyl-2-oxobutanoate
Pyruvate metabolism
1.1.1.86
1.1.1.86
(R)-2,3-Dihydroxy-3-methylpentanoate
(R)-2,3-Dihydroxy-3-methylbutanoate
4.2.1.9
4.2.1.9
(S)-3-Methyl-2-oxopentanoate
2-Oxoisovalerate
2.3.3.13
(2S)-2-Isopropylmalate
4.2.1.33
2-Isopropylmaleate
4.2.1.33
(2R,3S)-3-Isopropylmalate
2.6.1.42
1.4.1.9
2.6.1.42
1.4.1.9
2.6.1.66
1.1.1.85
L-Isoleucine
L-Valine
(2S)-2-Isopropyl-3-oxosuccinate
spontaneous
4-Methyl-2-oxopentanoate
2.6.1.6
2.6.1.42
1.4.1.9
Valine, leucine and isoleucine degaradation
L-Leucine
00290 3/7/17
(c) Kanehisa Laboratories

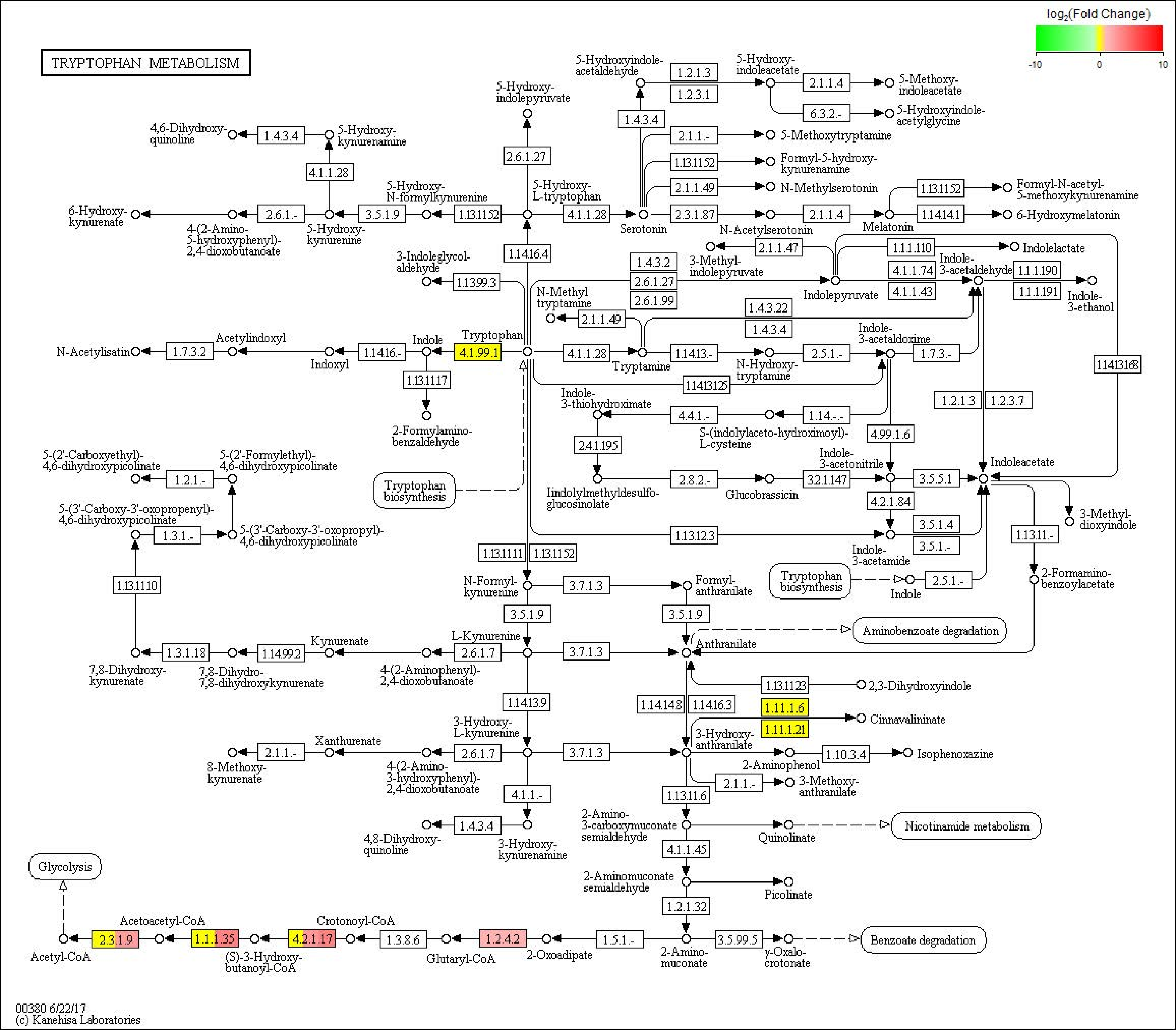
TRYPTOPHAN METABOLISM
log2(Fold Change)
-10
0
10
5-Hydroxyindole-acetaldehyde
1.2.1.3
1.2.3.1
5-Hydroxy-indoleacetate
2.1.1.4
5-Methoxy-indoleacetate
6.3.2.-
5-Hydroxyindole-acetylglycine
5-Hydroxy-indolepyruvate
1.4.3.4
2.1.1.-
5-Methoxytryptamine
1.13.11.52
Formyl-5-hydroxy-kynurenamine
2.1.1.49
N-Methylserotonin
1.13.11.52
Formyl-N-acetyl-5-methoxykynurenamine
4,6-Dihydroxy-quinoline
1.4.3.4
5-Hydroxy-kynurenamine
2.6.1.27
4.1.1.28
5-Hydroxy-N-formylkynurenine
5-Hydroxy-L-tryptophan
6-Hydroxy-kynurenate
2.6.1.-
3.5.1.9
1.13.11.52
4.1.1.28
2.3.1.87
2.1.1.4
1.14.14.1
6-Hydroxymelatonin
4-(2-Amino-5-hydroxyphenyl)-2,4-dioxobutanoate
5-Hydroxy-kynurenine
Serotonin
N-Acetylserotonin
Melatonin
1.14.16.4
2.1.1.47
1.1.1.110
Indolelactate
3-Indoleglycol-aldehyde
1.4.3.2
3-Methyl-indolepyruvate
4.1.1.74
Indole-3-acetaldehyde
1.1.1.190
1.13.99.3
2.6.1.27
N-Methyl tryptamine
2.6.1.99
Indolepyruvate
4.1.1.43
1.1.1.191
Indole-3-ethanol
2.1.1.49
1.4.3.22
1.4.3.4
Indole-3-acetaldoxime
Acetylindoxyl
Indole
Tryptophan
N-Acetylisatin
1.7.3.2
1.14.16.-
4.1.99.1
4.1.1.28
1.14.13.-
2.5.1.-
1.7.3.-
Indoxyl
Tryptamine
N-Hydroxy-tryptamine
1.13.11.17
1.14.13.125
1.14.13.168
Indole-3-thiohydroximate
2-Formylamino-benzaldehyde
4.4.1.-
1.14.-.-
1.2.1.3
1.2.3.7
S-(indolylaceto-hydroximoyl)-L-cysteine
2.4.1.195
4.99.1.6
5-(2'-Carboxyethyl)-4,6-dihydroxypicolinate
5-(2'-Formylethyl)-4,6-dihydroxypicolinate
Indoleacetate
1.2.1.-
Indole-3-acetonitrile
Tryptophan biosynthesis
2.8.2.-
3.2.1.147
3.5.5.1
Indolylmethyldesulfo-glucosinolate
Glucobrassicin
5-(3'-Carboxy-3'-oxopropenyl)-4,6-dihydroxypicolinate
4.2.1.84
3-Methyl-dioxyindole
1.3.1.-
5-(3'-Carboxy-3'-oxopropyl)-4,6-dihydroxypicolinate
3.5.1.4
1.13.12.3
1.13.11.-
3.5.1.-
1.13.11.11
1.13.11.52
Indole-3-acetamide
1.13.11.10
N-Formyl-kynurenine
3.7.1.3
Formyl-anthranilate
Tryptophan biosynthesis
Indole
2.5.1.-
2-Formamino-benzoylacetate
3.5.1.9
3.5.1.9
Aminobenzoate degradation
L-Kynurenine
Kynurenate
Anthranilate
1.3.1.18
1.14.99.2
2.6.1.7
3.7.1.3
7,8-Dihydroxy-kynurenate
7,8-Dihydro-7,8-dihydroxykynurenate
4-(2-Aminophenyl)-2,4-dioxobutanoate
1.13.11.23
2,3-Dihydroxyindole
1.14.13.9
1.14.14.8
1.14.16.3
1.11.1.6
Cinnavalininate
1.11.1.21
3-Hydroxy-L-kynurenine
3-Hydroxy-anthranilate
Xanthurenate
2.1.1.-
2.6.1.7
3.7.1.3
1.10.3.4
Isophenoxazine
8-Methoxy-kynurenate
4-(2-Amino-3-hydroxyphenyl)-2,4-dioxobutanoate
2-Aminophenol
2.1.1.-
3-Methoxy-anthranilate
4.1.1.-
1.13.11.6
1.4.3.4
2-Amino-3-carboxymuconate semialdehyde
Quinolinate
Nicotinamide metabolism
4,8-Dihydroxy-quinoline
3-Hydroxy-kynurenamine
4.1.1.45
Glycolysis
2-Aminomuconate semialdehyde
Picolinate
1.2.1.32
Acetoacetyl-CoA
Crotonoyl-CoA
2.3.1.9
1.1.1.35
4.2.1.17
1.3.8.6
1.2.4.2
1.5.1.-
3.5.99.5
Benzoate degradation
Acetyl-CoA
(S)-3-Hydroxy-butanoyl-CoA
Glutaryl-CoA
2-Oxoadipate
2-Amino-muconate
γ-Oxalo-crotonate
00380 6/22/17
(c) Kanehisa Laboratories

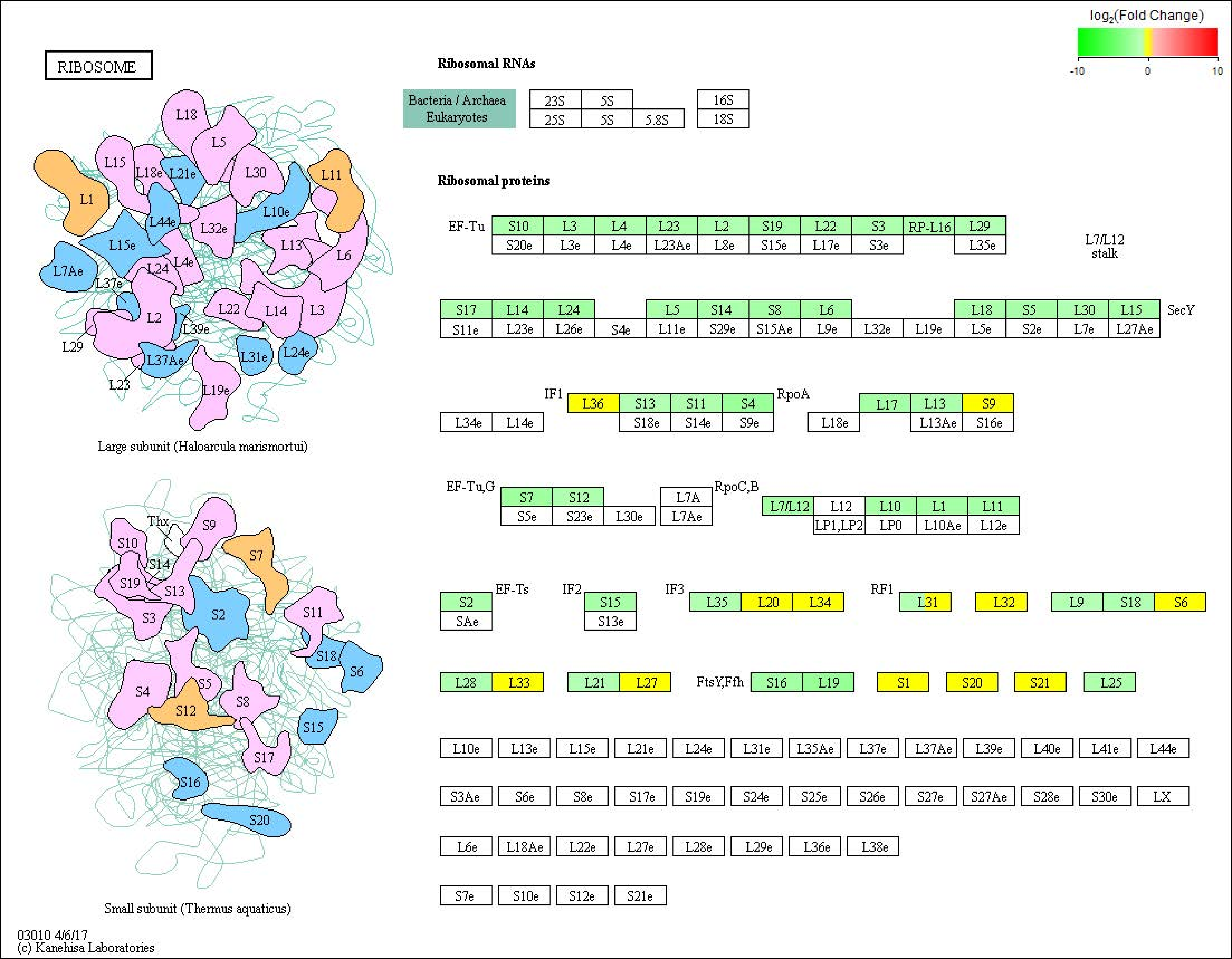

RIBOSOME
log2(Fold Change)
-10
0
10
Ribosomal RNAs
Bacteria / Archaea
Eukaryotes
23S
5S
25S
5S
5.8S
16S
18S
Ribosomal proteins
EF-Tu
S10 L3 L4 L23 L2 S19 L22 S3 RP-L16 L29
S20e L3e L4e L23Ae L8e S15e L17e S3e L35e
L7/L12 stalk
S17 L14 L24 L5 S14 S8 L6 L18 S5 L30 L15
S11e L23e L26e S4e L11e S29e S15Ae L9e L32e L19e L5e S2e L7e L27Ae
SecY
IF1
L36 S13 S11 S4
L34e L14e S18e S14e S9e
RpoA
L17 L13 S9
L18e L13Ae S16e
EF-Tu,G
S7 S12
S5e S23e L30e
L7A
L7Ae
RpoC,B
L7/L12 L12 L10 L1 L11
LP1,LP2 LP0 L10Ae L12e
S2
SAe
EF-Ts
IF2
S15
S13e
IF3
L35 L20 L34
RF1
L31
L32
L9 S18 S6
L28 L33
L21 L27
FtsY,Ffh
S16 L19
S1
S20
S21
L25
L10e L13e L15e L21e L24e L31e L35Ae L37e L37Ae L39e L40e L41e L44e
S3Ae S6e S8e S17e S19e S24e S25e S26e S27e S27Ae S28e S30e LX
L6e L18Ae L22e L27e L28e L29e L36e L38e
S7e S10e S12e S21e
L18 L5 L15 L18e L21e L30 L1 L11 L10e L44e L32e L15e L13 L6 L7Ae L4e L24 L37e L2 L22 L14 L3 L29 L39e L31e L24e L37Ae L23 L19e
Large subunit (Haloarcula marismortui)
Thx S9 S10 S14 S7 S19 S13 S3 S2 S11 S18 S6 S4 S5 S8 S12 S15 S17 S16 S20
Small subunit (Thermus aquaticus)
03010 4/6/17
(c) Kanehisa Laboratories

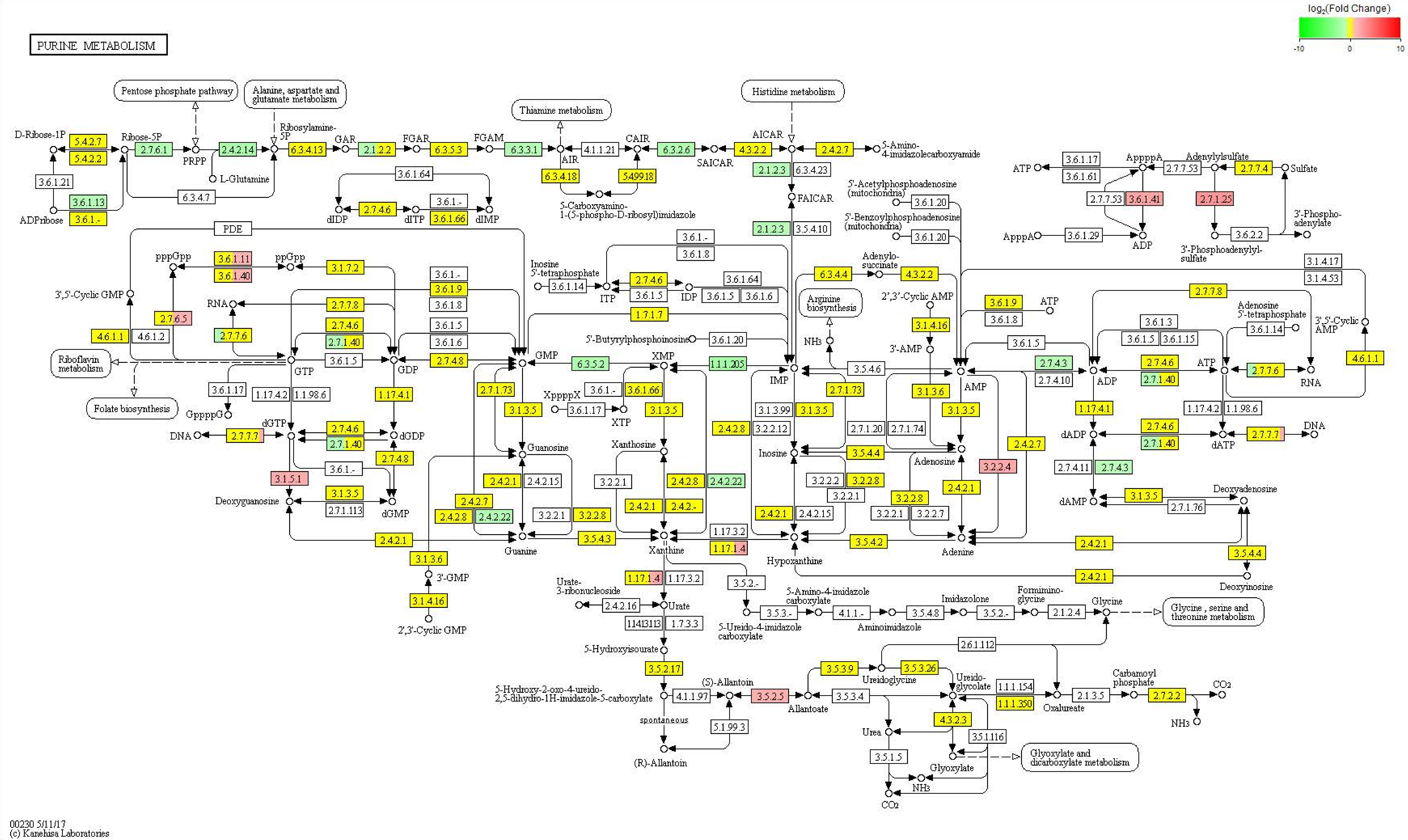

PURINE METABOLISM
log2(Fold Change)
-10
0
10
Pentose phosphate pathway
Alanine, aspartate and glutamate metabolism
Thiamine metabolism
Histidine metabolism
D-Ribose-1P
5.4.2.7
5.4.2.2
Ribose-5P
2.7.6.1
PRPP
2.4.2.14
L-Glutamine
Ribosylamine-5P
6.3.4.13
GAR
2.1.2.2
FGAR
6.3.5.3
FGAM
6.3.3.1
AIR
4.1.1.21
CAIR
6.3.2.6
SAICAR
4.3.2.2
AICAR
2.4.2.7
5-Amino-4-imidazolecarboxyamide
6.3.4.18
5.4.99.18
5-Carboxyamino-1-(5-phospho-D-ribosyl)imidazole
2.1.2.3
6.3.4.23
FAICAR
2.1.2.3
3.5.4.10
3.6.1.21
3.6.1.13
ADPribose
3.6.1.-
6.3.4.7
3.6.1.64
dIDP
2.7.4.6
dITP
3.6.1.-
3.6.1.66
dIMP
PDE
pppGpp
3.6.1.11
3.6.1.40
ppGpp
3.1.7.2
3',5'-Cyclic GMP
2.7.6.5
4.6.1.1
4.6.1.2
RNA
2.7.7.6
2.7.7.8
2.7.4.6
2.7.1.40
3.6.1.-
3.6.1.9
3.6.1.8
3.6.1.5
3.6.1.6
Riboflavin metabolism
Folate biosynthesis
GTP
3.6.1.5
GDP
2.7.4.8
GMP
6.3.5.2
XMP
1.1.1.205
IMP
3.6.1.17
GppppG
1.17.4.2
1.1.98.6
1.17.4.1
dGTP
DNA
2.7.7.7
2.7.4.6
2.7.1.40
dGDP
2.7.4.8
3.6.1.-
3.1.5.1
3.1.3.5
Deoxyguanosine
2.7.1.113
dGMP
2.7.1.73
3.1.3.5
Guanosine
2.4.2.1
2.4.2.15
2.4.2.7
2.4.2.8
2.4.2.22
3.2.2.1
3.2.2.8
Guanine
3.5.4.3
2.4.2.1
3.1.3.6
3'-GMP
3.1.4.16
2',3'-Cyclic GMP
Inosine 5'-tetraphosphate
3.6.1.14
ITP
2.7.4.6
3.6.1.5
IDP
3.6.1.64
3.6.1.5
3.6.1.6
3.6.1.-
3.6.1.8
1.7.1.7
5'-Butyrylphosphoinosine
3.6.1.20
XppppX
3.6.1.17
XTP
3.6.1.-
3.6.1.66
3.1.3.5
Xanthosine
3.2.2.1
2.4.2.8
2.4.2.22
2.4.2.1
2.4.2.-
Xanthine
1.17.3.2
1.17.1.4
Urate-3-ribonucleoside
2.4.2.16
Urate
1.17.1.4
1.17.3.2
1.14.13.113
1.7.3.3
5-Hydroxyisourate
3.5.2.17
5-Hydroxy-2-oxo-4-ureido-2,5-dihydro-1H-imidazole-5-carboxylate
4.1.1.97
spontaneous
(R)-Allantoin
(S)-Allantoin
5.1.99.3
3.5.2.5
Allantoate
3.5.2.-
5-Ureido-4-imidazole carboxylate
3.5.3.-
5-Amino-4-imidazole carboxylate
4.1.1.-
Aminoimidazole
3.5.4.8
Imidazolone
3.5.2.-
Formimino-glycine
2.1.2.4
Glycine
Glycine, serine and threonine metabolism
2.6.1.112
3.5.3.9
Ureidoglycine
3.5.3.26
Ureido-glycolate
1.1.1.154
1.1.1.350
Oxalureate
2.1.3.5
Carbamoyl phosphate
2.7.2.2
CO2
NH3
3.5.3.4
4.3.2.3
3.5.1.116
Urea
3.5.1.5
Glyoxylate
Glyoxylate and dicarboxylate metabolism
NH3
CO2
3.1.3.99
3.1.3.5
3.2.2.12
2.4.2.8
Inosine
3.5.4.4
3.2.2.2
3.2.2.8
3.2.2.1
2.4.2.1
2.4.2.15
Hypoxanthine
3.5.4.2
Arginine biosynthesis
NH3
3.5.4.6
6.3.4.4
Adenylo-succinate
4.3.2.2
2',3'-Cyclic AMP
3.1.4.16
3'-AMP
3.1.3.6
AMP
3.1.3.5
2.7.1.20
2.7.1.74
Adenosine
2.4.2.1
3.2.2.8
3.2.2.1
3.2.2.7
2.4.2.7
3.2.2.4
Adenine
5'-Acetylphosphoadenosine (mitochondria)
5'-Benzoylphosphoadenosine (mitochondria)
3.6.1.20
3.6.1.20
3.6.1.9
3.6.1.8
ATP
3.6.1.5
2.7.4.3
2.7.4.10
ADP
2.7.4.6
2.7.1.40
ATP
2.7.7.6
RNA
3.6.1.3
3.6.1.5
3.6.1.15
Adenosine 5'-tetraphosphate
3.6.1.14
2.7.7.8
3',5'-Cyclic AMP
4.6.1.1
3.1.4.17
3.1.4.53
1.17.4.1
1.17.4.2
1.1.98.6
dADP
2.7.4.6
2.7.1.40
dATP
2.7.7.7
DNA
2.7.4.11
2.7.4.3
dAMP
3.1.3.5
2.7.1.76
Deoxyadenosine
2.4.2.1
3.5.4.4
2.4.2.1
Deoxyinosine
ATP
3.6.1.17
3.6.1.61
ApppppA
2.7.7.53
Adenylylsulfate
2.7.7.4
Sulfate
2.7.7.53
3.6.1.41
2.7.1.25
ApppA
3.6.1.29
ADP
3'-Phosphoadenylyl-sulfate
3.6.2.2
3'-Phospho-adenylate
00230 5/11/17
(c) Kanehisa Laboratories

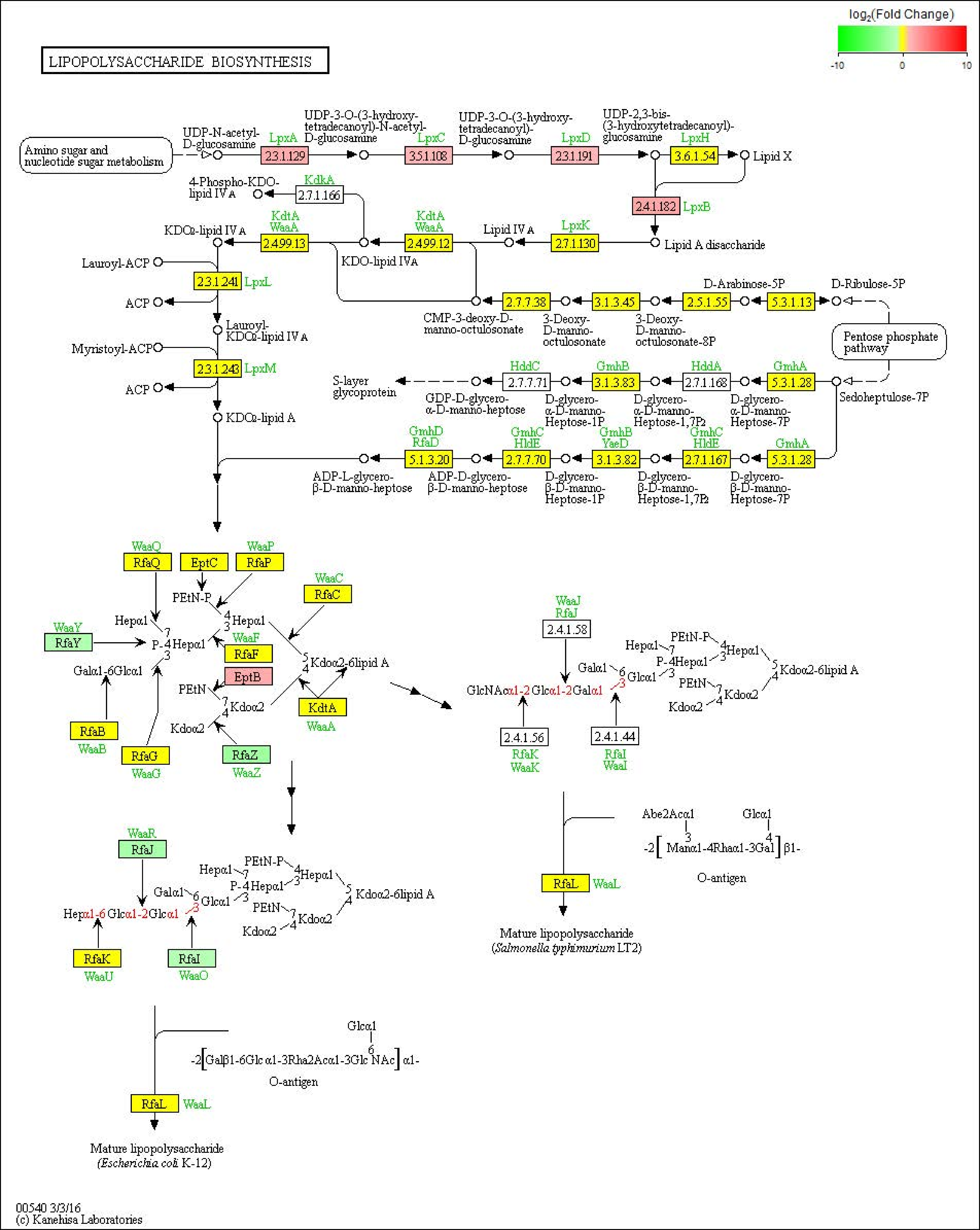

LIPOPOLYSACCHARIDE BIOSYNTHESIS
log2(Fold Change)
-10
0
10
Amino sugar and nucleotide sugar metabolism
UDP-N-acetyl-D-glucosamine
LpxA
2.3.1.129
UDP-3-O-(3-hydroxy-tetradecanoyl)-N-acetyl-D-glucosamine
LpxC
3.5.1.108
UDP-3-O-(3-hydroxy-tetradecanoyl)-D-glucosamine
LpxD
2.3.1.191
UDP-2,3-bis-(3-hydroxytetradecanoyl)-glucosamine
LpxH
3.6.1.54
Lipid X
LpxB
2.4.1.182
Lipid A disaccharide
LpxK
2.7.1.130
Lipid IV A
KdtA WaaA
2.4.99.12
KDO-lipid IVA
2.4.99.13
KDO2-lipid IV A
KdkA
2.7.1.166
4-Phospho-KDO-lipid IV A
Lauroyl-ACP
2.3.1.241
LpxL
ACP
Lauroyl-KDO2-lipid IV A
Myristoyl-ACP
2.3.1.243
LpxM
ACP
KDO2-lipid A
CMP-3-deoxy-D-manno-octulosonate
2.7.7.38
3-Deoxy-D-manno-octulosonate
3.1.3.45
3-Deoxy-D-manno-octulosonate-8P
2.5.1.55
D-Arabinose-5P
5.3.1.13
D-Ribulose-5P
Pentose phosphate pathway
Sedoheptulose-7P
S-layer glycoprotein
HddC
2.7.7.71
GDP-D-glycero-α-D-manno-heptose
GmhB
3.1.3.83
D-glycero-α-D-manno-Heptose-1P
HddA
2.7.1.168
D-glycero-α-D-manno-Heptose-1,7P2
GmhA
5.3.1.28
D-glycero-α-D-manno-Heptose-7P
GmhD RfaD
5.1.3.20
ADP-L-glycero-β-D-manno-heptose
GmhC HldE
2.7.7.70
ADP-D-glycero-β-D-manno-heptose
GmhB YaeD
3.1.3.82
D-glycero-β-D-manno-Heptose-1P
GmhC HldE
2.7.1.167
D-glycero-β-D-manno-Heptose-1,7P2
GmhA
5.3.1.28
D-glycero-β-D-manno-Heptose-7P
WaaQ RfaQ
EptC
WaaP RfaP
WaaC RfaC
WaaY RfaY
WaaF RfaF
EptB
KdtA WaaA
RfaB WaaB
RfaG WaaG
RfaZ WaaZ
PEtN-P
Hepα1
P-4Hepα1
Galα1-6Glcα1
PEtN
Kdoα2
Kdoα2-6lipid A
WaaJ RfaJ
2.4.1.58
GlcNAcα1-2 Glcα1-2Galα1
2.4.1.56
RfaK WaaK
2.4.1.44
RfaI WaaI
Abe2Acα1
Glcα1
-2[Manα1-4Rhaα1-3Gal]β1-
O-antigen
RfaL WaaL
Mature lipopolysaccharide (Salmonella typhimurium LT2)
WaaR RfaJ
Hepα1-6 Glcα1-2Glcα1
RfaK WaaU
RfaI WaaO
Glcα1
-2[Galβ1-6Glc α1-3Rha2Acα1-3Glc NAc]α1-
O-antigen
RfaL WaaL
Mature lipopolysaccharide (Escherichia coli K-12)
00540 3/3/16
(c) Kanehisa Laboratories

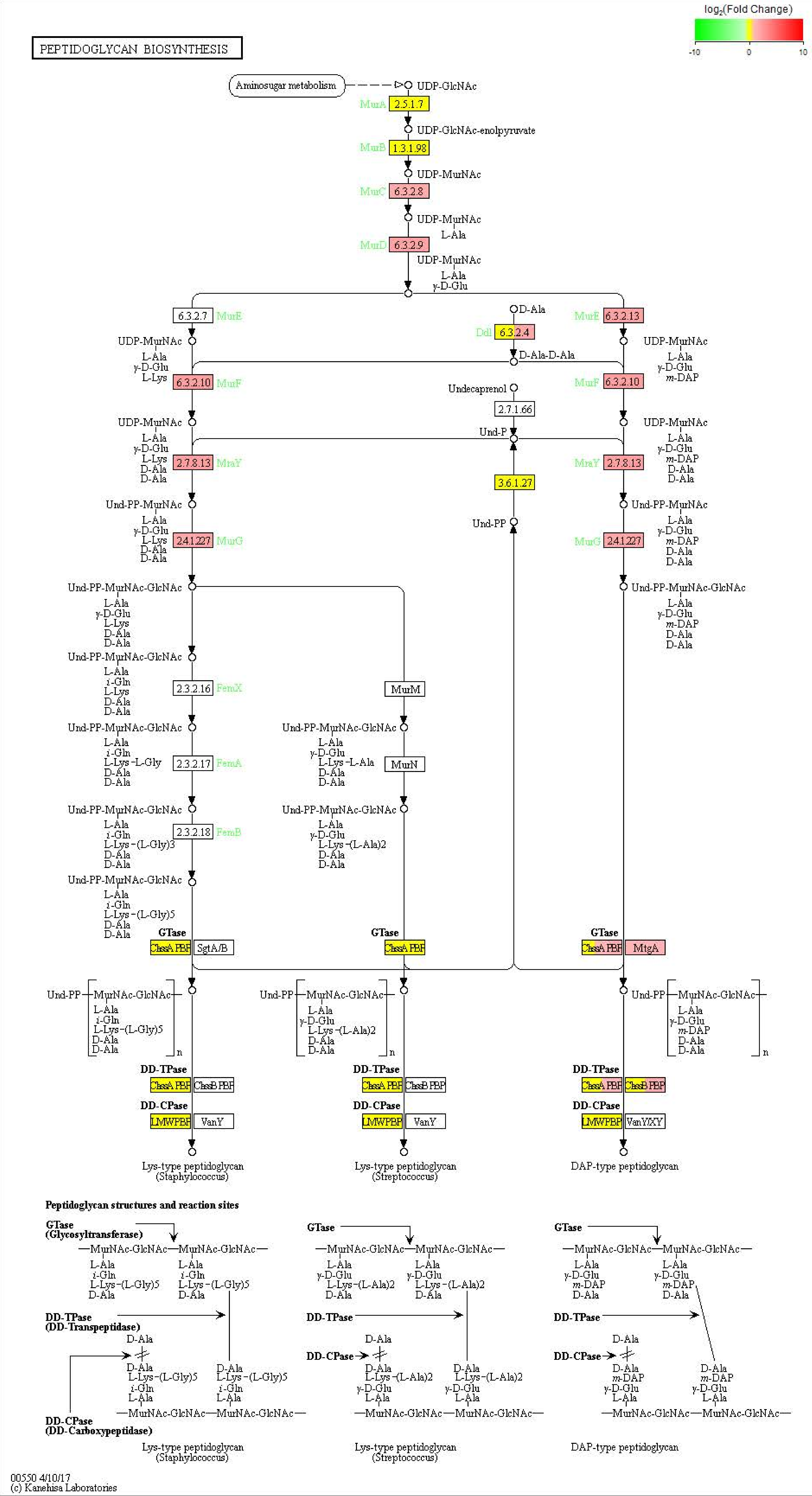

PEPTIDOGLYCAN BIOSYNTHESIS
log2(Fold Change)
-10
0
10
Aminosugar metabolism
UDP-GlcNAc
MurA
2.5.1.7
UDP-GlcNAc-enolpyruvate
MurB
1.3.1.98
UDP-MurNAc
MurC
6.3.2.8
UDP-MurNAc L-Ala
MurD
6.3.2.9
UDP-MurNAc L-Ala γ-D-Glu
6.3.2.7
MurE
D-Ala
Ddl
6.3.2.4
D-Ala-D-Ala
MurE
6.3.2.13
UDP-MurNAc L-Ala γ-D-Glu L-Lys
6.3.2.10
MurF
Undecaprenol
2.7.1.66
Und-P
MurF
6.3.2.10
UDP-MurNAc L-Ala γ-D-Glu m-DAP
UDP-MurNAc L-Ala γ-D-Glu L-Lys D-Ala D-Ala
2.7.8.13
MraY
3.6.1.27
MraY
2.7.8.13
UDP-MurNAc L-Ala γ-D-Glu m-DAP D-Ala D-Ala
Und-PP-MurNAc L-Ala γ-D-Glu L-Lys D-Ala D-Ala
2.4.1.227
MurG
Und-PP
MurG
2.4.1.227
Und-PP-MurNAc L-Ala γ-D-Glu m-DAP D-Ala D-Ala
Und-PP-MurNAc-GlcNAc L-Ala γ-D-Glu L-Lys D-Ala D-Ala
Und-PP-MurNAc-GlcNAc L-Ala γ-D-Glu m-DAP D-Ala D-Ala
Und-PP-MurNAc-GlcNAc L-Ala i-Gln L-Lys D-Ala D-Ala
2.3.2.16
FemX
MurM
Und-PP-MurNAc-GlcNAc L-Ala i-Gln L-Lys-L-Gly D-Ala D-Ala
2.3.2.17
FemA
Und-PP-MurNAc-GlcNAc L-Ala γ-D-Glu L-Lys-L-Ala D-Ala D-Ala
MurN
Und-PP-MurNAc-GlcNAc L-Ala i-Gln L-Lys-(L-Gly)3 D-Ala D-Ala
2.3.2.18
FemB
Und-PP-MurNAc-GlcNAc L-Ala γ-D-Glu L-Lys-(L-Ala)2 D-Ala D-Ala
Und-PP-MurNAc-GlcNAc L-Ala i-Gln L-Lys-(L-Gly)5 D-Ala D-Ala
GTase
ClassA PBP
SgtA/B
GTase
ClassA PBP
GTase
ClassA PBP
MtgA
Und-PP-[MurNAc-GlcNAc L-Ala i-Gln L-Lys-(L-Gly)5 D-Ala D-Ala]n
Und-PP-[MurNAc-GlcNAc L-Ala γ-D-Glu L-Lys-(L-Ala)2 D-Ala D-Ala]n
Und-PP-[MurNAc-GlcNAc L-Ala γ-D-Glu m-DAP D-Ala D-Ala]n
DD-TPase
ClassA PBP
ClassB PBP
DD-TPase
ClassA PBP
ClassB PBP
DD-TPase
ClassA PBP
ClassB PBP
DD-CPase
LMWPBP
VanY
DD-CPase
LMWPBP
VanY
DD-CPase
LMWPBP
VanY/X/Y
Lys-type peptidoglycan (Staphylococcus)
Lys-type peptidoglycan (Streptococcus)
DAP-type peptidoglycan
Peptidoglycan structures and reaction sites
GTase (Glycosyltransferase)
—MurNAc-GlcNAc—MurNAc-GlcNAc—
L-Ala i-Gln L-Lys-(L-Gly)5 D-Ala
L-Ala i-Gln L-Lys-(L-Gly)5 D-Ala
DD-TPase (DD-Transpeptidase)
D-Ala
D-Ala L-Lys-(L-Gly)5 i-Gln L-Ala
D-Ala L-Lys-(L-Gly)5 i-Gln L-Ala
—MurNAc-GlcNAc—MurNAc-GlcNAc—
DD-CPase (DD-Carboxypeptidase)
GTase
—MurNAc-GlcNAc—MurNAc-GlcNAc—
L-Ala γ-D-Glu L-Lys-(L-Ala)2 D-Ala
L-Ala γ-D-Glu L-Lys-(L-Ala)2 D-Ala
DD-TPase
DD-CPase
D-Ala
D-Ala L-Lys-(L-Ala)2 γ-D-Glu L-Ala
D-Ala L-Lys-(L-Ala)2 γ-D-Glu L-Ala
—MurNAc-GlcNAc—MurNAc-GlcNAc—
GTase
—MurNAc-GlcNAc—MurNAc-GlcNAc—
L-Ala γ-D-Glu m-DAP D-Ala
L-Ala γ-D-Glu m-DAP D-Ala
DD-TPase
DD-CPase
D-Ala
D-Ala m-DAP γ-D-Glu L-Ala
D-Ala m-DAP γ-D-Glu L-Ala
—MurNAc-GlcNAc—MurNAc-GlcNAc—
Lys-type peptidoglycan (Staphylococcus)
Lys-type peptidoglycan (Streptococcus)
DAP-type peptidoglycan
00550 4/10/17
(c) Kanehisa Laboratories

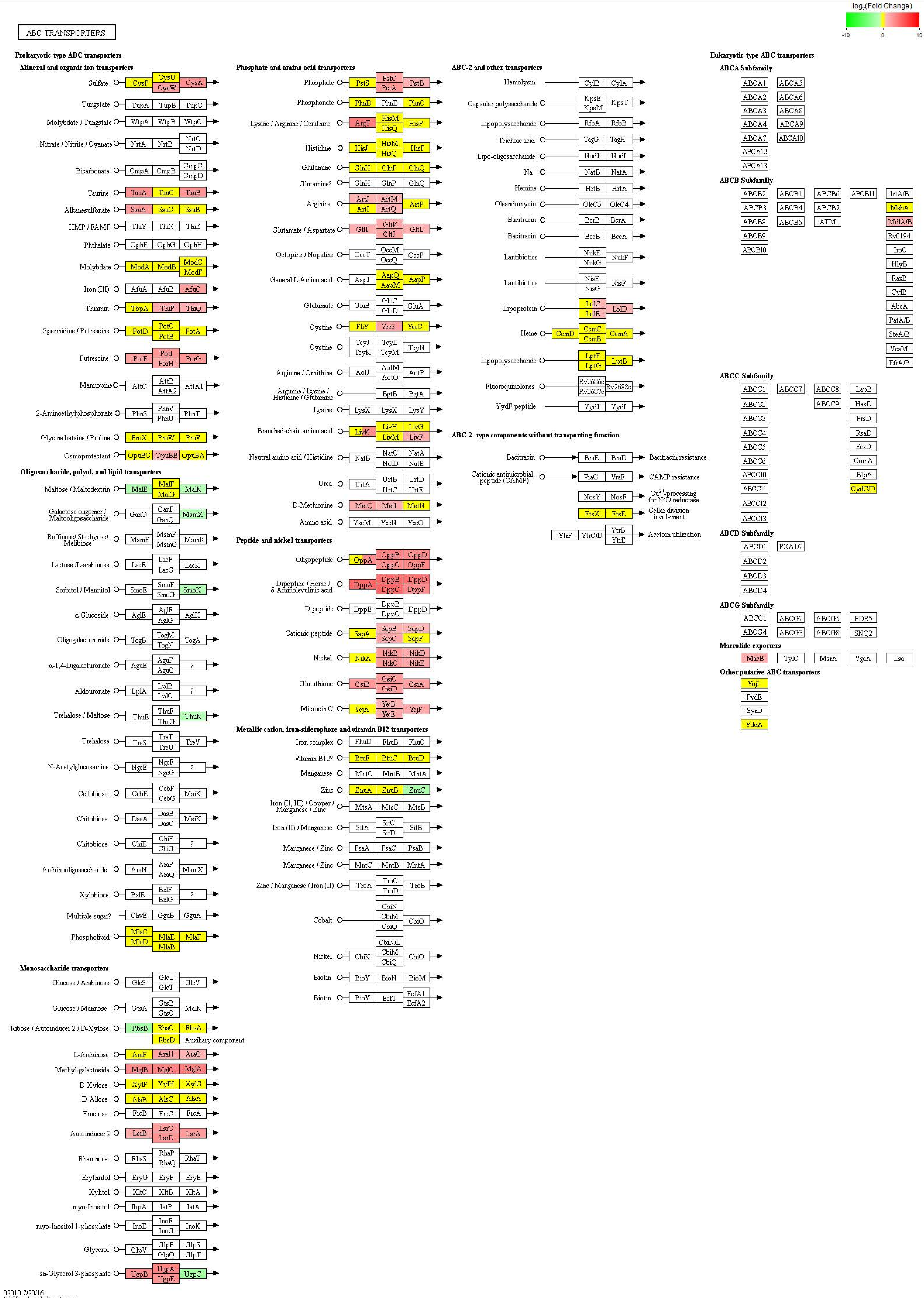

02010 7/20/16
(c) Kanehisa Laboratories

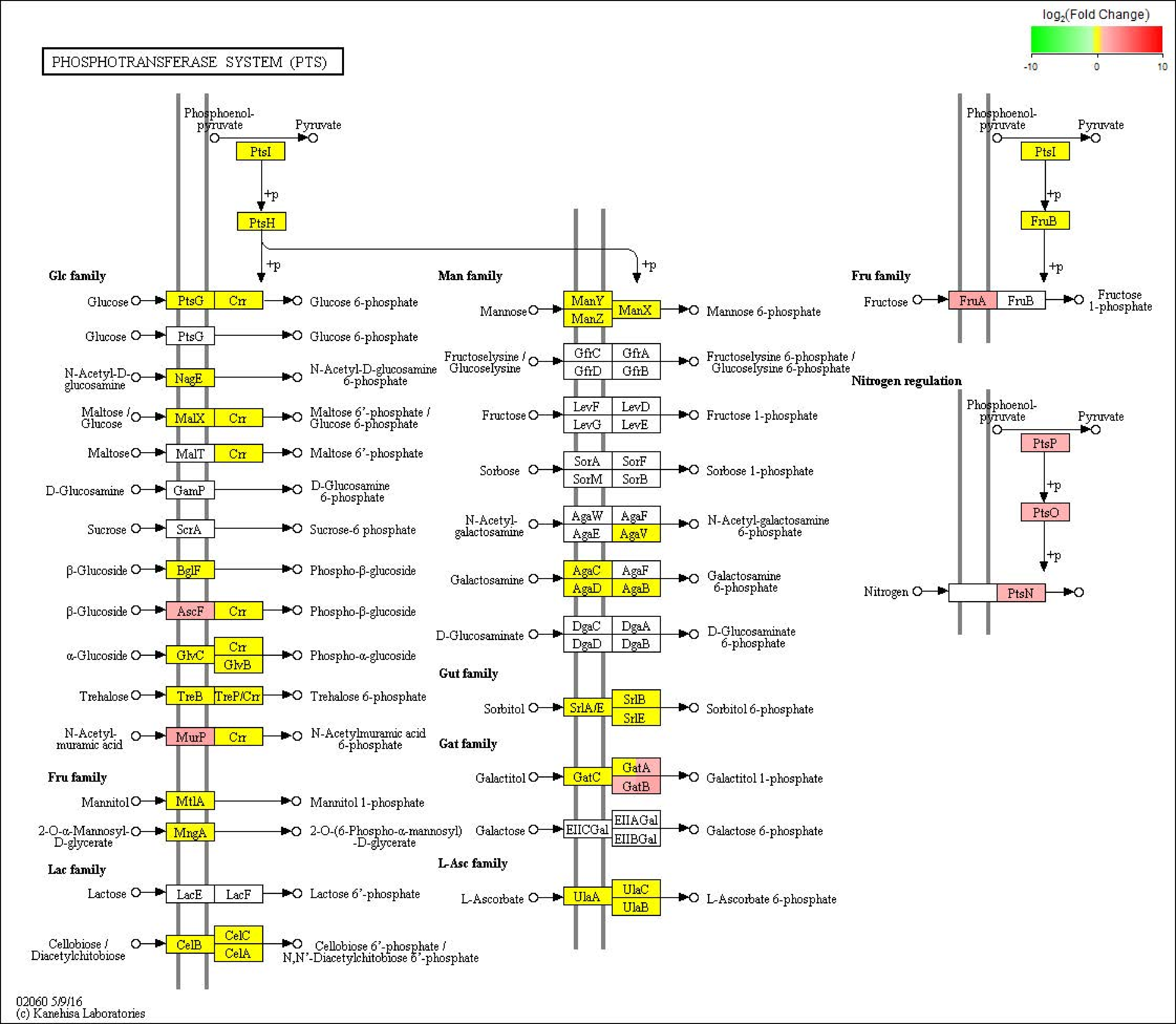

log2(Fold Change)
-10
0
10
PHOSPHOTRANSFERASE SYSTEM (PTS)
Phosphoenol-pyruvate
Pyruvate
PtsI
+p
PtsH
+p
Glc family
Glucose
PtsG
Crr
Glucose 6-phosphate
Glucose
PtsG
Glucose 6-phosphate
N-Acetyl-D-glucosamine
NagE
N-Acetyl-D-glucosamine 6-phosphate
Maltose / Glucose
MalX
Crr
Maltose 6'-phosphate / Glucose 6-phosphate
Maltose
MalT
Crr
Maltose 6'-phosphate
D-Glucosamine
GamP
D-Glucosamine 6-phosphate
Sucrose
ScrA
Sucrose-6 phosphate
β-Glucoside
BglF
Phospho-β-glucoside
β-Glucoside
AscF
Crr
Phospho-β-glucoside
α-Glucoside
GlvC
Crr
GlvB
Phospho-α-glucoside
Trehalose
TreB
TreP/Crr
Trehalose 6-phosphate
N-Acetyl-muramic acid
MurP
Crr
N-Acetylmuramic acid 6-phosphate
Fru family
Mannitol
MtlA
Mannitol 1-phosphate
2-O-α-Mannosyl-D-glycerate
MngA
2-O-(6-Phospho-α-mannosyl)-D-glycerate
Lac family
Lactose
LacE
LacF
Lactose 6'-phosphate
Cellobiose / Diacetylchitobiose
CelB
CelC
CelA
Cellobiose 6'-phosphate / N,N'-Diacetylchitobiose 6'-phosphate
Man family
Mannose
ManY
ManZ
ManX
Mannose 6-phosphate
Fructoselysine / Glucoselysine
GfrC
GfrA
GfrD
GfrB
Fructoselysine 6-phosphate / Glucoselysine 6-phosphate
Fructose
LevF
LevD
LevG
LevE
Fructose 1-phosphate
Sorbose
SorA
SorF
SorM
SorB
Sorbose 1-phosphate
N-Acetyl-galactosamine
AgaW
AgaF
AgaE
AgaV
N-Acetyl-galactosamine 6-phosphate
Galactosamine
AgaC
AgaF
AgaD
AgaB
Galactosamine 6-phosphate
D-Glucosaminate
DgaC
DgaA
DgaD
DgaB
D-Glucosaminate 6-phosphate
Gut family
Sorbitol
SrlA/E
SrlB
SrlE
Sorbitol 6-phosphate
Gat family
Galactitol
GatC
GatA
GatB
Galactitol 1-phosphate
Galactose
EIICGal
EIIAGal
EIIBGal
Galactose 6-phosphate
L-Asc family
L-Ascorbate
UlaA
UlaC
UlaB
L-Ascorbate 6-phosphate
Phosphoenol-pyruvate
Pyruvate
PtsI
+p
FruB
+p
Fru family
Fructose
FruA
FruB
Fructose 1-phosphate
Nitrogen regulation
Phosphoenol-pyruvate
Pyruvate
PtsP
+p
PtsO
+p
Nitrogen
PtsN
02060 5/9/16
(c) Kanehisa Laboratories

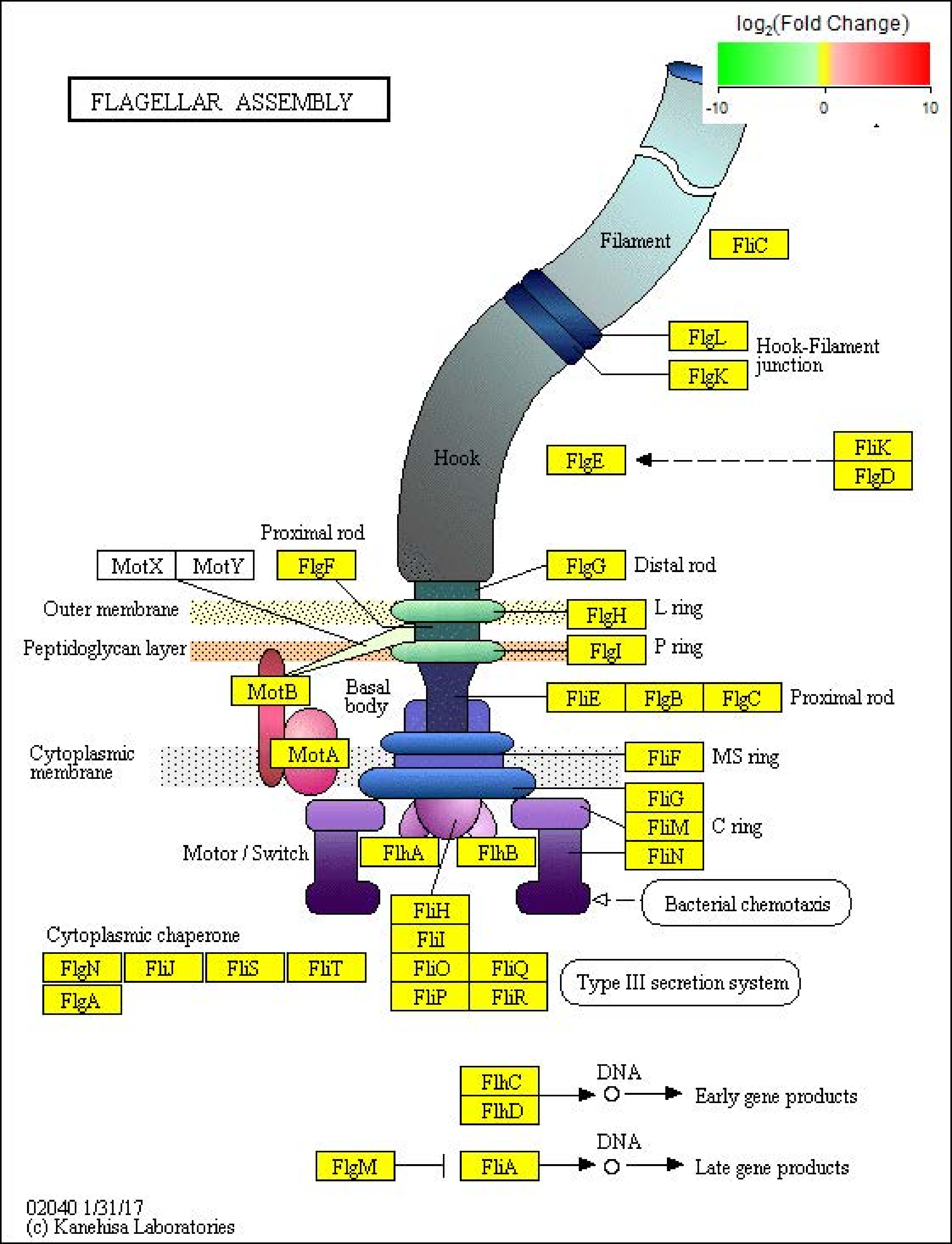
log2(Fold Change)
-10
0
10
FLAGELLAR ASSEMBLY
Filament
FliC
FlgL
FlgK
Hook-Filament junction
Hook
FlgE
FliK
FlgD
Proximal rod
MotX
MotY
FlgF
FlgG
Distal rod
Outer membrane
FlgH
L ring
Peptidoglycan layer
FlgI
P ring
MotB
Basal body
FliE
FlgB
FlgC
Proximal rod
Cytoplasmic membrane
MotA
FliF
MS ring
FliG
FliM
FliN
C ring
Motor / Switch
FlhA
FlhB
FliH
FliI
FliO
FliQ
FliP
FliR
Bacterial chemotaxis
Type III secretion system
Cytoplasmic chaperone
FlgN
FliJ
FliS
FliT
FlgA
FlhC
FlhD
DNA
Early gene products
FlgM
FliA
DNA
Late gene products
02040 1/31/17
(c) Kanehisa Laboratories

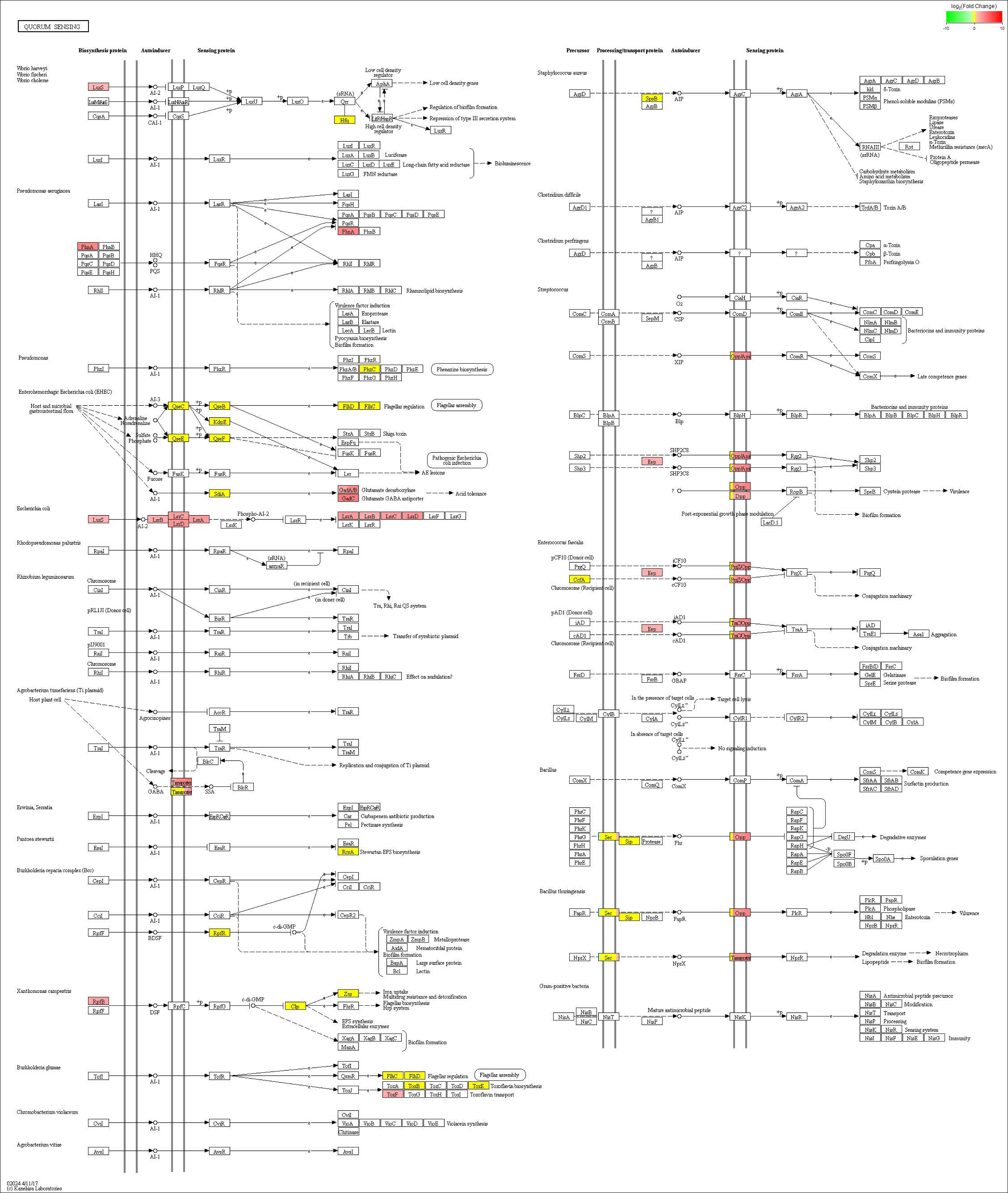

QUORUM SENSING
log2(Fold Change)
-10
0
10
Biosynthesis protein
Autoinducer
Sensing protein
Precursor
Processing/transport protein
Autoinducer
Sensing protein
Vibrio harveyi
Vibrio fischeri
Vibrio cholerae
LuxS
AI-2
LuxP
LuxQ
LuxM/AinS
AI-1
LuxN/AinR
CqsA
CAI-1
CqsS
LuxU
LuxO
(sRNA)
Qrr
Hfq
Low cell density regulator
AphA
Low cell density genes
LuxR/HapR
High cell density regulator
Regulation of biofilm formation
Repression of type III secretion system
LuxR
LuxI
LuxI
LuxR
LuxA
LuxB
Luciferase
LuxC
LuxD
LuxE
Long-chain fatty acid reductase
LuxG
FMN reductase
Bioluminescence
Pseudomonas aeruginosa
LasI
LasR
PqsH
PqsA
PqsB
PqsC
PqsD
PqsE
PqsR
PhnA
PhnB
HHQ
PQS
RhlI
RhlR
RhlA
RhlB
RhlC
Rhamnolipid biosynthesis
Virulence factor induction
LasA
Exoprotease
LasB
Elastase
LecA
LecB
Lectin
Pyocyanin biosynthesis
Biofilm formation
Pseudomonas
PhzI
PhzR
PhzA/B
PhzC
PhzD
PhzE
PhzF
PhzG
PhzH
Phenazine biosynthesis
Enterohemorrhagic Escherichia coli (EHEC)
Host and microbial gastrointestinal flora
AI-3
QseC
QseB
FlhD
FlhC
Flagellar regulation
Flagellar assembly
Adrenaline
Noradrenaline
KdpE
Sulfate
Phosphate
QseE
QseF
StxA
StxB
Shiga toxin
EspFu
FusK
FusR
Pathogenic Escherichia coli infection
Fucose
Ler
AE lesions
SdiA
GadA/B
Glutamate decarboxylase
GadC
Glutamate GABA antiporter
Acid tolerance
Escherichia coli
LsrB
LsrC
LsrD
LsrA
Phospho-AI-2
LsrK
LsrR
LsrF
LsrG
Rhodopseudomonas palustris
RpaI
RpaR
(sRNA)
asrpaR
Rhizobium leguminosarum
Chromosome
CinI
CinR
(in recipient cell)
(in doner cell)
Tra, Rhi, Rai QS system
pRL1JI (Donor cell)
BisR
TraR
TraI
Trb
Transfer of symbiotic plasmid
pIJ9001
RaiI
RaiR
RhiI
RhiR
RhiA
RhiB
RhiC
Effect on nodulation?
Agrobacterium tumefaciens (Ti plasmid)
Host plant cell
Agrocinopines
AccR
TraM
BlcC
BlcR
Cleavage
GABA
Transporter
SSA
Replication and conjugation of Ti plasmid
Erwinia, Serratia
ExpI
ExpR/CarR
Car
Carbapenem antibiotic production
Pel
Pectinase synthesis
Pantoea stewartii
EsaI
EsaR
RcsA
Stewartan EPS biosynthesis
Burkholderia cepacia complex (Bcc)
CepI
CepR
CciI
CciR
CepR2
RpfF
BDSF
RpfR
c-di-GMP
ZmpA
ZmpB
Metalloprotease
AidA
Nematocidal protein
BapA
Large surface protein
BclA
Lectin
Xanthomonas campestris
RpfB
DSF
RpfC
RpfG
Clp
Zur
Iron uptake
Multidrug resistance and detoxification
FhrR
Flagellar biosynthesis
Hrp system
EPS synthesis
Extracellular enzymes
XagA
XagB
XagC
ManA
Burkholderia glumae
TofI
TofR
QsmR
Toxoflavin biosynthesis
Toxoflavin transport
ToxA
ToxB
ToxC
ToxD
ToxE
ToxF
ToxG
ToxH
ToxI
ToxJ
Chromobacterium violaceum
CviI
CviR
VioA
VioB
VioC
VioD
VioE
Violacein synthesis
Chitinase
Agrobacterium vitiae
AvsI
AvsR
Staphylococcus aureus
AgrD
SpsB
AgrB
AIP
AgrC
AgrA
hld
δ-Toxin
PSMα
PSMβ
Phenol-soluble modulins (PSMs)
RNAIII
(srRNA)
Rot
Exoproteases
Lipase
Urease
Enterotoxin
Leukocidins
α-Toxin
Methicillin resistance (mecA)
Protein A
Oligopeptide permease
Carbohydrate metabolism
Amino acid metabolism
Staphyloxanthin biosynthesis
Clostridium difficile
AgrD1
AgrB1
AgrC2
AgrA2
TcdA/B
Toxin A/B
Clostridium perfringens
Cpa
α-Toxin
Cpb
β-Toxin
PfoA
Perfringolysin O
Streptococcus
O2
CiaH
CiaR
ComC
ComA
ComB
CSP
ComD
ComE
SepM
NlmA
NlmB
NlmC
NlmD
CipI
Bacteriocins and immunity proteins
ComS
XIP
Opp/Ami
ComR
ComX
Late competence genes
BlpC
BlpA
BlpB
Blp
BlpH
BlpR
BlpI
BlpK
Shp2
Shp3
Eep
SHP2C8
SHP3C8
Rgg2
Rgg3
Opp
Dpp
RopB
SpeB
Cysteine protease
Virulence
Post-exponential growth phase modulation
LacD.1
Biofilm formation
Enterococcus faecalis
pCF10 (Donor cell)
PrgQ
iCF10
PrgZ/Opp
PrgX
CcfA
cCF10
Chromosome (Recipient cell)
Conjugation machinary
pAD1 (Donor cell)
iAD
iAD1
TraC/Opp
TraA
cAD1
TraE1
AsaI
Aggregation
FsrD
FsrB
GBAP
FsrC
FsrA
FsrB/D
GelE
Gelatinase
SprE
Serine protease
CylLL
CylLS
CylM
CylB
CylA
In the presence of target cells
Target cell lysis
CylR1
CylR2
In absence of target cells
No signaling induction
Bacillus
ComX
ComQ
ComP
ComK
Competence gene expression
SfrAA
SfrAB
SfrAC
SfrAD
Surfactin production
PhrC
PhrF
PhrK
PhrG
PhrH
PhrA
PhrE
Sec
Sip
Protease
Phr
RapC
RapF
RapK
RapG
RapH
RapA
RapE
RapB
DegU
Degradative enzymes
Spo0F
Spo0B
Spo0A
Sporulation genes
Bacillus thuringiensis
PapR
NprB
PlcR
PlcA
Phospholipase
Hbl
Nhe
Enterotoxin
NprB
Vilurence
NprX
NprR
Degradation enzyme
Necrotrophism
Lipopeptide
Gram-positive bacteria
NisA
NisB
NisC
NisT
Mature antimicrobial peptide
NisP
NisK
NisR
Antimicrobial peptide precursor
Modification
Transport
Processing
Sensing system
NisI
NisF
NisE
NisG
Immunity
02024 4/11/17
(c) Kanehisa Laboratories

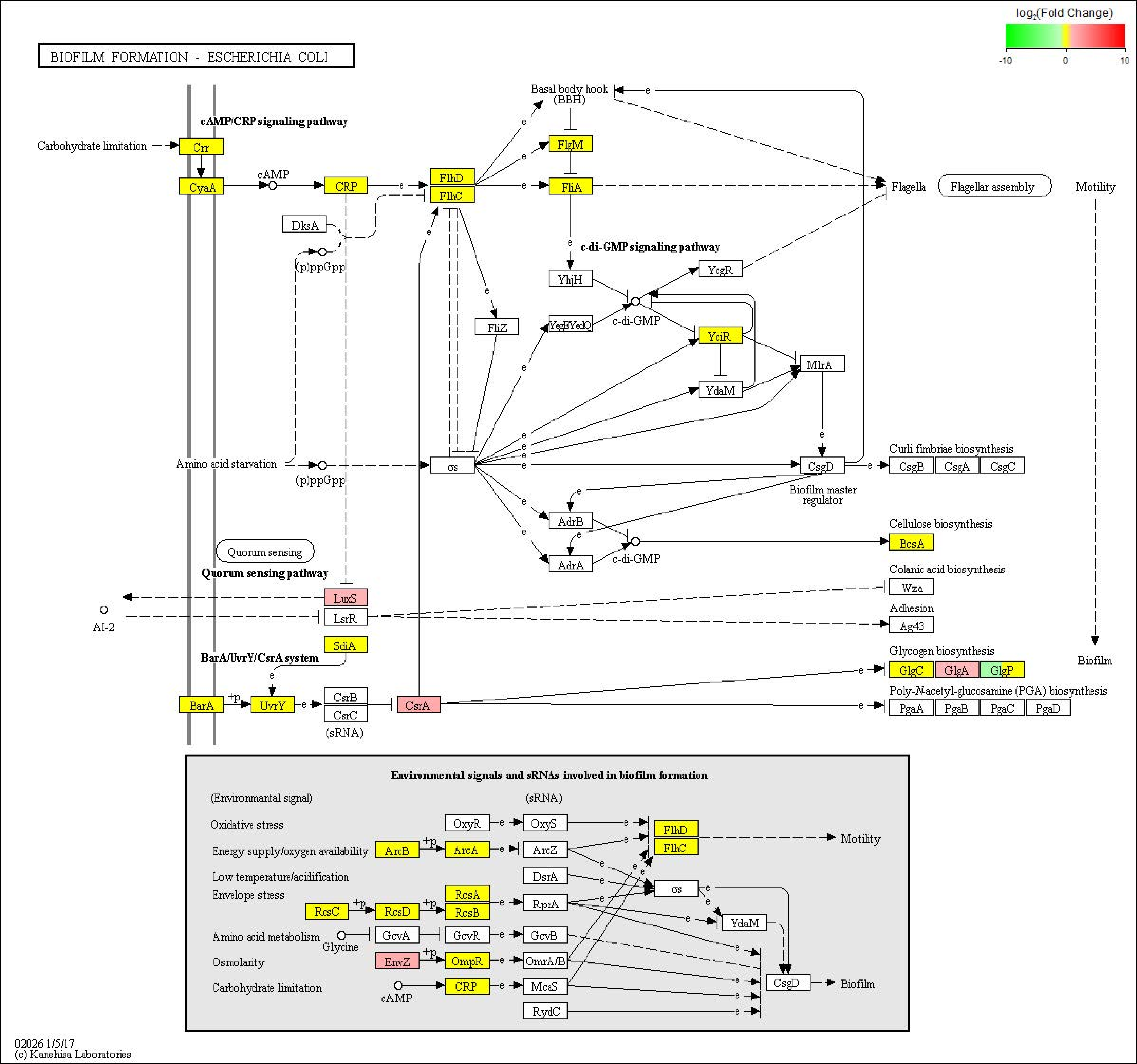
BIOFILM FORMATION - ESCHERICHIA COLI
log2(Fold Change)
-10
0
10
cAMP/CRP signaling pathway
Carbohydrate limitation
Crr
CyaA
cAMP
CRP
DksA
(p)ppGpp
FlhD
FlhC
Basal body hook (BBH)
FlgM
FliA
Flagella
Flagellar assembly
Motility
c-di-GMP signaling pathway
YhjH
YcgR
YegE/YedQ
c-di-GMP
YciR
YdaM
MlrA
FliZ
Amino acid starvation
σS
CsgD
Biofilm master regulator
Curli fimbriae biosynthesis
CsgB
CsgA
CsgC
AdrB
AdrA
Cellulose biosynthesis
BcsA
Quorum sensing
Quorum sensing pathway
LuxS
LsrR
AI-2
Colanic acid biosynthesis
Wza
Adhesion
Ag43
SdiA
BarA/UvrY/CsrA system
BarA
+p
UvrY
CsrB
CsrC
(sRNA)
CsrA
Glycogen biosynthesis
GlgC
GlgA
GlgP
Biofilm
Poly-N-acetyl-glucosamine (PGA) biosynthesis
PgaA
PgaB
PgaC
PgaD
Environmental signals and sRNAs involved in biofilm formation
(Environmantal signal)
(sRNA)
Oxidative stress
OxyR
OxyS
Energy supply/oxygen availability
ArcB
ArcA
ArcZ
Low temperature/acidification
DsrA
Envelope stress
RcsC
RcsD
RcsA
RcsB
RprA
Amino acid metabolism
Glycine
GcvA
GcvR
GcvB
Osmolarity
EnvZ
OmpR
OmrA/B
Carbohydrate limitation
cAMP
CRP
McaS
RydC
FlhD
FlhC
Motility
σS
YdaM
CsgD
Biofilm
02026 1/5/17
(c) Kanehisa Laboratories

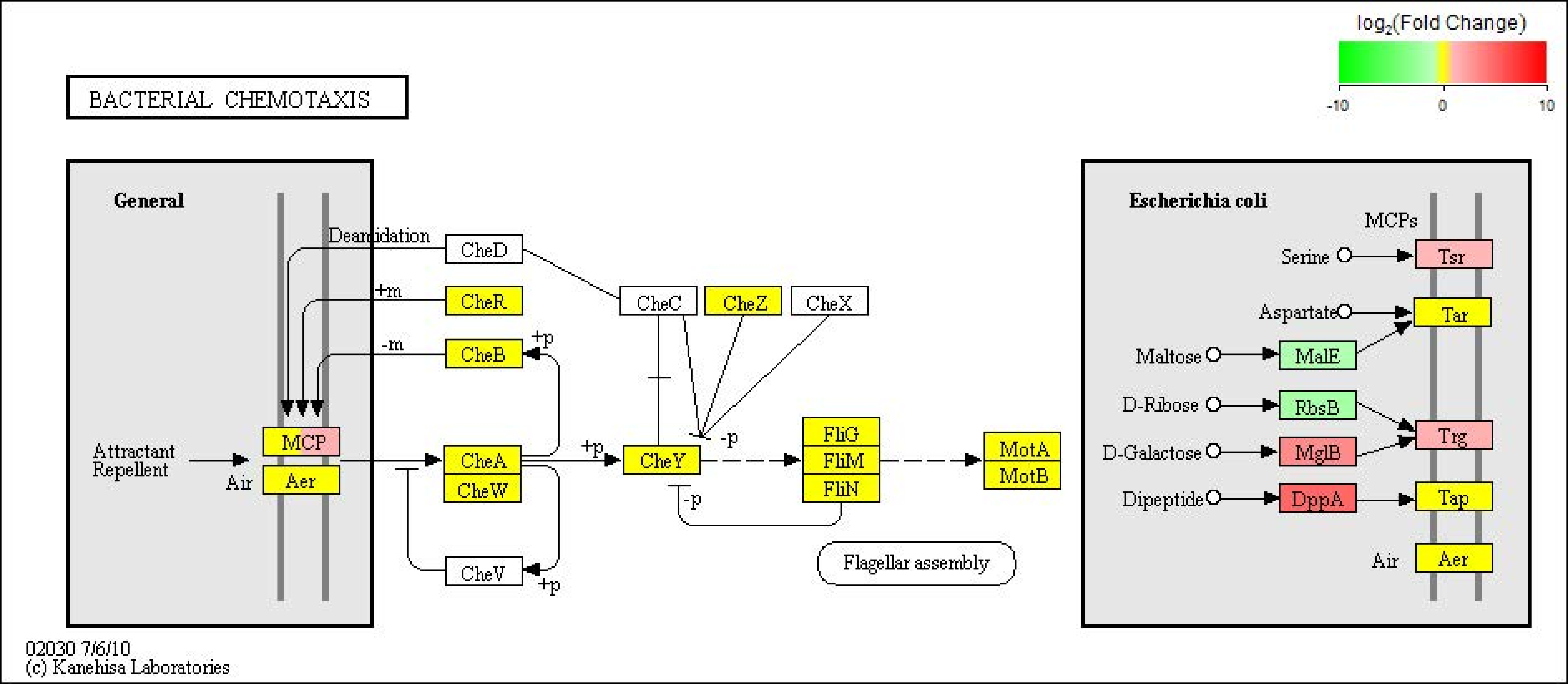

log2(Fold Change)
-10
0
10
BACTERIAL CHEMOTAXIS
General
Deamidation
CheD
+m
CheR
-m
CheB
+p
Attractant
Repellent
Air
MCP
Aer
CheA
CheW
CheV
+p
+p
CheC
CheZ
CheX
CheY
-p
-p
FliG
FliM
FliN
MotA
MotB
Flagellar assembly
Escherichia coli
MCPs
Serine
Tsr
Aspartate
Tar
Maltose
MalE
D-Ribose
RbsB
Trg
D-Galactose
MglB
Dipeptide
DppA
Tap
Air
Aer
02030 7/6/10
(c) Kanehisa Laboratories
