# Supplementary material for: Resistance characterization and transcriptomic analysis of imipenem-induced drug resistance in Escherichia coli
Source: PeerJ. 2024 Nov 29;12:e18572. doi: 10.7717/peerj.18572 (PMC11610472; doi:10.7717/peerj.18572)
Supplement: Table S2 [file peerj-12-18572-s008.docx]

Table S2 Penicillin-binding protein gene and primer sequences

| Genes | Primers | Product Size (bp) |
| --- | --- | --- |
| PBP1a-*mrcA* | F：CCTTATCACCCGTCACTCT  R：TTTCACGATTTTGTTAGCC | 3107 |
| PBP1b-*mrcB* | F：TGGTGCTGGAGTTGCTTT  R：CGGTAACGGTGATAGTGTCTT | 3262 |
| PBP2-*mrdA* | F：CGCTCCATCATGCCAATATCCT  R：CACCACCAACCATCCTTATCACC | 2117 |
| PBP3-*ftsI* | F：TGGAGCGAGATGCTTTAGACA  R：CGTCCGCCTGATGACCTACT | 2071 |
| PBP4-*dacB* | F：ACAAGTCCCAGGTCAGCTACA  R：GACGAAGACGACGAAGAAGG | 1806 |
| PBP5-*dacA* | F：TACAGAACCATGCGGACAAT  R：TACCAACAGCAACTGGGACA | 1902 |
| PBP6a-*dacC* | F：CCACAGGCTGAACAGGACTC  R：GCAGCGCATTAAGTTGATGTA | 1496 |
| PBP6b-*dacD* | F：AAATCATCACCGACTACACGA  R：CTGCCAGAGTATCAAATATCACC | 1793 |
| PBP7-*pbpG* | F：ACTGGCAGGAAGTGGAGT  R：TTTGCTCGTTATGATGGC | 1079 |
