# Supplementary material for: Resistance characterization and transcriptomic analysis of imipenem-induced drug resistance in Escherichia coli
Source: PeerJ. 2024 Nov 29;12:e18572. doi: 10.7717/peerj.18572 (PMC11610472; doi:10.7717/peerj.18572)
Supplement: Table S3 [file peerj-12-18572-s009.docx]

Table S3 Carbapenem hydrolase gene and primer sequences

| Genes | Primers | Product Size (bp) |
| --- | --- | --- |
| *bla* KPC | F: CGTCTAGTTCTGCTGTCTTG  R: CTTGTCATCCTTGTTAGGCG | 798 |
| *bla* SME | F: AGATAGTAAATTTTATAG  R: CTCTAACGCTAAATAG | 1138 |
| *bla* IMI | F: ATAGCCATCCTTGTTTAGCTC  R: TCTGCGATTACTTTATCCTC | 818 |
| *bla* GES | F: GTTTTGCAATGTGCTCAACG  R: TGCCATAGCAATAGGCGTAG | 371 |
| *bla* IMP | F: CATGGTTTGGTGGTTCTTGT  R: GTACGTTTCAAGAGTGATGC | 528 |
| *bla* VIM | F: GTTTGGTCGCATATCGCAAC  R: CTACTCGGCGACTGAGCGAT | 645 |
| *bla* SIM | F: TACAAGGGATTCGGCATCG  R: TAATGGCCTGTTCCCATGTG | 570 |
| *bla* GIM | F: TCGACACACCTTGGTCTGAA  R: AACTTCCAACTTTGCCATGC | 477 |
| *bla* NDM | F: GGTTTGGCGATCTGGTTTTC  R: CGGAATGGCTCATCACGATC | 621 |
| *bla* OXA48 | F: GCTTGATCGCCCTCGATT  R: GATTTGCTCCGTGGCCGAAA | 281 |
