# Supplementary material for: Resistance characterization and transcriptomic analysis of imipenem-induced drug resistance in Escherichia coli
Source: PeerJ. 2024 Nov 29;12:e18572. doi: 10.7717/peerj.18572 (PMC11610472; doi:10.7717/peerj.18572)
Supplement: Table S4 [file peerj-12-18572-s010.docx]

mdtC knockout strain sequencing fragments

ATTGTGAGCGGATAACAATTTGTGGAATCCCGGGAGAGCTCCTCGGCATTCTGTACGAGAGCTTTATTCACCCGATCACCATTCTCTCGACGCTACCCACCGCAGGGGTTGGCGCACTGCTGGCGTTGCTGATTGCTGGTAGCGAACTGGATGTGATTGCGATTATCGGCATTATTTTGCTGATCGGTATCGTGAAGAAGAACGCCATCATGATGATCGACTTCGCGCTGGCTGCTGAGCGCGAGCAAGGCATGTCGCCGCGCGAGGCAATCTACCAGGCTTGTCTGTTGCGTTTTCGTCCGATCCTGATGACCACTCTGGCGGCTCTGCTTGGCGCGCTGCCGCTGATGTTGAGTACCGGGGTCGGCGCGGAACTGCGTCGTCCGTTAGGTATCGGCATGGTCGGCGGTCTGATTGTCAGCCAGGTGCTGACGCTGTTTACCACGCCGGTGATTTATTTGCTGTTCGACCGCCTGGCATTGTGGACCAAAAGCCGCTTTGCCCGTCATGAAGAGGAGGCGTAAGTGTCGCTGGACGACGTACGCACCGCCGTCAGCAATGCCAACGTGCGTAAACCGCAGGGCGCGCTGGAAGATGGCACTCACCGCTGGCAGATCCAGACCAATGATGAGCTAAAAACCGCCGCTGAATATCAGCCGTTGATTATTCACTACAACAACGGCGGCGCGGTTCGTCTGGGCGATGTGGCGACGGTGACCGACTCAGTGCAGGATGTGCGCAACGCCGGGATGACCAACGCCAAACCGGCTATTTTACTGATGATCCGCAAACTGCCGGAAGCCAATATTATCCAGACGGTTGACAGCATCCGGGCAAAATTACCGGAGTTGCAGGAAACCATTCCGGCGGCGATTGATCTGCAAATTGCCCAGGATCGCTCCCCCACCATTCGCGCCTCGCTGGAAGAAGTCGAGCAAACGCTGATTATCTCGGTGGCGCTGGTGATTCTGGTGGTGTTTTTATTCCTGCGCTCGGGTCGCGCCACTATTATTCCCGCCGCTAGCGCTGATGTCCGGCGGTGCTTTTGCC

mdtD knockout strain sequencing fragments

TTGAGTGACACAGGAACACTTAACGGCTGACATGGGAATTCCACATGTGGAATTCCACATGTGGAATTGTGAGCGGATAACAATTTGTGGAATCCCGGGAGAGCTCGTGCTGGGTATCCTTTACGAGAGTTACGTACATCCGCTGACGATTCTCTCCACCCTGCCCTCGGCGGGCGTTGGAGCGCTGTTGGCGCTGGAGCTGTTCAATGCCCCGTTCAGCCTAATCGCCCTGATAGGGATCATGCTATTAATCGGCATCGTGAAGAAAAACGCCATTATGATGGTCGATTTTGCGCTTGAAGCCCAACGGCACGGTAACCTGACGCCGCAGGAAGCTATTTTCCAGGCCTGTCTGCTGCGTTTTCGCCCGATTATGATGACTACCCTGGCGGCGCTGTTTGGTGCGCTGCCGCTGGTATTGTCGGGCGGCGACGGCTCGGAGCTGCGGCAACCCCTGGGGATCACCATTGTCGGCGGACTGGTAATGAGCCAGCTCCTTACGCTGTATACCACGCCGGTGGTGTATCTCTTTTTCGACCGTCTGCGGCTGCGTTTTTCGCGTAAACCTAAACAAACGGTAACCGAGTAACTGATGATGATCCCGATGGTGCTTGGCAGCATGGGAATGAAGCGAATTGTGGTACAGGTGGTGAATCGCTTTGGTTATCGTCGGGTACTGGTAGCGACCACGCTGGGTCTGTCGCTGGTCACCCTGTTGTTTATGACTACCGCCCTGCTGGGCTGGTACTACGTTTTGCCGTTCGTCCTGTTTTTACAAGGGATGGTCAACTCGACGCGTTTCTCCTCCATGAACACCCTGACGCTGAAAGATCTCCCGGACAATCTGGCGAGCAGCGGCAACAGCCTGCTGTCGATGATTATGCAATTGTCGATGAGTATCGGCGTCACTATCGCCGGGCTGTTGCTGGGACTTTTTGGTTCACAGCATGTCAGCGTCGACAGCGGCACCACACAAACCGTCTTTATGTACACCTGGCTTAGCATGGCGTTGATCATCGCCCTTCCGGCGTTCATCTTTGCCAGAGTGCCGAACGATACGCATCAAAATGTAGCTAGCGCTGATGTCCGGCGGTGCTTTTGCCGTTACGCACCACCCCGTCAGTAGCTGAACAGGAGGGACAGCTGATAGAAACAGAAGCCACTGGAGCACCTCAAAA

macB knockout strain sequencing fragments

TTAACGGCTGACATGGGAATTCCACATGTGGAATTCCACATGTGGAATTGTGAGCGGATAACAATTTGTGGAATCCCGGGAGAGCTCAGCACCGAACATTCTGACGCTGGCAGATATGAGCGCCATGCTGGTAAAAGCGCAGGTTTCTGAAGCGGATGTAATCCACCTGAAGCCGGGGCAAAAAGCCTGGTTTACGGTGCTTGGCGATCCACTGACGCGCTACGAGGGGCAAATCAAGGATGTACTACCGACGCCGGAAAAGGTTAACGACGCTATTTTCTATTACGCCCGTTTTGAAGTCCCCAACCCCAATGGTTTGCTGCGGCTGGATATGACTGCGCAAGTGCATATTCAGCTCACCGATGTGAAAAATGTGCTGACGATCCCTCTGTCGGCGTTAGGCGATCCGGTTGGCGATAATCGTTATAAAGTCAAATTGTTGCGTAATGGTGAAACACGCGAGCGTGAAGTGACGATTGGCGCACGTAACGATACCGATGTTGAGATTGTCAAAGGGCTTGAAGCGGGCGATGAAGTGGTGATTGGTGAGGCCAAACCAGGAGCTGCACACTGGCGGATATTCGTTCTATTGGTACGAATACTATTGATGTCTATCCCGGGAAAGATTTTGGCGATGACGATCCGCAATATCAGCAGGCGCTGAAGTACGACGACTTAATCGCCATCCAAAAACAACCGTGGGTCGCCTCAGCCACACCTGCCGTCTCGCAAAACCTGCGCCTGCGTTATAACAATGTTGATGTTGCTGCCAGTGCCAATGGCGTGAGCGGCGATTATTTTAATGTCTATGGCATGACCTTCAGTGAAGGAAACACCTTTAATCAGGAGCAGCTGAACGGTCGTGCGCAGGTCGTGGTTCTCGACAGTAATACTCGCCGCCAGCTTTTCCCCCATAAAGCAGATGTGGTTGGCGAGGTGATTCTGGTCGGCAATATGCCCGCCAGAGTCATTGGTGTGGCGGAAGAAAAACAGTCGATGTTTGGTAGCAGTAAAGTGCTGCGTGTCTGGCTACCTTACAGCACGATGTCCGGGGCTAGCGCTGATGTCCGGCGGTGCTTTTGCCGTTACGCACCACCCCGTC

mdtF knockout strain sequencing fragments

TTGAGTGACACAGGAACACTTAACGGCTGACATGGGAATTCCACATGTGGAATTCCACATGTGGAATTGTGAGCGGATAACAATTTGTGGAATCCCGGGAGAGCTCGCTATGCCTTCATTACCTGAAGCAGTGCAGCAGCAGGGGATTAGCGTCGATAAGTCGAGCAGTAATATCCTGATGGTAGCGGCGTTTATTTCTGATAACGGCAGCCTCAACCAGTACGATATCGCGGACTATGTAGCGTCTAATATCAAAGACCCGCTAAGCCGTACCGCGGGCGTTGGTAGCGTACAACTCTTTGGTTCCGAGTATGCCATGCGTATCTGGCTGGACCCGCAAAAACTCAATAAATATAACCTGGTACCTTCCGATGTTATTTCCCAGATTAAGGTGCAAAACAACCAGATTTCCGGTGGTCAACTGGGTGGCATGCCACAGGCGGCAGACCAGCAGCTAAACGCCTCGATCATTGTGCAGACGCGTCTGCAAACGCCGGAAGAATTTGGCAAAATCCTGTTGAAAGTTCAGCAAGATGGTTCGCAAGTGGAACGTGTCGGTGAGGAAAACTCGGTTACCGCGATCATTCAGCGGGCAATGATTGCGTTAAGCAGTATCAATAAAGCCGTCGTCTTCCCGTTCAACTTACCCGCGGTGGCTGAACTGGGTACCGCGTCAGGTTTTGATATGGAACTGCTGGACAACGGTAACCTGGGGCACGAAAAACTAACCCAGGCGCGAAACGAGCTGTTATCACTGGCAGCGCAATCACCGAATCAGGTCACCGGGGTACGCCCGAACGGCCTGGAAGATACGCCGATGTTCAAAGTGAACGTCAACGCTGCGAAAGCTGAAGCGATGGGCGTGGCGCTGTCTGATATCAACCAGACAATTTCCACCGCCTTCGGCAGCAGCTACGTGAACGACTTCCTCAACCAGGGGCGGGTGAAAAAAGTGTATGTCCAGGCAGGCACGCCGTTCCGTATGTTGCCGGATAACATCAACCAATGGTATGTACGCAACGCCTCTGGCACGATGGCACCGCTTTCTGCCTACTCGTCTACCGAATGGGCTAGCGCTGATGTCCGGCGGTGCTTTTGCCGTTACGCACCACCCC
