# Supplementary material for: Resistance characterization and transcriptomic analysis of imipenem-induced drug resistance in Escherichia coli
Source: PeerJ. 2024 Nov 29;12:e18572. doi: 10.7717/peerj.18572 (PMC11610472; doi:10.7717/peerj.18572)
Supplement: Table S5 [file peerj-12-18572-s011.docx]

*mdtC* gene sequencing fragment of K-12 strain

CTCGGCATTCTGTACGAGAGCTTTATTCACCCGATCACCATTCTCTCGACGCTACCCACCGCAGGGGTTGGCGCACTGCTGGCGTTGCTGATTGCTGGTAGCGAACTGGATGTGATTGCGATTATCGGCATTATTTTGCTGATCGGTATCGTGAAGAAGAACGCCATCATGATGATCGACTTCGCGCTGGCTGCTGAGCGCGAGCAAGGCATGTCGCCGCGCGAGGCAATCTACCAGGCTTGTCTGTTGCGTTTTCGTCCGATCCTGATGACCACTCTGGCGGCTCTGCTTGGCGCGCTGCCGCTGATGTTGAGTACCGGGGTCGGCGCGGAACTGCGTCGTCCGTTAGGTATCGGCATGGTCGGCGGTCTGATTGTCAGCCAGGTGCTGACGCTGTTTACCACGCCGGTGATTTATTTGCTGTTCGACCGCCTGGCATTGTGGACCAAAAGCCGCTTTGCCCGTCATGAAGAGGAGGCGTAAGTGAAGTTTTTTGCCCTCTTCATTTACCGCCCGGTGGCGACGATTTTACTGTCGGTTGCCATTACCCTGTGCGGCATACTGGGCTTCCGTATGCTGCCGGTCGCCCCGCTGCCGCAGGTCGATTTTCCGGTGATTATCGTCAGCGCCTCGCTGCCCGGTGCGTCACCAGAAACAATGGCGTCTTCCGTTGCCACGCCGCTGGAGCGCTCACTTGGGCGCATTGCCGGAGTCAGTGAAATGACCTCCAGCAGTTCGCTCGGCAGCACGCGTATTATTTTGCAGTTTGATTTTGACCGGGATATCAACGGCGCAGCGCGTGATGTGCAGGCGGCGATCAACGCTGCACAAAGTTTGCTGCCCAGTGGGATGCCCAGCCGCCCGACCTATCGCAAAGCGAACCCGTCGGATGCGCCAATTATGATCCTCACGCTGACGTCCGATACTTATTCGCAGGGTGAACTGTACGATTTCGCCTCGACGCAGCTGGCTCCGACGATTTCGCAAATCGACGGTGTTGGTGATGTCGATGTCGGAGGCAGCTCACTGCCCGCCGTACGCGTCGGGCTGAATCCGCAGGCGCTGTTTAATCAGGGCGTGTCGCTGGACGACGTACGCACCGCCGTCAGCAATGCCAACGTGCGTAAACCGCAGGGCGCGCTGGAAGATGGCACTCACCGCTGGCAGATCCAGACCAATGATGAGCTAAAAACCGCCGCTGAATATCAGCCGTTGATTATTCACTACAACAACGGCGGCGCGGTTCGTCTGGGCGATGTGGCGACGGTGACCGACTCAGTGCAGGATGTGCGCAACGCCGGGATGACCAACGCCAAACCGGCTATTTTACTGATGATCCGCAAACTGCCGGAAGCCAATATTATCCAGACGGTTGACAGCATCCGGGCAAAATTACCGGAGTTGCAGGAAACCATTCCGGCGGCGATTGATCTGCAAATTGCCCAGGATCGCTCCCCCACCATTCGCGCCTCGCTGGAAGAAGTCGAGCAAACGCTGATTATCTCGGTGGCGCTGGTGATTCTGGTGGTGTTTTTATTCCTGCGCTCGGGTCGCGCCACTATTATTCCCGCCG

*mdtD* gene sequencing fragment of K-12 strain

TCGTGCTGGGTATCCTTTACGAGAGTTACGTACATCCGCTGACGATTCTCTCCACCCTGCCCTCGGCGGGCGTTGGAGCGCTGTTGGCGCTGGAGCTGTTCAATGCCCCGTTCAGCCTAATCGCCCTGATAGGGATCATGCTATTAATCGGCATCGTGAAGAAAAACGCCATTATGATGGTCGATTTTGCGCTTGAAGCCCAACGGCACGGTAACCTGACGCCGCAGGAAGCTATTTTCCAGGCCTGTCTGCTGCGTTTTCGCCCGATTATGATGACTACCCTGGCGGCGCTGTTTGGTGCGCTGCCGCTGGTATTGTCGGGCGGCGACGGCTCGGAGCTGCGGCAACCCCTGGGGATCACCATTGTCGGCGGACTGGTAATGAGCCAGCTCCTTACGCTGTATACCACGCCGGTGGTGTATCTCTTTTTCGACCGTCTGCGGCTGCGTTTTTCGCGTAAACCTAAACAAACGGTAACCGAGTAAATGACAGATCTTCCCGACAGCACCCGTTGGCAATTGTGGATTGTGGCTTTCGGCTTCTTTATGCAGTCGCTGGACACCACCATCGTAAACACCGCCCTTCCCTCAATGGCGCAAAGCCTCGGGGAAAGTCCGTTGCATATGCACATGGTCATTGTCTCTTATGTGCTGACCGTGGCGGTGATGCTGCCCGCCAGCGGCTGGCTGGCGGACAAAGTCGGCGTGCGCAATATTTTCTTTACCGCCATCGTGCTGTTTACTCTCGGTTCACTGTTTTGCGCGCTTTCCGGCACGCTGAACGAACTGTTGCTGGCACGCGCGTTACAGGGCGTTGGCGGCGCGATGATGGTGCCGGTCGGCAGATTGACGGTGATGAAAATCGTACCGCGCGAGCAATATATGGCGGCGATGACCTTTGTCACGTTACCCGGTCAGGTCGGTCCGCTGCTCGGTCCGGCGCTCGGCGGTCTGCTGGTGGAGTACGCATCGTGGCACTGGATCTTTTTGATCAACATTCCGGTGGGGATTATCGGTGCGATCGCCACATTGCTGTTAATGCCGAACTACACCATGCAGACGCGGCGCTTTGATCTCTCCGGATTTTTATTGCTGGCGGTTGGCATGGCGGTATTAACCCTGGCGCTGGACGGCAGTAAAGGTACAGGTTTATCGCCGCTGACGATTGCAGGCCTGGTCGCAGTTGGCGTGGTGGCACTGGTGCTTTATCTGCTGCACGCCAGAAATAACAACCGTGCCCTGTTCAGTCTGAAACTGTTCCGTACTCGTACCTTTTCGCTGGGCCTGGCGGGGAGCTTTGCCGGACGTATTGGCAGTGGCATGTTGCCCTTTATGACACCGGTTTTCCTGCAAATTGGCCTCGGTTTCTCGCCGTTTCATGCCGGACTGATGATGATCCCGATGGTGCTTGGCAGCATGGGAATGAAGCGAATTGTGGTACAGGTGGTGAATCGCTTTGGTTATCGTCGGGTACTGGTAGCGACCACGCTGGGTCTGTCGCTGGTCACCCTGTTGTTTATGACTACCGCCCTGCTGGGCTGGTACTACGTTTTGCCGTTCGTCCTGTTTTTACAAGGGATGGTCAACTCGACGCGTTTCTCCTCCATGAACACCCTGACGCTGAAAGATCTCCCGGACAATCTGGCGAGCAGCGGCAACAGCCTGCTGTCGATGATTATGCAATTGTCGATGAGTATCGGCGTCACTATCGCCGGGCTGTTGCTGGGACTTTTTGGTTCACAGCATGTCAGCGTCGACAGCGGCACCACACAAACCGTCTTTATGTACACCTGGCTTAGCATGGCGTTGATCATCGCCCTTCCGGCGTTCATCTTTGCCAGAGTGCCGAACGATACGCATCAAAATGTAGCTA

*macB* gene sequencing fragment of K-12 strain

AGCACCGAACATTCTGACGCTGGCAGATATGAGCGCCATGCTGGTAAAAGCGCAGGTTTCTGAAGCGGATGTAATCCACCTGAAGCCGGGGCAAAAAGCCTGGTTTACGGTGCTTGGCGATCCACTGACGCGCTACGAGGGGCAAATCAAGGATGTACTACCGACGCCGGAAAAGGTTAACGACGCTATTTTCTATTACGCCCGTTTTGAAGTCCCCAACCCCAATGGTTTGCTGCGGCTGGATATGACTGCGCAAGTGCATATTCAGCTCACCGATGTGAAAAATGTGCTGACGATCCCTCTGTCGGCGTTAGGCGATCCGGTTGGCGATAATCGTTATAAAGTCAAATTGTTGCGTAATGGTGAAACACGCGAGCGTGAAGTGACGATTGGCGCACGTAACGATACCGATGTTGAGATTGTCAAAGGGCTTGAAGCGGGCGATGAAGTGGTGATTGGTGAGGCCAAACCAGGAGCTGCACAATGACGCCTTTGCTCGAATTAAAGGATATTCGTCGCAGCTATCCTGCCGGTGATGAGCAGGTTGAGGTGCTGAAGGGCATCAGCCTCGATATTTATGCGGGTGAGATGGTCGCGATTGTTGGCGCTTCGGGTTCCGGTAAATCGACCCTGATGAATATTCTCGGCTGTCTGGATAAGGCCACCAGCGGCACCTATCGCGTCGCCGGTCAGGATGTTGCCACGCTGGACGCCGATGCGCTGGCGCAACTGCGCCGCGAGCATTTCGGCTTTATTTTCCAGCGTTACCATTTGCTTTCGCATTTAACCGCCGAGCAGAACGTTGAAGTACCCGCCGTCTATGCTGGTCTTGAGCGGAAACAGCGACTGCTTCGTGCCCAGGAGTTGCTGCAACGGCTGGGGCTGGAAGACCGTACAGAGTATTATCCGGCACAGCTTTCGGGTGGTCAGCAACAGCGCGTCAGCATCGCGCGGGCATTGATGAACGGTGGTCAGGTAATTCTTGCCGATGAACCAACCGGCGCACTGGACAGCCATTCTGGCGAAGAGGTGATGGCGATCCTGCATCAGCTGCGCGATCGTGGGCATACGGTGATTATCGTCACCCACGATCCGCAGGTCGCTGCTCAGGCCGAGCGGGTGATCGAAATTCGCGACGGCGAAATTGTGCGCAATCCTCCCGCCATTGAAAAAGTGAATGTTACTGGCGGGACGGAACCTGTTGTCAACACGGTGTCTGGCTGGCGGCAGTTTGTCAGCGGTTTTAACGAGGCGCTGACGATGGCATGGCGGGCGCTGGCAGCGAATAAAATGCGTACTTTACTGACCATGCTGGGGATTATTATCGGTATTGCGTCGGTGGTTTCCATTGTCGTGGTGGGTGACGCCGCCAAACAAATGGTGCTGGCGGATATTCGTTCTATTGGTACGAATACTATTGATGTCTATCCCGGGAAAGATTTTGGCGATGACGATCCGCAATATCAGCAGGCGCTGAAGTACGACGACTTAATCGCCATCCAAAAACAACCGTGGGTCGCCTCAGCCACACCTGCCGTCTCGCAAAACCTGCGCCTGCGTTATAACAATGTTGATGTTGCTGCCAGTGCCAATGGCGTGAGCGGCGATTATTTTAATGTCTATGGCATGACCTTCAGTGAAGGAAACACCTTTAATCAGGAGCAGCTGAACGGTCGTGCGCAGGTCGTGGTTCTCGACAGTAATACTCGCCGCCAGCTTTTCCCCCATAAAGCAGATGTGGTTGGCGAGGTGATTCTGGTCGGCAATATGCCCGCCAGAGTCATTGGTGTGGCGGAAGAAAAACAGTCGATGTTTGGTAGCAGTAAAGTGCTGCGTGTCTGGCTACCTTACAGCACGATGTCCGGG

*mdtF* gene sequencing fragment of K-12 strain

CTCGCTATGCCTTCATTACCTGAAGCAGTGCAGCAGCAGGGGATTAGCGTCGATAAGTCGAGCAGTAATATCCTGATGGTAGCGGCGTTTATTTCTGATAACGGCAGCCTCAACCAGTACGATATCGCGGACTATGTAGCGTCTAATATCAAAGACCCGCTAAGCCGTACCGCGGGCGTTGGTAGCGTACAACTCTTTGGTTCCGAGTATGCCATGCGTATCTGGCTGGACCCGCAAAAACTCAATAAATATAACCTGGTACCTTCCGATGTTATTTCCCAGATTAAGGTGCAAAACAACCAGATTTCCGGTGGTCAACTGGGTGGCATGCCACAGGCGGCAGACCAGCAGCTAAACGCCTCGATCATTGTGCAGACGCGTCTGCAAACGCCGGAAGAATTTGGCAAAATCCTGTTGAAAGTTCAGCAAGATGGTTCGCAAGTGCTGCTGCGTGATGTCGCTCGCGTCGAACTTGGGGCGGAAGATTATTCCACCGTGGCACGCTATAACGGCAAACCTGCTGCCGGGATCGCCATCAAACTGGCTGCCGGAGCAAACGCCCTGGATACCTCGCGGGCAGTCAAAGAGGAACTGAACCGCTTATCAGCCTATTTCCCGGCAAGTCTGAAGACGGTTTATCCTTACGACACCACGCCGTTTATCGAAATTTCTATTCAGGAAGTTTTCAAAACACTGGTTGAGGCTATCATCCTAGTCTTCCTGGTCATGTATCTGTTTTTGCAGAATTTCCGTGCCACAATCATCCCGACGATTGCCGTACCGGTGGTTATTCTCGGGACGTTTGCGATCTTGTCGGCGGTCGGTTTCACCATCAACACGTTGACTATGTTCGGGATGGTGCTGGCGATAGGGTTACTGGTGGATGACGCCATCGTGGTGGTGGAGAACGTCGAGCGTGTCATTGCGGAAGATAAGCTACCGCCGAAGGAAGCGACGCATAAATCGATGGGGCAGATCCAACGTGCGCTGGTCGGTATTGCCGTTGTTCTTTCCGCAGTGTTTATGCCGATGGCCTTTATGAGCGGTGCAACCGGGGAGATCTACCGCCAGTTCTCCATCACGCTGATCTCCTCCATGCTGCTTTCAGTATTTGTGGCAATGAGCCTGACCCCTGCCCTGTGCGCCACCATTCTGAAAGCCGCGCCGGAAGGCGGTCACAAACCTAACGCCCTGTTCGCACGCTTCAACACGCTGTTTGAAAAATCAACTCAACACTATACCGATAGCACCCGCTCGCTGTTGCGTTGTACCGGTCGCTACATGGTGGTCTACCTGCTGATTTGCGCCGGGATGGCGGTGCTGTTCCTGCGCACGCCGACCTCTTTCTTACCAGAAGAGGATCAGGGGGTATTTATGACCACCGCGCAGTTACCTTCCGGTGCCACCATGGTTAACACCACGAAAGTGCTGCAACAGGTGACGGATTATTATCTGACTAAAGAGAAAGATAATGTCCAGTCGGTGTTTACCGTTGGCGGCTTTGGCTTCAGCGGTCAGGGGCAAAACAACGGCCTGGCGTTTATCAGTCTCAAGCCGTGGTCTGAACGTGTCGGTGAGGAAAACTCGGTTACCGCGATCATTCAGCGGGCAATGATTGCGTTAAGCAGTATCAATAAAGCCGTCGTCTTCCCGTTCAACTTACCCGCGGTGGCTGAACTGGGTACCGCGTCAGGTTTTGATATGGAACTGCTGGACAACGGTAACCTGGGGCACGAAAAACTAACCCAGGCGCGAAACGAGCTGTTATCACTGGCAGCGCAATCACCGAATCAGGTCACCGGGGTACGCCCGAACGGCCTGGAAGATACGCCGATGTTCAAAGTGAACGTCAACGCTGCGAAAGCTGAAGCGATGGGCGTGGCGCTGTCTGATATCAACCAGACAATTTCCACCGCCTTCGGCAGCAGCTACGTGAACGACTTCCTCAACCAGGGGCGGGTGAAAAAAGTGTATGTCCAGGCAGGCACGCCGTTCCGTATGTTGCCGGATAACATCAACCAATGGTATGTACGCAACGCCTCTGGCACGATGGCACCGCTTTCTGCCTACTCGTCTACCGAATGG
