# Supplementary material for: Resistance characterization and transcriptomic analysis of imipenem-induced drug resistance in Escherichia coli
Source: PeerJ. 2024 Nov 29;12:e18572. doi: 10.7717/peerj.18572 (PMC11610472; doi:10.7717/peerj.18572)
Supplement: Table S6 [file peerj-12-18572-s012.docx]

Table S6 Primers for agarose gel electrophoresis of gene knockout strains

| Genes | Primers | Product size in the WT strain (bp) | Product size in the gene knockout strain (bp) | |
| --- | --- | --- | --- | --- |
| *mdtC-1* | F: AAAGCCTGACCTCATTTGTTGG  R: GCCGCCGTTGTTGTAGTGAAT | 2072 | | 1478 |
| *mdtC-2* | F: GATCGGTATCGTGAAGAAGAAC  R: TGACTTGCCGTAGCCTTG | 1897 | | 1303 |
| *mdtD* | F: GGCTTTACAGGCGGTTCG  R: TCAGGGTGTTCATGGAGGA | 2422 | | 1522 |
| *macB* | F: TTAGGCCAGTGGGAAAGTTCG  R: GCTGCTCCTGATTAAAGGTGTT | 2651 | | 1751 |
| *mdtE* | F: TGTGCCTGTATCCCACCTTA  R: ACCTGAGTGACCGAGTCTTCTA | 1496 | | 749 |
| *mdtF* | F: GGTAGAAGACTCGGTCACTCA  R: GGAAGACGACGGCTTTATT | 1820 | | 701 |
